# Engineering Highly Reduced Molybdenum Polyoxometalates via the Incorporation of *d* and *f* Block Metal Ions

**DOI:** 10.1002/anie.202201672

**Published:** 2022-03-23

**Authors:** Eduard Garrido Ribó, Nicola L. Bell, De‐Liang Long, Leroy Cronin

**Affiliations:** ^1^ School of Chemistry The University of Glasgow Glasgow G12 8QQ UK

**Keywords:** Lanthanides, Molybdenum, Polyoxometalates, Redox Materials, Self-Assembly

## Abstract

The assembly of nanoscale polyoxometalate (POM) clusters has been dominated by the highly reduced icosahedral {Mo_132_} “browns” and the toroidal {Mo_154_} “blues” which are 45 % and 18 % reduced, respectively. We hypothesised that there is space for a greater diversity of structures in this immediate reduction zone. Here we show it is possible to make highly reduced mix‐valence POMs by presenting new classes of polyoxomolybdates: [Mo^V^_52_Mo^VI^_12_H_26_O_200_]^42−^ {Mo_64_} and [Mo^V^_40_Mo^VI^_30_H_30_O_215_]^20−^ {Mo_70_}, 81 % and 57 % reduced, respectively. The {Mo_64_} cluster archetype has a super‐cube structure and is composed of five different types of building blocks, each arranged in overlayed Archimedean or Platonic polyhedra. The {Mo_70_} cluster comprises five tripodal {Mo^V^_6_} and five tetrahedral {Mo^V^_2_Mo^VI^_2_} building blocks alternatively linked to form a loop with a pentagonal star topology. We also show how the reaction yielding the {Mo_64_} super‐cube can be used in the enrichment of lanthanides which exploit the differences in selectivity in the self‐assembly of the polyoxometalates.

## Graphical abstract

Novel types of highly reduced Mo polyoxometalates, the {Mo_64_} cube and {Mo_70_} star, are obtained through near‐hydrothermal conditions with incorporations of nickel and lanthanide ions and possess a set of building blocks different to those present in Mo blues and browns. With a few of other rare structures, this group of polyoxomolybdates is redefined as Mo reds based on the major differences in building blocks and reduction degrees.

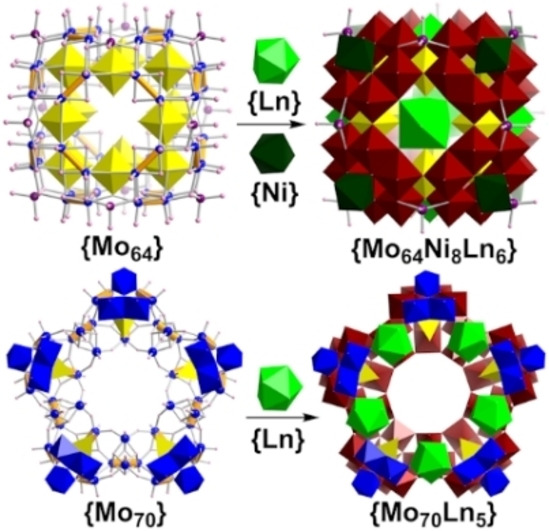

## Introduction

Exploring the assembly‐space of metal oxides is important to understand self‐assembly and discover new structural archetypes. In the case of polyoxometalates (POMs), the self‐assembly is normally critically controlled by the degree of reduction, type of anionic templates, and the nature of the cations. POMs are a diverse family of molecular metal oxides which have already been used for or have potential in catalysis,^[1]^ medicine,^[2]^ biosensors^[3]^ and materials^[4]^ by exploiting their redox, spectroscopic, photovoltaic, or magnetic properties, and selective enrichment of ions or molecules.[^5^, ^6^] Recent work demonstrated the promise of POMs in energy storage due to their ability to store multiple electrons reversibly by undergoing super‐stoichiometric reduction, accepting as many electrons as there are redox centres in the clusters.[^7^, ^8^] However, the stability of reduced POM species in the solid state remains an issue, since the reaction with oxygen is facile so POM materials that are highly reduced, and stable in air, have potential for use as very high energy density materials.

**Figure 1:**
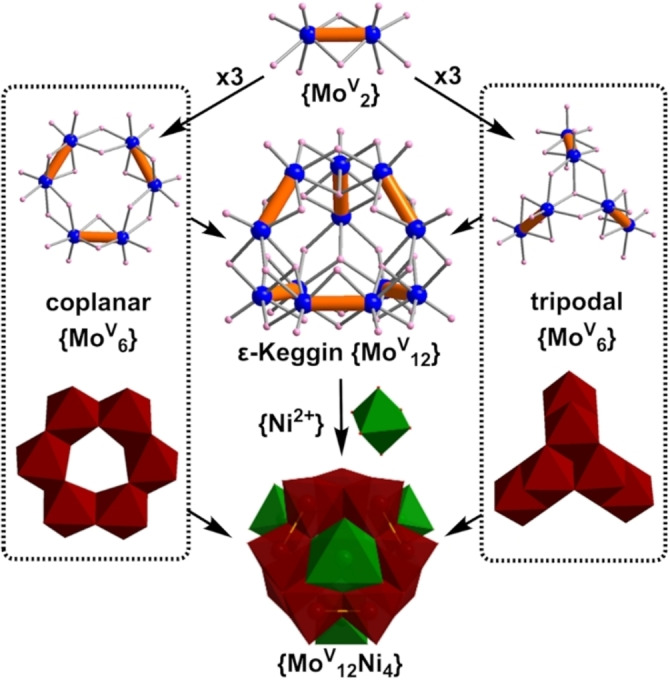
Schematic representation of the formation of the different building blocks that produce the ϵ‐Keggin anion, [Mo_12_H_12_O_40_]^8−^, and its Ni‐capped derivative structure, {Mo_12_Ni_4_}. Mo^V^: blue balls and brown polyhedra. Ni: dark green balls and polyhedra. O: pink balls.

To find or engineer more highly reduced materials we need to understand how mixed valance polyoxomolybdates can be manipulated as oligomeric assemblies of fundamental building blocks (BBs) in solution. The building blocks commonly found are the pentagonal {(Mo)Mo_5_} units, as well as the {Mo_2_} and {Mo_1_} units seen in Molybdenum blues {Mo_154_}^[9]^ or {Mo_368_}.^[10]^ The negative charge is delocalised throughout the structure, which yields the characteristic blue colour of these clusters. When the degree of reduction and pH are increased, a {Mo^V^_2_} unit is found to be a new type of building block. This basic unit having a Mo^V^−Mo^V^ bond of ≈2.5 Å (Figure 1) differs from the {Mo^VI^_2_} one, which has Mo⋅⋅⋅Mo separation larger than 3.0 Å.

This building block localizes the two reducing electrons to form the Mo^V^−Mo^V^ bond and creates a set of structures commonly known as the Molybdenum browns, due to their brown colour. Many of the other building blocks are shared between both sets of reduced molybdates, as seen in the well‐studied {Mo_132_},^[11]^ with the {(Mo)Mo_5_} pentagonal unit being key for molecular growth.^[12]^ However, there is a small number of highly reduced Mo clusters that present building blocks that do not match those of the previously mentioned sets. The {Mo_37_}^[13]^ and {Mo_42_}^[14]^ examples have been its major representatives but they have not been widely exploited to date. More recently, however, the {Mo_240_} has been a new addition to this now growing group of mix‐valence molybdates.^[15]^ Herein we report an approach to control the building blocks, and the structures formed using both *d* and *f* metal ions in the synthesis of two new classes of highly reduced, mix‐valence polyoxomolybdates: [Mo^V^_52_Mo^VI^_12_H_26_O_200_]^42−^ {Mo_64_} and [Mo^V^_40_Mo^VI^_30_H_30_O_215_]^20−^ {Mo_70_} and redefine this group of molybdates as Molybdenum reds.

## Results and Discussion

### Synthesis of Molybdenum Reds

The [Mo^V^_52_Mo^VI^_12_H_26_O_200_]^42−^ and [Mo^V^_40_Mo^VI^_30_H_30_O_215_]^20−^ cluster anions were obtained with incorporation of Ni^2+^ and a range of Ln^3+^ ions and charge balanced by Na^+^ cations as follows:

$$1.\;\mathrm{Na}{}_{9}\left[\mathrm{Mo}{}_{64}\mathrm{Ni}{}_{8}\mathrm{La}{}_{6}H{}_{26}O{}_{200}\left(H{}_{2}O\right){}_{30}\right]\left(\mathrm{ClO}{}_{4}\right)\left(H{}_{2}O\right){}_{55},\{\mathrm{Mo}{}_{64}\mathrm{Ni}{}_{8}\mathrm{La}{}_{6}\};$$

$$2.\;\mathrm{Na}{}_{9}\left[\mathrm{Mo}{}_{64}\mathrm{Ni}{}_{8}\mathrm{Ce}{}_{6}H{}_{26}O{}_{200}\left(H{}_{2}O\right){}_{30}\right]\left(\mathrm{ClO}{}_{4}\right)\left(H{}_{2}O\right){}_{55},\{\mathrm{Mo}{}_{64}\mathrm{Ni}{}_{8}\mathrm{Ce}{}_{6}\};$$

$$2{}^{'}.\;\mathrm{Na}{}_{9}\left[\mathrm{Mo}{}_{64}\mathrm{Ni}{}_{8}\mathrm{Ce}{}_{6}H{}_{26}O{}_{200}\left(H{}_{2}O\right){}_{30}\right]\left(\mathrm{ClO}{}_{4}\right)\left(H{}_{2}O\right){}_{30},\{\mathrm{Mo}{}_{64}\mathrm{Ni}{}_{8}\mathrm{Ce}{}_{6}\};$$

$$3.\;\mathrm{Na}{}_{9}\left[\mathrm{Mo}{}_{64}\mathrm{Ni}{}_{8}\mathrm{Pr}{}_{6}H{}_{26}O{}_{200}\left(H{}_{2}O\right){}_{30}\right]\left(\mathrm{ClO}{}_{4}\right)\left(H{}_{2}O\right){}_{30},\{\mathrm{Mo}{}_{64}\mathrm{Ni}{}_{8}\mathrm{Pr}{}_{6}\};$$

$$4.\;\mathrm{Na}{}_{3}\mathrm{Ni}{}_{0.5}\mathrm{Ce}\left[\mathrm{Ce}{}_{4.5}\mathrm{Mo}{}_{70}H{}_{30}O{}_{215}\right]\mathrm{Cl}{}_{0.5}\left(H{}_{2}O\right){}_{250},\;\left\{\mathrm{Mo}{}_{70}\mathrm{Ce}{}_{4}.{}_{5}\right\};$$

$$5.\;\mathrm{Na}{}_{3}\mathrm{Nd}{}_{0.67}\left[\mathrm{Nd}{}_{5}\mathrm{Mo}{}_{70}H{}_{30}O{}_{215}\right]\left(H{}_{2}O\right){}_{180},\;\left\{\mathrm{Mo}{}_{70}\mathrm{Nd}{}_{5}\right\};$$

$$6.\;\mathrm{Na}{}_{3.5}\mathrm{Sm}{}_{0.5}\left[\mathrm{Sm}{}_{5}\mathrm{Mo}{}_{70}H{}_{30}O{}_{215}\right]\left(H{}_{2}O\right){}_{250},\;\left\{\mathrm{Mo}{}_{70}\mathrm{Sm}{}_{5}\right\}.$$

**Figure 2:**
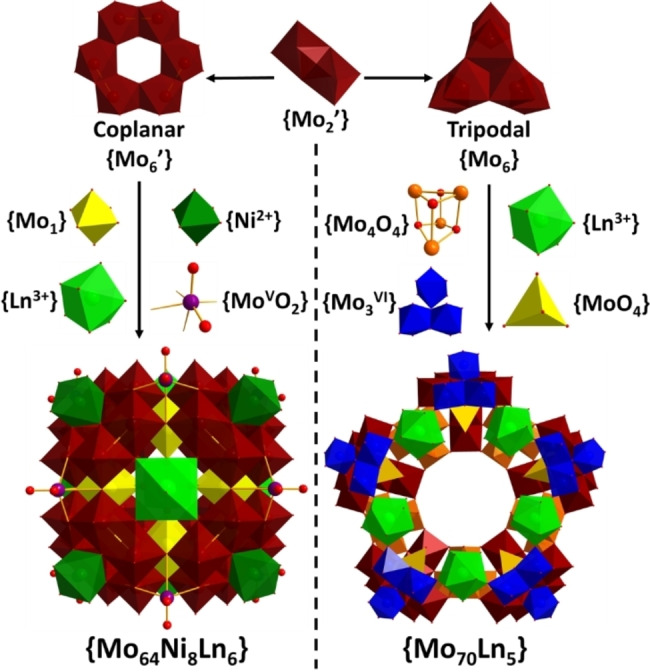
Schematic representation of the formation of compounds **1** to **6**. Left side: compounds **1**–**3**. Right side: compounds **4**–**6**. Mo^V^: maroon balls and polyhedra. {Mo^VI^}: yellow or blue polyhedra. {Mo^V^O_2_}: purple ball. Ni: dark green polyhedra. Ln: light green balls and polyhedra. O: red balls.

Compound **1** was synthesized in a one‐pot reaction by acidifying a mixture of Na_2_MoO_4_⋅2 H_2_O (0.2 mmol) and NiCl_2_⋅6 H_2_O (0.4 mmol) with HClO_4_ (6 mmol). This was followed by the addition of LaCl_3_⋅7 H_2_O (0.08 mmol) and finally N_2_H_4_⋅2 HCl (0.24 mmol) as the reducing agent. After the mixture was allowed to react in a sealed vial for ≈2 h at 90 °C, the solution changed from light green to red‐brown. At this point a base was added to raise the pH to 2.8, which caused a colour change from red‐brown to deep blue. The sample was then sonicated and flushed with argon to remove oxygen, sealed in a vial, and kept in an oven at 100 °C for 4 days resulting in the formation of single crystals of compound **1**, see Figure 2.^[16]^

Dissolution of [Mo^V^_2_O_4_(H_2_O)_4_]^2+^ seems to rapidly lead to the initial formation of the anionic ϵ‐Keggin‐type cluster [Mo^v^_12_H_12_O_40_]^8−^ (Figure 1), thereby increasing the nucleophilicity of the molybdate oxo ligands. However, this isomer of ϵ‐Keggin structure is not stable and is usually stabilized by electrophiles such as Ni^2+^ ions for charge balancing of the anionic polyoxometalate super structure. Without reducing agent, it has been reported that at low pH a solution containing Ni^2+^ and Mo^6+^ will form the Anderson‐type POM {NiMo_6_} with the Ni^2+^ ion occupying the centre of a coplanar {Mo_6_} hexagon.^[17]^

In compound **1** the Ni^2+^ ion adopts a similar position but since the current system is highly reduced and involves La^3+^ ions, a non‐Anderson‐type POM but rather a super cube cluster structure of the type [Mo_64_Ni_8_La_6_H_26_O_200_(H_2_O)_30_]^8−^ forms. We then investigated whether the same Mo/Ni‐only cube framework would form in the absence of La^3+^ ions but instead the Ni^2+^ complex of ϵ‐Keggin [{Ni(H_2_O)_3_}_4_Mo^v^_12_H_12_O_40_] {Mo_12_Ni_4_} (Figure 1) is formed under exact the same reaction conditions.^[16]^ This observation supports the hypothesis that the lanthanide(III) ions assist in the molecular growth and self‐assembly of **1**. In addition, a high ratio of La : Mo is needed to obtain **1** reliably, and in good yield, although no evidence showed that the excess lanthanide ions act as separated counter cations for the cluster. This is similar to the case previously reported for the synthesis of the {Mo_90_Ln_10_} lanthanide ring.^[18]^

This excess, in addition to an even higher pH, results in a substantial amount of precipitate in the form of La_2_O_3_⋅7 MoO_3_ and also crystals of {La_3_[LaMo_12_]}.^[19]^ However, trying to filter these side‐products before putting the samples to crystallize at high temperature failed to produce any crystals whatsoever, indicating that at high temperature the solubility of these compounds change which facilitates the formation of the cube over an extended period. After cooling to room temperature, crystals of compound **1** could be isolated from the side‐products by washing the solid with ice‐cold water and removing the precipitate in suspension while allowing the crystals to sink. Alternatively, since the crystals of compound **1** were rather big (≈100 μm), they could be selectively filtered by using a larger pore sized filter mesh which allowed the precipitate and smaller crystals to wash off while retaining compound **1**. Compounds **2** and **3** were synthesized and purified in the same manner with Ce^3+^ and Pr^3+^ replacing La^3+^ and the clusters adopt the same cubic structure as **1**. **1** and **2** are isomorphous and crystallise in similar unit cells, however **2** also forms another phase compound **2′**, which crystallizes in a unit cell isomorphous to **3**. Hence compounds **2′** and **3** share an almost identical cluster framework to **1** and **2** but they deviate slightly in the overall size of the cluster due to the smaller lanthanide ion. This is consistent with the crystal morphology observed; compounds **1** and **2** grow as large red square plates while **2′** and **3** are found as long red rods.

Compound **4** was obtained under similar reaction conditions to those for compounds **1**–**3**, with two key differences: the pH and the amount of reducing agent used. A lower pH value is needed to obtain **4** as well as a reducing stoichiometry similar to that of the final structure (i.e., 60 % reduced). That said however, where these two syntheses differ the most is on the yield and purity of the final product. Where for compounds **1** to **3** the crystals that appeared were relatively big and easy to separate from their by‐products in the reaction mixture, here the crystals of **4** were found as tiny rods in a midst of other crystals from minor molybdenum‐based salts, mostly MoO_3_,^[20]^ making the isolation of compound **4** extremely difficult. A possible reason for this could be that the overall structure may not be very stable, or rather, it may be a high energy intermediate between other, more thermodynamically stable, compounds.

The cluster [Mo_70_H_30_O_215_]^20−^ {Mo_70_} in compound **4** exhibits a pentagonal star‐like structure with building blocks similar to that of {Mo_240_} cage reported earlier, see Figure 4, and it seems possible that this compound might be an intermediate in the formation of the {Mo_240_}.^[15]^ Compounds **5** and **6** have structures derived from **4** where the Ce^3+^ are replaced by Nd^3+^ and Sm^3+^ respectively and they retain the {Mo_70_} star‐like framework but have significant differences in lanthanide ion coordination. For compound **4**, the large cerium ions are difficult to fully incorporate into the star rim resulting in the 5^th^ Ce^3+^ ion in the rim moving out of plane and bridging between two adjacent star structures. As such, the Ce^3+^ ion on this position of a crystallographic inversion centre is shared by two star molecules. In contrast, the rim lanthanides in both **5** and **6** are all within the plane and are fully occupied. Due to these differences the three compounds, **4**, **5**, and **6**, crystallise in very different crystal systems, see Table S4.

### Cluster Assembly from Building Blocks

**Figure 3:**
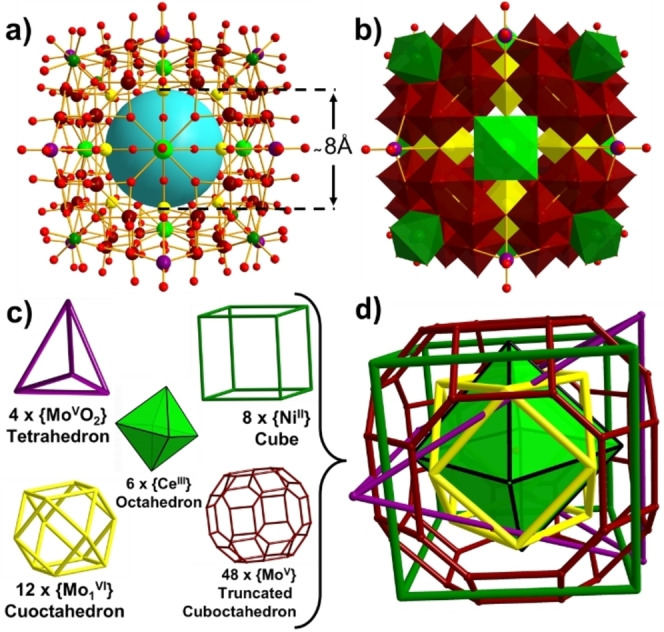
a) Ball‐and‐stick representation of the {Mo_64_Ni_8_Ln_6_} framework with the central cavity painted in light blue. b) Polyhedral representation of {Mo_64_Ni_8_Ln_6_}. c) Different geometric shapes that conform to the structure. d) Simplified metal atom based skeleton with all the overlayed geometrical shapes. Mo^V^: maroon balls and polyhedra. {Mo^VI^_1_}: yellow balls and polyhedra. {Mo^V^O_2_}: purple ball. Ni: dark green balls and polyhedra. Ln: light green balls and polyhedra. O: red balls.

Single‐crystal X‐ray structural analysis^[21]^ revealed that compounds **1**, **2**, **2′** and **3** crystallize in the tetragonal system with the space group *I*4*/m* (Table S2 and S3) and share the same main framework [Mo_64_Ni_8_Ln_6_H_26_O_200_(H_2_O)_30_]^8−^, {Mo_64_Ni_8_Ln_6_} (Ln=La, Ce and Pr), containing a [Mo^V^_52_Mo^VI^_12_H_26_O_200_]^42−^ {Mo_64_} polyoxomolybdate (Figure S3). The structure is composed by 5 types of building blocks. Firstly, 48 Mo^V^ atoms form the main framework of the structure in the shape of a truncated cuboctahedron (Red, Figure 3c, d). These Mo^V^ atoms can be divided into 8 coplanar {Mo^V^_6_} building blocks, each of which is composed by 3 Mo−Mo bonded dimers, {Mo^V^_2_}. This coplanar {Mo^V^_6_} building block can be considered as a lacunary ϵ‐Keggin polyoxomolybdate since it resembles the classic ϵ‐Keggin isomer with a tripodal {Mo^V^_6_} unit removed. Over the centre of each coplanar {Mo^V^_6_}, a {Ni^II^(H_2_O)_3_} is fitted to coordinate to three *μ*_2_‐O atoms on the {Mo^V^_6_} and form a {NiO_3_(H_2_O)_3_} moiety of C_3v_ symmetry, which resembles one of the four faces of a Ni doped ϵ‐Keggin, {Mo_12_Ni_4_}.^[16]^ Combined, these 8 Ni atoms form a cubic topology that defines the outline of the entire cluster structure. The Ni⋅⋅⋅Ni distance along the cube edge is *ca* 10.4 Å indicating a cluster size of over 1000 Å^3^ in volume. Attached to the main {Mo^V^_48_} framework, 12 {Mo^VI^} sites, each below a cube edge centre, are distributed in a cuboctahedron (yellow, Figure 3c, d) serving as joints between two adjacent {Mo^V^_6_} building blocks. 6 {Ln^III^(H_2_O)} sites, each on a face‐centre of the cube, form an octahedron (light green, Figure 3c, d). Each one of the {Ln^III^(H_2_O)} units link together 4 {Mo^V^_6_} groups as well as 4 {Mo^VI^} units by coordinating to 4 μ_2_‐O atoms each from a {Mo^V^_6_} and 4 terminal O atoms each from a {Mo^VI^} unit, leaving the final H_2_O ligand towards outside of the cluster. The coordination sphere of {LnO_8_(H_2_O)} displays regular C_4v_ symmetry. Finally, over each edge centre of the cube an additional {Mo^V^O_2_} can be found. However, the occupancy of these 12 {Mo^V^O_2_} units is only 1/3, meaning only 4 {Mo^V^O_2_} coexist in one cube which, with maximum symmetry, adopt a tetrahedral supramolecular arrangement (purple, Figure 3c, d) that crosses the entire structure. In summary, this {Mo_64_Ni_8_Ln_6_} super cube structure is comprised of 5 different overlayed and co‐centred geometrical shapes built from 5 different supramolecular building blocks: a primary truncated cuboctahedron consisting of 48 {Mo^V^}, a cube comprised of 8 {Ni^II^(H_2_O)_3_}, an octahedron of 6 {Ln^III^(H_2_O)}, a cuboctahedron made of 12 {Mo^VI^} and a disordered tetrahedron of 4 {Mo^V^O_2_} spread over 12 positions. A perchlorate anion was identified at the centre of the cubic cluster, although it was difficult to model during the structure refinement due to disorder.

**Figure 4:**
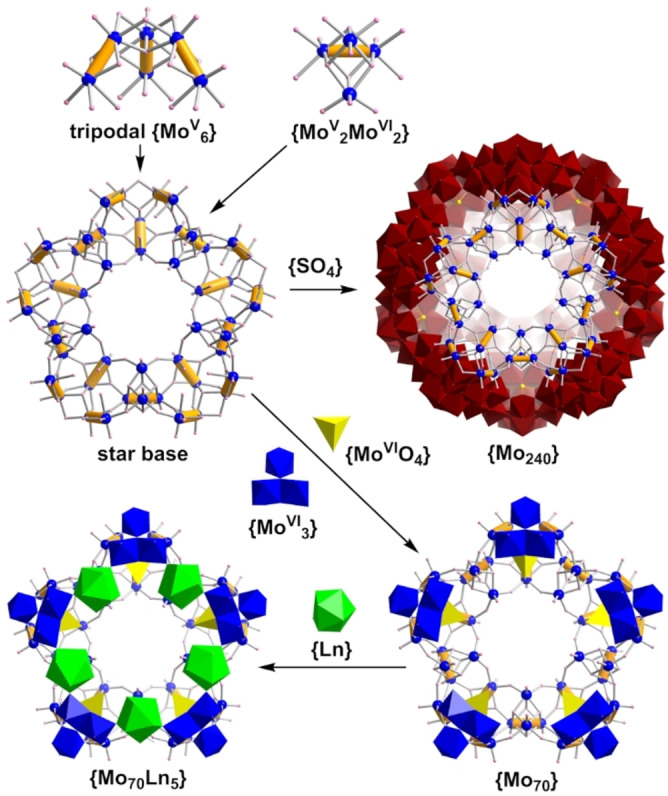
Schematical representation of the formation of the star compounds, **4**, **5** and **6**. Star base is the common part for forming {Mo_70_} and {Mo_240_}. Colour scheme is the same as that in Figure 2.

Compound **4** crystallises in the monoclinic system with C2/c space group and displays a [Mo^V^_40_Mo^VI^_30_H_30_O_215_]^20−^ {Mo_70_} cluster structure consisting of 5 {Mo_10_} units and 5 {Mo_4_} linking units. Each {Mo_10_} can be considered as a tripodal {Mo^V^_6_} unit (blue balls in Figure 4), plus a {Mo^VI^_3_} add‐on group (blue polyhedra) and a {MoO_4_} template (yellow in Figure 4) inside the tripodal {Mo^V^_6_}. The tripodal {Mo^V^_6_} building block itself can be considered a lacunary ϵ‐Keggin polyoxomolybdate by removing the coplanar {Mo^V^_6_} half of the classic ϵ‐Keggin isomer. Interestingly, the edge sharing dimers, {Mo^VI^_2_}, of the {Mo^VI^_3_} add‐on group are co‐planar with two {Mo^V^_2_} pairs of the tripodal {Mo^V^_6_} and form a {Mo^V^_4_Mo^VI^_2_} hexagonal ring with the third Mo^VI^ of the {Mo^VI^_3_} add‐on group over centre (Figure S4). The position of this Mo^VI^ site is very similar to how the Ni sits over the centre of a coplanar {Mo^V^_6_} building block in the cube structure, but with a coordination sphere of {Mo^VI^O_5_(H_2_O)} (Figure S4). As the tripodal {Mo^V^_6_} is not stable on its own, the {Mo^VI^_3_} add‐on group can be regarded as a finishing unit for the exposed two “legs” of the tripod. The tripodal {Mo^V^_6_} building block has been observed in structures like the {Mo_240_}, {Mo_37_} or {Mo_42_}, see Figure 4.

In the centre of tripodal {Mo^V^_6_} building block, a tetrahedral {MoO_4_} template also tightly contacts with the edge sharing dimer {Mo^VI^_2_}, taking a position normally occupied by a heteroatom such as P, S or Si. Five {Mo_4_}={Mo^V^_2_Mo^VI^_2_} (in tetrahedral arrangement) units connect the aforementioned {Mo_10_} building units making the main framework of the structure.^[22]^ The two molybdenum ions closest to the lanthanide ions are in the 6+ oxidation state whereas the opposite pair are in the 5+ state. Overall, the super‐star structure has an outer diameter of *ca* 23.5 Å at its most elongated points and 8.9 Å at its shortest. The inner cavity is defined by two concentric rings delineated by the {Mo_4_} units and the central {MoO_4_} tetrahedral templates, with a radius of 4.4 Å and 5.5 Å, respectively.

Compounds **5** and **6** also display the same {Mo_70_} star cluster structure however, they crystallise in different crystal systems and space groups (*P*2_1_/c for **5** and *P*2_1_/*n* for **6**). The main differences between these compounds come from the final components of the structure: lanthanide ions appended to the {Mo_70_} framework. In the case of compound **4**, 4.5 {Ce^III^(H_2_O)_*n*_} (*n*=4 to 6) per cluster can fit in the space in between two adjacent {Mo_10_} units and on top of a {Mo_4_} BB. The half equivalent comes about due to the fact that a disordered {Ce^III^(H_2_O)_*n*_} is located around a crystallographic inversion centre shared by and bridging two {Mo_70_} clusters. Between these two {Mo_70_} clusters there are other four Ce^3+^ bridges in proximate area of this inversion centre. Each Ce^3+^ typically connects to one {Mo_70_} main framework through three points: three terminal oxo each from the two neighbouring {Mo_10_} and one {Mo_4_} below. The remaining coordination sites of Ce^3+^ are occupied by water ligands or oxo ligands from nearby {Mo_70_} cluster thus forming multiple bridges between clusters. Only one of the five Ce^3+^ on the cluster does not form bridges; thus, each cluster of **4** has seven Ce^3+^ bridges between clusters that form a tightly linked 1D chain (Figure S5a).

In the case of compounds **5** and **6**, five {Ln^III^(H_2_O)_*n*_} (Ln=Nd and Sm) are found attached to the {Mo_70_} cluster. Here only two of the five {Ln^III^(H_2_O)_*n*_} are bound to oxo ligands from nearby {Mo_70_} clusters. Thus, each cluster has only four bridges forming a 1D chain (Figure S5b). Common to all three compounds is an additional {Ln^III^(H_2_O)_9_} with full or partial occupancy at the centre of the cluster. The slight differences in the packing between compounds **4**, **5** and **6** can be attributed to the different sizes of the lanthanide ions around the cores of the {Mo_16_}^[23]^ and all three different POMs have Keggin‐based repeating units.

### Molybdenum Blues, Browns and Reds

When looking at mix‐valence Mo clusters, traditionally, these have been categorised into 2 main groups. “Mo blues” are the most extensive group and also the less reduced one, with a reduction degree around 35 %. In this group we find the ring structures, the blue ball, {Mo_102_},^[24]^ and, the highest nuclearity POM known to date, the {Mo_368_} blue lemon.^[10]^ The second group, commonly denominated “Mo browns”, contains structures with higher reduced ratios (around 45 % of Mo centres reduced). Although some lesser‐known structures exist in this group like the {Mo_46_}^[25]^ and the {Mo_54_},^[26]^ the well‐known Keplerate structure, {Mo_132_}, is the main representative of this group and it has been a subject of great study for ligand exchange and host–guest chemistry.^[6]^ The main difference between Mo blues and Mo browns lies in where the electrons are located in the structures. In the Mo blues they are delocalized between several Mo atoms whereas in Mo browns we see the appearance of the {Mo^V^_2_} building block, formed by two Mo^V^, where the reducing electrons are localised and a Mo−Mo bond forms. In both the Mo blues and Mo browns, one of the most characteristic building blocks to form is the pentagonal {(Mo)Mo_5_}. This building block usually interconnects other building units and promotes molecular growth. Its versatility allows it to be present in structures as different as the {Mo_154_},^[9]^ {Mo_132_}^[11]^ and {Mo_57_}.^[13]^

Until recently, there were only three examples of mixed valence Mo compounds with a higher reducing ratio (Mo^V^ content >50 %) than the ones described here. These were, probably for a lack of a better classification, categorized as Mo browns. The first one is the ϵ‐Keggin [Mo^v^_12_H_12_O_40_]^8−^. This structure has 100 % of its Mo centres reduced but it is unstable so it does not appear on its own but rather stabilized by other metal ions like Ni^2+^, Co^2+^, Mo^VI^ and also by lanthanides.[^16^, ^23^, ^27^] A second example would be the {Mo_37_} structure.^[13]^ With a 70 % reduced ratio, this compound has been until now the highest reduced, fully inorganic Mo cluster. Finally, the {Mo_42_}, an extended structure of a ϵ‐Keggin core, represents the highest reduced Mo compound known to date (85 %) but it should be noted that it relies on organic ligands for its formation.^[14]^

**Figure 5:**
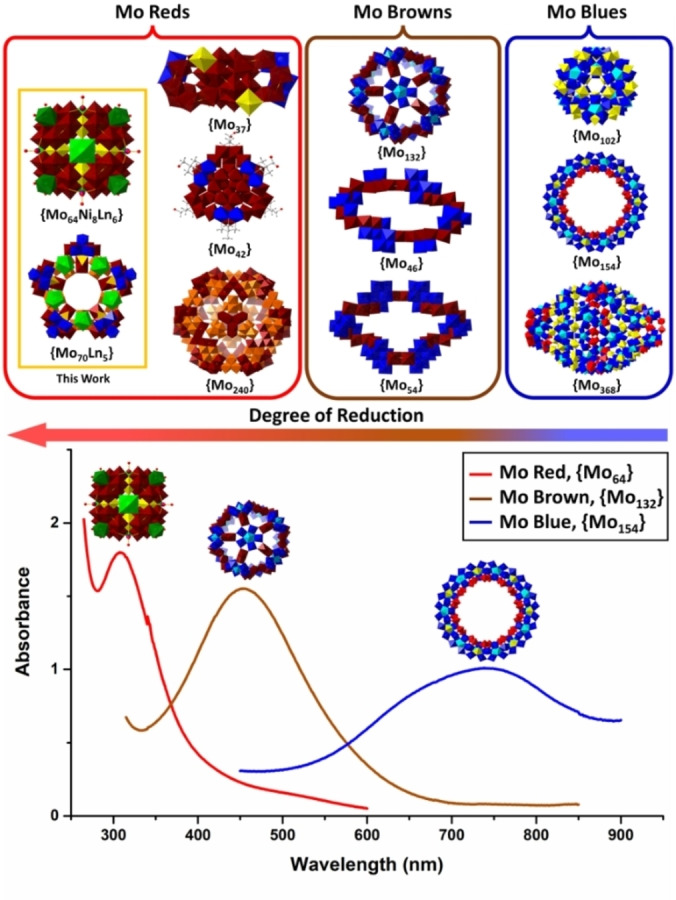
Classification of the most characteristic Mo Blues (right), Mo Browns (centre) and newly defined Mo Reds (left) structures based on their reduction degree and the characteristic UV/Vis spectrum of an example of each sub‐group. Colour scheme is the same as that in Figure 2.

In all, these three examples of highly reduced molybdates presented two key variables compared to their blue/brown cousins. The first one is the lack of the {(Mo)Mo_5_} building block. The fact, then, that this key component in molecular growth for reduced molybdates is missing suggests that the whole process of self‐assembly is completely different from the one occurring for traditional Mo blues. The second one is the lower nuclearity, as these highly reduced clusters cannot surpass 50 metal atoms in one structure compared to an average closer to 130 metal atoms for the Mo blues and browns. This however changed when Lin et al. found the {Mo_240_} cage.^[15]^ This massive cage sits at the top of the highest nuclearity Mo POMs and it is 67 % reduced. The existence of this cage implies that highly reduced Mo clusters are not limited to low nuclearities. This hypothesis has now been confirmed by the discovery of the two new different structures reported in this paper. Both the {Mo_64_} cube and the {Mo_70_} star have nuclearities higher than the average observed with reduction ratios of 81 % and 57 %, respectively (Figure 5). Table S5 summarises reported reduced polyoxomolybdates for comparison.

### Lanthanide Enrichment

When compound **1** was first encountered as {Mo_64_Ni_8_La_6_} it was hypothesized that several iso‐structures could be achieved by changing both the Ni^2+^ for a different 1^st^ row TM and the La^3+^ for other lanthanide ions. This proved to be only partially true since no compound was successfully isolated when using other TM salts. On the other hand, the cube structure was also found for Ce, compound **2**, and for Pr, compound **3**, but interestingly, for no other lanthanides. This last finding raised the question that perhaps the cube framework and its synthesis can be a means of separating different lanthanides depending on their ability to form the compound or not. With that in mind, we sought to achieve separation between close lanthanide ions and also between light and heavy lanthanides to see if there was a potential new approach.

A mixture of La and Er was chosen as a starting point since this mixture has given good results in previous work.^[18]^ Three different molar ratios were attempted to test the systems selectivity. Two of these ratios were equimolar mixtures, one using 1 equiv of each lanthanide and one using 0.5 equiv (cf. synthesis of **1**). The third one was a 3 : 1 mixture using 0.75 equiv and 0.25 equiv of La and Er, respectively. The mixture using 0.5 equiv of each lanthanide failed to produce any quality crystals but the other two did, and in comparable yield to a regular synthesis. For the 1 : 1 mixture using 1 equiv of each lanthanide, the separation factor was quite promising and comparable to previous studies. That said, the more surprising results came from the 3 : 1 mixture where a separation factor close to 26 was obtained. It was expected that with a higher content of La, the separation would be better, but such a separation factor shows the selectivity that this reaction has for light lanthanides. With these promising results achieved for one binary mixture, a set of different La based mixtures were tested to compare and test the selectivity between early and later lanthanides. Table 1 shows the different separation factors obtained using both ratios. It can be seen how lighter lanthanides (Pr, Nd, and Sm) show little to no separation, due to the similar ionic radii between lanthanides. But, as the size of the ions decreases, and only La can form the cube structure, the separation factors increase exponentially. Thus, as expected, there exists a direct correlation between the ionic radius difference and the separation factor. Moreover, when added in a 3 : 1 ratio, the selectivity of the system shows greater preference for the bigger lanthanide, leaving the smaller one in solution.

**Table 1:** Selective enrichment of light lanthanide ions from binary mixtures.

Lanthanide Mixture	Δ*r* [pm]	Separation Factor at 1 : 1 ratio	Separation Factor at 3 : 1 ratio
La/Pr	3.46	0.7	2.21
La/Nd	5.06	2.04	3.6
La/Sm	8.16	4.19	7.98
La/Ho	14.16	23.37	34.51
La/Er	15.20	8.25	25.56
La/Yb	17.16	55.20	34.99

## Conclusion

To conclude, we have synthesised highly reduced cube‐shaped polyoxometalates, {Mo_64_Ni_8_Ln_6_} (**1**–**3**), through near hydrothermal conditions and using lanthanides (La, Ce and Pr) as molecular growth agents. Strongly acidifying the reaction mixture in the presence of reducing agent promotes the reduction of the molybdenum centres from Mo^VI^ to Mo^V^, which form dimeric {Mo_2_^V^} building blocks. These can come together into Keggin‐like structural units, which will control the self‐assembly process. In addition, through similar synthetic conditions a star‐shape POM framework, {Mo_70_Ln_5‐6_} (Ln=Ce, Nd, Sm, **4**–**6**) has been isolated. This structure is believed to be a high‐energy intermediate of the recently reported {Mo_240_} cage. Its discovery has been a means to re‐evaluate the {Mo_240_} and its formation, establishing tetrahedral Mo units as templates as opposed to SO_3_^2−^/SO_4_^2−^. The cube and star are two new additions to the now growing set of structures we here define as Mo reds. The differences in the morphology and characteristic building blocks between Mo blues, browns and these new set of highly reduced polyoxomolybdates has led us to naturally classify them as a subspecies of mixed‐valence Mo‐based POMs. In addition, the cube structure has proven to be highly selective towards lanthanide size only incorporating the larger ones in its framework. We have exploited this fact through lanthanide enrichment tests where we can draw a direct correlation between the difference in ionic radii of the lanthanides in the mixture and their separation factors. The positive results obtained in these tests confirm the great potential of using self‐assembly in lanthanide separation. In conclusion, this work demonstrated the first example of incorporating different types of transition metal ions to produce high nuclearity Mo reds. We predict that there are still plenty of interesting reduced POMs that will be accessed by use of other combination of metal ions as templates and charger balancers. These materials will be of high interest as they will combine the properties of highly reduced POMs and the metal ions themselves.

## Conflict of interest

The authors declare no conflict of interest.

1

## Supporting information

As a service to our authors and readers, this journal provides supporting information supplied by the authors. Such materials are peer reviewed and may be re‐organized for online delivery, but are not copy‐edited or typeset. Technical support issues arising from supporting information (other than missing files) should be addressed to the authors.

Supporting Information

Supporting Information

Supporting Information

Supporting Information

Supporting Information

Supporting Information

Supporting Information

Supporting Information

Supporting Information

Supporting Information

Supporting Information

Supporting Information

Supporting Information

Supporting Information

Supporting Information

## Data Availability

The data that support the findings of this study are available in the supplementary material of this article.
