# Supplementary material for: Engineering Highly Reduced Molybdenum Polyoxometalates via the Incorporation of d and f Block Metal Ions
Source: Angew Chem Int Ed Engl. 2022 Mar 23;61(21):e202201672. doi: 10.1002/anie.202201672 (PMC9401863; doi:10.1002/anie.202201672)

checkCIF/PLATON report

Structure factors have been supplied for datablock(s) edu7371_sq

THIS REPORT IS FOR GUIDANCE ONLY. IF USED AS PART OF A REVIEW PROCEDURE FOR PUBLICATION, IT SHOULD NOT REPLACE THE EXPERTISE OF AN EXPERIENCED CRYSTALLOGRAPHIC REFEREE.

No syntax errors found. CIF dictionary Interpreting this report

Datablock: edu7371_sq

```
Bond precision:  La- O = 0.0034 A         Wavelength=0.71073

Cell:            a=24.4208(2)     b=24.4208(2)     c=22.8385(2)
                 alpha=90         beta=90          gamma=90
Temperature:     150 K
```

	Calculated	Reported
Volume	13620.3(3)	13620.3(3)
Space group	I 4/m	I 4/m
Hall group	-I 4	-I 4
Moiety formula	La6 Mo64 Ni8 O230, Cl O2.62, 28.867(O) [+ solvent]	?
Sum formula	Cl La6 Mo64 Ni8 O261.49 [+ solvent]	Cl H196 La6 Mo64 Na9 Ni8 O289
Mr	11662.33	12507.22
Dx,g cm-3	2.844	3.050
Z	2	2
Mu (mm-1)	4.407	4.437
F000	10725.7	11756.0
F000’	10512.71	
h,k,lmax	30,30,28	30,30,28
Nref	6893	6883
Tmin,Tmax	0.766,0.915	0.641,1.000
Tmin’	0.394	

```
Correction method= # Reported T Limits: Tmin=0.641 Tmax=1.000
AbsCorr = GAUSSIAN

Data completeness= 0.999          Theta(max)= 25.999

R(reflections)= 0.0243( 6650)     wR2(reflections)= 0.0713( 6883)

S = 1.085                   Npar= 422
```

The following ALERTS were generated. Each ALERT has the format
test-name_ALERT_alert-type_alert-level.
Click on the hyperlinks for more details of the test.

Alert level C

```
PLAT202_ALERT_3_C Isotropic non-H Atoms in Anion/Solvent .........          2 Check
              O36     O37
PLAT910_ALERT_3_C Missing # of FCF Reflection(s) Below Theta(Min).          7 Note
PLAT911_ALERT_3_C Missing FCF Refl Between Thmin & STh/L=    0.600          3 Report
PLAT918_ALERT_3_C Reflection(s) with I(obs) much Smaller I(calc) .          5 Check
PLAT975_ALERT_2_C Check Calcd Resid. Dens.  0.77A   From O46             1.25 eA-3
PLAT975_ALERT_2_C Check Calcd Resid. Dens.  0.80A   From O38             1.08 eA-3
PLAT975_ALERT_2_C Check Calcd Resid. Dens.  0.73A   From O47             0.82 eA-3
PLAT975_ALERT_2_C Check Calcd Resid. Dens.  0.56A   From O43             0.79 eA-3
```

Alert level G

```
FORMU01_ALERT_2_G  There is a discrepancy between the atom counts in the
            _chemical_formula_sum and the formula from the _atom_site* data.
            Atom count from _chemical_formula_sum:H196 Cl1 La6 Mo64 Na9 Ni8 O289
            Atom count from the _atom_site data:  Cl1 La6 Mo64.00460 Ni8 O261.4860
CELLZ01_ALERT_1_G Difference between formula and atom_site contents detected.
CELLZ01_ALERT_1_G ALERT: Large difference may be due to a
            symmetry error - see SYMMG tests
           From the CIF: _cell_formula_units_Z    2
           From the CIF: _chemical_formula_sum  Cl H196 La6 Mo64 Na9 Ni8 O289
           TEST: Compare cell contents of formula and atom_site data

           atom    Z*formula  cif sites diff
           Cl         2.00      2.00    0.00
           H        392.00      0.00  392.00
           La        12.00     12.00    0.00
           Mo       128.00    128.00    0.00
           Na        18.00      0.00   18.00
           Ni        16.00     16.00    0.00
           O        578.00    522.97   55.03
PLAT041_ALERT_1_G Calc. and Reported SumFormula    Strings  Differ     Please Check
PLAT083_ALERT_2_G SHELXL Second Parameter in WGHT  Unusually Large     118.42 Why ?
PLAT143_ALERT_4_G s.u. on c - Axis Small or Missing .............    0.00020 Ang.
PLAT172_ALERT_4_G The CIF-Embedded .res File Contains DFIX Records          1 Report
PLAT300_ALERT_4_G Atom Site Occupancy of Mo9        Constrained at     0.3333 Check
PLAT300_ALERT_4_G Atom Site Occupancy of Mo10       Constrained at     0.3333 Check
PLAT300_ALERT_4_G Atom Site Occupancy of O32        Constrained at     0.3333 Check
PLAT300_ALERT_4_G Atom Site Occupancy of O33        Constrained at     0.3333 Check
PLAT300_ALERT_4_G Atom Site Occupancy of O34        Constrained at     0.3333 Check
PLAT300_ALERT_4_G Atom Site Occupancy of O45        Constrained at     0.3333 Check
PLAT300_ALERT_4_G Atom Site Occupancy of O39        Constrained at       0.25 Check
PLAT300_ALERT_4_G Atom Site Occupancy of O41        Constrained at        0.1 Check
PLAT300_ALERT_4_G Atom Site Occupancy of O42        Constrained at      0.105 Check
PLAT300_ALERT_4_G Atom Site Occupancy of O35        Constrained at        0.5 Check
PLAT300_ALERT_4_G Atom Site Occupancy of O35'       Constrained at        0.5 Check
PLAT300_ALERT_4_G Atom Site Occupancy of O36        Constrained at     0.6667 Check
PLAT300_ALERT_4_G Atom Site Occupancy of O37        Constrained at     0.6667 Check
PLAT300_ALERT_4_G Atom Site Occupancy of O38        Constrained at     0.3333 Check
PLAT300_ALERT_4_G Atom Site Occupancy of O43        Constrained at        0.4 Check
PLAT300_ALERT_4_G Atom Site Occupancy of O44        Constrained at       0.35 Check
PLAT300_ALERT_4_G Atom Site Occupancy of O44'       Constrained at        0.4 Check
PLAT300_ALERT_4_G Atom Site Occupancy of O46        Constrained at       0.25 Check
PLAT300_ALERT_4_G Atom Site Occupancy of O47        Constrained at        0.2 Check
```

```
PLAT301_ALERT_3_G Main Residue  Disorder ..............(Resd  1  )         5% Note
PLAT302_ALERT_4_G Anion/Solvent/Minor-Residue Disorder (Resd  2  )        78% Note
PLAT302_ALERT_4_G Anion/Solvent/Minor-Residue Disorder (Resd  3  )       100% Note
PLAT302_ALERT_4_G Anion/Solvent/Minor-Residue Disorder (Resd  4  )       100% Note
PLAT302_ALERT_4_G Anion/Solvent/Minor-Residue Disorder (Resd  5  )       100% Note
PLAT302_ALERT_4_G Anion/Solvent/Minor-Residue Disorder (Resd  6  )       100% Note
PLAT302_ALERT_4_G Anion/Solvent/Minor-Residue Disorder (Resd  7  )       100% Note
PLAT302_ALERT_4_G Anion/Solvent/Minor-Residue Disorder (Resd  8  )       100% Note
PLAT302_ALERT_4_G Anion/Solvent/Minor-Residue Disorder (Resd  9  )       100% Note
PLAT302_ALERT_4_G Anion/Solvent/Minor-Residue Disorder (Resd 10  )       100% Note
PLAT302_ALERT_4_G Anion/Solvent/Minor-Residue Disorder (Resd 11  )       100% Note
PLAT302_ALERT_4_G Anion/Solvent/Minor-Residue Disorder (Resd 12  )       100% Note
PLAT304_ALERT_4_G Non-Integer Number of Atoms in ..... (Resd  1  )     269.50 Check
PLAT304_ALERT_4_G Non-Integer Number of Atoms in ..... (Resd  2  )       3.62 Check
PLAT304_ALERT_4_G Non-Integer Number of Atoms in ..... (Resd  3  )       0.50 Check
PLAT304_ALERT_4_G Non-Integer Number of Atoms in ..... (Resd  4  )       0.50 Check
PLAT304_ALERT_4_G Non-Integer Number of Atoms in ..... (Resd  5  )       0.33 Check
PLAT304_ALERT_4_G Non-Integer Number of Atoms in ..... (Resd  6  )       0.67 Check
PLAT304_ALERT_4_G Non-Integer Number of Atoms in ..... (Resd  7  )       0.33 Check
PLAT304_ALERT_4_G Non-Integer Number of Atoms in ..... (Resd  8  )       0.20 Check
PLAT304_ALERT_4_G Non-Integer Number of Atoms in ..... (Resd  9  )       0.35 Check
PLAT304_ALERT_4_G Non-Integer Number of Atoms in ..... (Resd 10  )       0.40 Check
PLAT304_ALERT_4_G Non-Integer Number of Atoms in ..... (Resd 11  )       0.13 Check
PLAT304_ALERT_4_G Non-Integer Number of Atoms in ..... (Resd 12  )       0.20 Check
PLAT311_ALERT_2_G Isolated Disordered Oxygen Atom (No H’s ?) .....        O39 Check
PLAT311_ALERT_2_G Isolated Disordered Oxygen Atom (No H’s ?) .....        O41 Check
PLAT311_ALERT_2_G Isolated Disordered Oxygen Atom (No H’s ?) .....        O42 Check
PLAT311_ALERT_2_G Isolated Disordered Oxygen Atom (No H’s ?) .....        O35 Check
PLAT311_ALERT_2_G Isolated Disordered Oxygen Atom (No H’s ?) .....       O35’ Check
PLAT311_ALERT_2_G Isolated Disordered Oxygen Atom (No H’s ?) .....        O36 Check
PLAT311_ALERT_2_G Isolated Disordered Oxygen Atom (No H’s ?) .....        O37 Check
PLAT311_ALERT_2_G Isolated Disordered Oxygen Atom (No H’s ?) .....        O38 Check
PLAT311_ALERT_2_G Isolated Disordered Oxygen Atom (No H’s ?) .....        O43 Check
PLAT311_ALERT_2_G Isolated Disordered Oxygen Atom (No H’s ?) .....        O44 Check
PLAT311_ALERT_2_G Isolated Disordered Oxygen Atom (No H’s ?) .....       O44’ Check
PLAT311_ALERT_2_G Isolated Disordered Oxygen Atom (No H’s ?) .....        O46 Check
PLAT311_ALERT_2_G Isolated Disordered Oxygen Atom (No H’s ?) .....        O47 Check
PLAT606_ALERT_4_G Solvent Accessible VOID(S) in Structure ........          ! Info
PLAT790_ALERT_4_G Centre of Gravity not Within Unit Cell: Resd.  #          6 Note
              O
PLAT790_ALERT_4_G Centre of Gravity not Within Unit Cell: Resd.  #          7 Note
              O
PLAT794_ALERT_5_G Tentative Bond Valency for La1       (III)     .       3.10 Info
PLAT794_ALERT_5_G Tentative Bond Valency for La2       (III)     .       3.09 Info
PLAT794_ALERT_5_G Tentative Bond Valency for Mo7       (VI)      .       6.05 Info
PLAT794_ALERT_5_G Tentative Bond Valency for Mo8       (VI)      .       6.08 Info
PLAT794_ALERT_5_G Tentative Bond Valency for Ni1       (II)      .       2.03 Info
PLAT869_ALERT_4_G ALERTS Related to the Use of SQUEEZE Suppressed           ! Info
PLAT933_ALERT_2_G Number of OMIT Records in Embedded .res File ...          3 Note
PLAT965_ALERT_2_G The SHELXL WEIGHT Optimisation has not Converged     Please Check
```

---

   0 **ALERT level A** = Most likely a serious problem - resolve or explain
   0 **ALERT level B** = A potentially serious problem, consider carefully
   8 **ALERT level C** = Check. Ensure it is not caused by an omission or oversight
  74 **ALERT level G** = General information/check it is not something unexpected

```
   3 ALERT type 1 CIF construction/syntax error, inconsistent or missing data
  21 ALERT type 2 Indicator that the structure model may be wrong or deficient
   5 ALERT type 3 Indicator that the structure quality may be low
  48 ALERT type 4 Improvement, methodology, query or suggestion
   5 ALERT type 5 Informative message, check
```

It is advisable to attempt to resolve as many as possible of the alerts in all categories. Often the minor alerts point to easily fixed oversights, errors and omissions in your CIF or refinement strategy, so attention to these fine details can be worthwhile. In order to resolve some of the more serious problems it may be necessary to carry out additional measurements or structure refinements. However, the purpose of your study may justify the reported deviations and the more serious of these should normally be commented upon in the discussion or experimental section of a paper or in the "special_details" fields of the CIF. checkCIF was carefully designed to identify outliers and unusual parameters, but every test has its limitations and alerts that are not important in a particular case may appear. Conversely, the absence of alerts does not guarantee there are no aspects of the results needing attention. It is up to the individual to critically assess their own results and, if necessary, seek expert advice.

Publication of your CIF in IUCr journals

A basic structural check has been run on your CIF. These basic checks will be run on all CIFs submitted for publication in IUCr journals (*Acta Crystallographica*, *Journal of Applied Crystallography*, *Journal of Synchrotron Radiation*); however, if you intend to submit to *Acta Crystallographica Section C* or *E* or *IUCrData*, you should make sure that full publication checks are run on the final version of your CIF prior to submission.

Publication of your CIF in other journals

Please refer to the *Notes for Authors* of the relevant journal for any special instructions relating to CIF submission.

PLATON version of 03/06/2021; check.def file version of 02/06/2021

Datablock edu7371_sq - ellipsoid plot

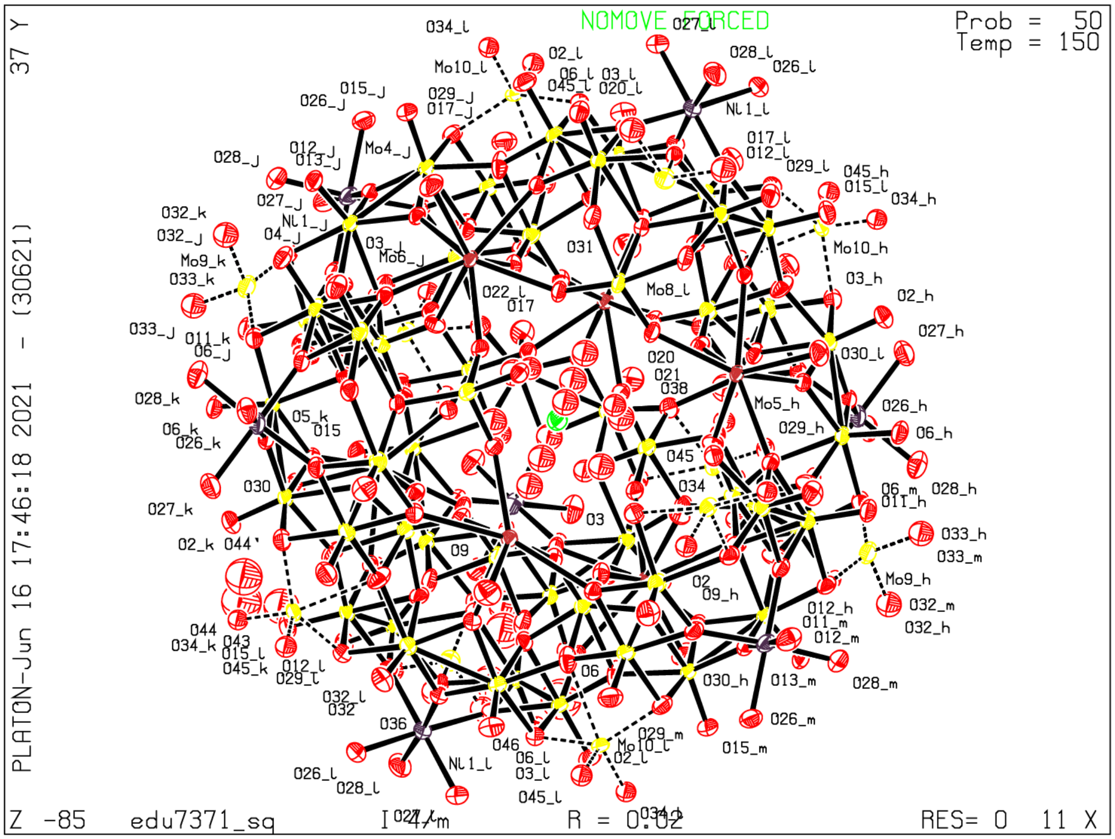

Supplement: Supplementary file 2 — Supporting Information [file ANIE-61-0-s007.pdf]
