# Supplementary material for: Engineering Highly Reduced Molybdenum Polyoxometalates via the Incorporation of d and f Block Metal Ions
Source: Angew Chem Int Ed Engl. 2022 Mar 23;61(21):e202201672. doi: 10.1002/anie.202201672 (PMC9401863; doi:10.1002/anie.202201672)

checkCIF/PLATON report

Structure factors have been supplied for datablock(s) edu6764_sq

THIS REPORT IS FOR GUIDANCE ONLY. IF USED AS PART OF A REVIEW PROCEDURE FOR PUBLICATION, IT SHOULD NOT REPLACE THE EXPERTISE OF AN EXPERIENCED CRYSTALLOGRAPHIC REFEREE.

No syntax errors found. CIF dictionary Interpreting this report

Datablock: edu6764_sq

```
Bond precision:  Ce- O = 0.0042 A        Wavelength=0.71073

Cell:            a=25.7249(2)      b=25.7249(2)      c=18.9493(2)
                 alpha=90          beta=90           gamma=90
Temperature:     150 K
```

	Calculated	Reported
Volume	12540.1(2)	12540.1(2)
Space group	I 4/m	I 4/m
Hall group	-I 4	-I 4
Moiety formula	Ce12 Mo128 Ni16 O463.20, 0.3(O16), 16(O0.60), 0.8(Cl), 25.761(O	?
Sum formula	Ce12 Cl0.80 Mo128 Na4 Ni16 O503.36 [+ solvent]	Ce6 H146 Cl Mo64 Na9 Ni8 O264
Mr	23074.80	12064.08
Dx,g cm-3	3.056	3.195
Z	1	2
Mu (mm-1)	4.846	4.873
F000	10604.4	11268.0
F000’	10391.61	
h,k,lmax	31,31,23	31,31,23
Nref	6366	6357
Tmin,Tmax	0.620,0.784	0.923,1.000
Tmin’	0.608	

```
Correction method= # Reported T Limits: Tmin=0.923 Tmax=1.000
AbsCorr = MULTI-SCANS

Data completeness= 0.999           Theta(max)= 25.990

R(reflections)= 0.0280( 5697)      wR2(reflections)= 0.0789( 6357)

S = 1.042                  Npar= 420
```

The following ALERTS were generated. Each ALERT has the format
test-name_ALERT_alert-type_alert-level.
Click on the hyperlinks for more details of the test.

Alert level B

```
PLAT975_ALERT_2_B Check Calcd Resid. Dens.  0.79A   From O46            1.75 eA-3
```

Author Response: Heavily disordered solvent area with electron density residuals that are not modelled.

Alert level C

```
PLAT077_ALERT_4_C Unitcell Contains Non-integer Number of Atoms ..     Please Check
PLAT202_ALERT_3_C Isotropic non-H Atoms in Anion/Solvent .........          2 Check
              O36     O37
PLAT910_ALERT_3_C Missing # of FCF Reflection(s) Below Theta(Min).          6 Note
PLAT911_ALERT_3_C Missing FCF Refl Between Thmin & STh/L=    0.600          3 Report
PLAT975_ALERT_2_C Check Calcd Resid. Dens.  0.68A   From O46            1.34 eA-3
```

Author Response: Heavily disordered solvent area with electron density residuals that are not modelled.

```
PLAT975_ALERT_2_C Check Calcd Resid. Dens.  0.49A   From O46            1.26 eA-3
```

Author Response: Heavily disordered solvent area with electron density residuals that are not modelled.

```
PLAT975_ALERT_2_C Check Calcd Resid. Dens.  0.53A   From O35            1.06 eA-3
```

Author Response: Heavily disordered solvent area with electron density residuals that are not modelled.

```
PLAT975_ALERT_2_C Check Calcd Resid. Dens.  0.59A   From O35            1.01 eA-3
```

Author Response: Heavily disordered solvent area with electron density residuals that are not modelled.

```
PLAT975_ALERT_2_C Check Calcd Resid. Dens.  0.94A   From O47            0.92 eA-3
```

Author Response: Heavily disordered solvent area with electron density residuals that are not modelled.

```
PLAT976_ALERT_2_C Check Calcd Resid. Dens.  0.44A   From O41'          -0.43 eA-3
```

Alert level G

```
FORMU01_ALERT_2_G  There is a discrepancy between the atom counts in the
            _chemical_formula_sum and the formula from the _atom_site* data.
```

```
            Atom count from _chemical_formula_sum:H146 Ce6 Cl1 Mo64 Na9 Ni8 O264
            Atom count from the _atom_site data:  Ce6 Cl0.4 Mo64.00460 Na2 Ni8 O25
ABSTY01_ALERT_1_G  Extra text has been found in the _exptl_absorpt_correction_type
            field, which should be only a single keyword. A literature
            citation should be included in the _exptl_absorpt_process_details
            field.
CELLZ01_ALERT_1_G Difference between formula and atom_site contents detected.
CELLZ01_ALERT_1_G ALERT: Large difference may be due to a
            symmetry error - see SYMMG tests
           From the CIF: _cell_formula_units_Z    2
           From the CIF: _chemical_formula_sum  Ce6 H146 Cl Mo64 Na9 Ni8 O264
           TEST: Compare cell contents of formula and atom_site data

           atom    Z*formula  cif sites diff
           Ce        12.00     12.00    0.00
           H        292.00      0.00  292.00
           Cl         2.00      0.80    1.20
           Mo       128.00    128.00    0.00
           Na        18.00      4.00   14.00
           Ni        16.00     16.00    0.00
           O        528.00    503.36   24.64
PLAT041_ALERT_1_G Calc. and Reported SumFormula    Strings  Differ     Please Check
PLAT045_ALERT_1_G Calculated and Reported Z Differ by a Factor ...       0.50 Check
PLAT083_ALERT_2_G SHELXL Second Parameter in WGHT  Unusually Large     158.13 Why ?
PLAT300_ALERT_4_G Atom Site Occupancy of Mo9        Constrained at     0.3333 Check
PLAT300_ALERT_4_G Atom Site Occupancy of Mo10       Constrained at     0.3333 Check
PLAT300_ALERT_4_G Atom Site Occupancy of O27        Constrained at     0.3333 Check
PLAT300_ALERT_4_G Atom Site Occupancy of O28        Constrained at     0.3333 Check
PLAT300_ALERT_4_G Atom Site Occupancy of O29        Constrained at     0.3333 Check
PLAT300_ALERT_4_G Atom Site Occupancy of O30        Constrained at     0.3333 Check
PLAT300_ALERT_4_G Atom Site Occupancy of O44        Constrained at        0.4 Check
PLAT300_ALERT_4_G Atom Site Occupancy of O45        Constrained at        0.3 Check
PLAT300_ALERT_4_G Atom Site Occupancy of O45’       Constrained at        0.3 Check
PLAT300_ALERT_4_G Atom Site Occupancy of O43        Constrained at        0.4 Check
PLAT300_ALERT_4_G Atom Site Occupancy of O43’       Constrained at        0.2 Check
PLAT300_ALERT_4_G Atom Site Occupancy of Cl1        Constrained at        0.4 Check
PLAT300_ALERT_4_G Atom Site Occupancy of O36        Constrained at     0.6667 Check
PLAT300_ALERT_4_G Atom Site Occupancy of O37        Constrained at     0.6667 Check
PLAT300_ALERT_4_G Atom Site Occupancy of O41’       Constrained at       0.32 Check
PLAT300_ALERT_4_G Atom Site Occupancy of O41"       Constrained at       0.16 Check
PLAT300_ALERT_4_G Atom Site Occupancy of O44’       Constrained at        0.4 Check
PLAT300_ALERT_4_G Atom Site Occupancy of O46        Constrained at        0.3 Check
PLAT300_ALERT_4_G Atom Site Occupancy of O47        Constrained at        0.2 Check
PLAT300_ALERT_4_G Atom Site Occupancy of Na1        Constrained at       0.34 Check
PLAT300_ALERT_4_G Atom Site Occupancy of Na1’       Constrained at       0.16 Check
PLAT301_ALERT_3_G Main Residue  Disorder .............(Resd  1  )         6% Note
PLAT302_ALERT_4_G Anion/Solvent/Minor-Residue Disorder (Resd  2  )       100% Note
PLAT302_ALERT_4_G Anion/Solvent/Minor-Residue Disorder (Resd  3  )       100% Note
PLAT302_ALERT_4_G Anion/Solvent/Minor-Residue Disorder (Resd  4  )       100% Note
PLAT302_ALERT_4_G Anion/Solvent/Minor-Residue Disorder (Resd  5  )       100% Note
PLAT302_ALERT_4_G Anion/Solvent/Minor-Residue Disorder (Resd  6  )       100% Note
PLAT302_ALERT_4_G Anion/Solvent/Minor-Residue Disorder (Resd  7  )       100% Note
PLAT302_ALERT_4_G Anion/Solvent/Minor-Residue Disorder (Resd  8  )       100% Note
PLAT302_ALERT_4_G Anion/Solvent/Minor-Residue Disorder (Resd  9  )       100% Note
PLAT302_ALERT_4_G Anion/Solvent/Minor-Residue Disorder (Resd 10  )       100% Note
PLAT302_ALERT_4_G Anion/Solvent/Minor-Residue Disorder (Resd 11  )       100% Note
PLAT302_ALERT_4_G Anion/Solvent/Minor-Residue Disorder (Resd 12  )       100% Note
PLAT302_ALERT_4_G Anion/Solvent/Minor-Residue Disorder (Resd 13  )       100% Note
PLAT304_ALERT_4_G Non-Integer Number of Atoms in ..... (Resd  1  )     270.90 Check
PLAT304_ALERT_4_G Non-Integer Number of Atoms in ..... (Resd  2  )       1.50 Check
PLAT304_ALERT_4_G Non-Integer Number of Atoms in ..... (Resd  3  )       0.60 Check
PLAT304_ALERT_4_G Non-Integer Number of Atoms in ..... (Resd  4  )       0.05 Check
```

```
PLAT304_ALERT_4_G Non-Integer Number of Atoms in ..... (Resd  5  )       0.33 Check
PLAT304_ALERT_4_G Non-Integer Number of Atoms in ..... (Resd  6  )       0.67 Check
PLAT304_ALERT_4_G Non-Integer Number of Atoms in ..... (Resd  7  )       0.08 Check
PLAT304_ALERT_4_G Non-Integer Number of Atoms in ..... (Resd  8  )       0.08 Check
PLAT304_ALERT_4_G Non-Integer Number of Atoms in ..... (Resd  9  )       0.20 Check
PLAT304_ALERT_4_G Non-Integer Number of Atoms in ..... (Resd 10  )       0.15 Check
PLAT304_ALERT_4_G Non-Integer Number of Atoms in ..... (Resd 11  )       0.10 Check
PLAT304_ALERT_4_G Non-Integer Number of Atoms in ..... (Resd 12  )       0.17 Check
PLAT304_ALERT_4_G Non-Integer Number of Atoms in ..... (Resd 13  )       0.08 Check
PLAT311_ALERT_2_G Isolated Disordered Oxygen Atom (No H’s ?) .....        O36 Check
PLAT311_ALERT_2_G Isolated Disordered Oxygen Atom (No H’s ?) .....        O37 Check
PLAT311_ALERT_2_G Isolated Disordered Oxygen Atom (No H’s ?) .....       O41’ Check
PLAT311_ALERT_2_G Isolated Disordered Oxygen Atom (No H’s ?) .....       O41" Check
PLAT311_ALERT_2_G Isolated Disordered Oxygen Atom (No H’s ?) .....       O44’ Check
PLAT311_ALERT_2_G Isolated Disordered Oxygen Atom (No H’s ?) .....        O46 Check
PLAT311_ALERT_2_G Isolated Disordered Oxygen Atom (No H’s ?) .....        O47 Check
PLAT606_ALERT_4_G Solvent Accessible VOID(S) in Structure ........          ! Info
PLAT794_ALERT_5_G Tentative Bond Valency for Ce1       (III)     .       3.13 Info
PLAT794_ALERT_5_G Tentative Bond Valency for Ce2       (III)     .       2.86 Info
PLAT794_ALERT_5_G Tentative Bond Valency for Mo5       (VI)      .       6.07 Info
PLAT794_ALERT_5_G Tentative Bond Valency for Mo6       (VI)      .       6.07 Info
PLAT794_ALERT_5_G Tentative Bond Valency for Ni1       (II)      .       2.00 Info
PLAT869_ALERT_4_G ALERTS Related to the Use of SQUEEZE Suppressed           ! Info
PLAT933_ALERT_2_G Number of OMIT Records in Embedded .res File ...          3 Note
PLAT965_ALERT_2_G The SHELXL WEIGHT Optimisation has not Converged     Please Check
```

---

0 **ALERT level A** = Most likely a serious problem - resolve or explain
1 **ALERT level B** = A potentially serious problem, consider carefully
10 **ALERT level C** = Check. Ensure it is not caused by an omission or oversight
70 **ALERT level G** = General information/check it is not something unexpected

5 ALERT type 1 CIF construction/syntax error, inconsistent or missing data
18 ALERT type 2 Indicator that the structure model may be wrong or deficient
4 ALERT type 3 Indicator that the structure quality may be low
49 ALERT type 4 Improvement, methodology, query or suggestion
5 ALERT type 5 Informative message, check

It is advisable to attempt to resolve as many as possible of the alerts in all categories. Often the minor alerts point to easily fixed oversights, errors and omissions in your CIF or refinement strategy, so attention to these fine details can be worthwhile. In order to resolve some of the more serious problems it may be necessary to carry out additional measurements or structure refinements. However, the purpose of your study may justify the reported deviations and the more serious of these should normally be commented upon in the discussion or experimental section of a paper or in the "special_details" fields of the CIF. checkCIF was carefully designed to identify outliers and unusual parameters, but every test has its limitations and alerts that are not important in a particular case may appear. Conversely, the absence of alerts does not guarantee there are no aspects of the results needing attention. It is up to the individual to critically assess their own results and, if necessary, seek expert advice.

Publication of your CIF in IUCr journals

A basic structural check has been run on your CIF. These basic checks will be run on all CIFs submitted for publication in IUCr journals (*Acta Crystallographica*, *Journal of Applied Crystallography*, *Journal of Synchrotron Radiation*); however, if you intend to submit to *Acta Crystallographica Section C* or *E* or *IUCrData*, you should make sure that full publication checks are run on the final version of your CIF prior to submission.

Publication of your CIF in other journals

Please refer to the *Notes for Authors* of the relevant journal for any special instructions relating to CIF submission.

PLATON version of 03/06/2021; check.def file version of 02/06/2021

Datablock edu6764_sq - ellipsoid plot

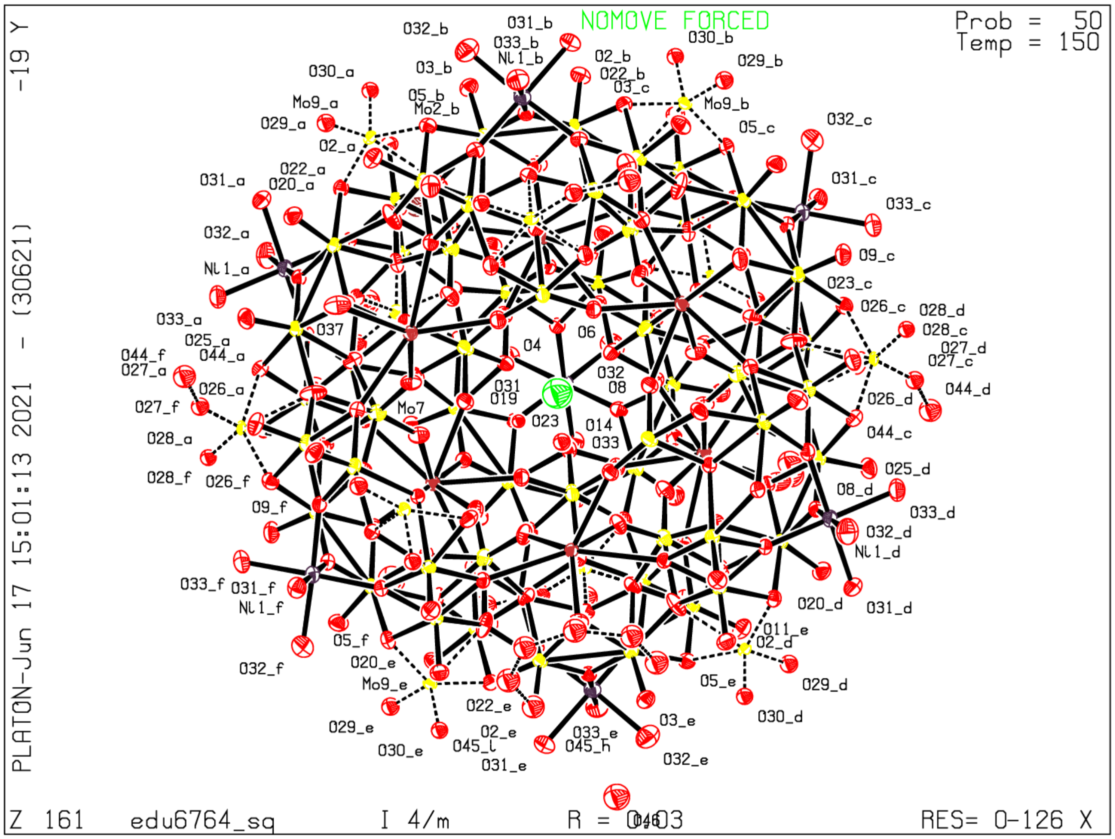

Supplement: Supplementary file 4 — Supporting Information [file ANIE-61-0-s015.pdf]
