# Supplementary material for: Engineering Highly Reduced Molybdenum Polyoxometalates via the Incorporation of d and f Block Metal Ions
Source: Angew Chem Int Ed Engl. 2022 Mar 23;61(21):e202201672. doi: 10.1002/anie.202201672 (PMC9401863; doi:10.1002/anie.202201672)

checkCIF/PLATON report

Structure factors have been supplied for datablock(s) edu6851_sq

THIS REPORT IS FOR GUIDANCE ONLY. IF USED AS PART OF A REVIEW PROCEDURE FOR PUBLICATION, IT SHOULD NOT REPLACE THE EXPERTISE OF AN EXPERIENCED CRYSTALLOGRAPHIC REFEREE.

No syntax errors found. CIF dictionary Interpreting this report

Datablock: edu6851_sq

Bond precision:	Ni- O = 0.0040 A		Wavelength=0.71073
Cell:	a=24.3944(3)	b=24.3944(3)	c=22.8076(5)
	alpha=90	beta=90	gamma=90
Temperature:	150 K		

	Calculated	Reported
Volume	13572.5(4)	13572.5(4)
Space group	I 4/m	I 4/m
Hall group	-I 4	-I 4
Moiety formula	2(Ce6 Mo64 Ni8 O230), Cl, 65.333(O), 1.92(Na) [+ solvent]	?
Sum formula	Ce12 Cl Mo128 Na1.92 Ni16 O525.33 [+ solvent]	Ce6 H196 Cl Mo64 Na9 Ni8 O289
Mr	23385.62	12514.48
Dx,g cm-3	2.861	3.062
Z	1	2
Mu (mm-1)	4.482	4.515
F000	10760.7	11768.0
F000’	10548.09	
h,k,lmax	30,30,28	30,29,28
Nref	6868	6385
Tmin,Tmax	0.850,0.873	0.851,0.925
Tmin’	0.798	

Correction method= # Reported T Limits: Tmin=0.851 Tmax=0.925
AbsCorr = GAUSSIAN

Data completeness= 0.930 Theta(max)= 25.999

R(reflections)= 0.0271(5452) wR2(reflections)= 0.0763(6385)

S = 1.073 Npar= 424

The following ALERTS were generated. Each ALERT has the format
test-name_ALERT_alert-type_alert-level.
Click on the hyperlinks for more details of the test.

Alert level A

```
PLAT029_ALERT_3_A _diffrn_measured_fraction_theta_full value Low .      0.934 Why?
```

Alert level B

```
PLAT911_ALERT_3_B Missing FCF Refl Between Thmin & STh/L=    0.600        410 Report
```

Alert level C

```
PLAT077_ALERT_4_C Unitcell Contains Non-integer Number of Atoms ..     Please Check
PLAT202_ALERT_3_C Isotropic non-H Atoms in Anion/Solvent .........          3 Check
              O36     O37     O42
PLAT910_ALERT_3_C Missing # of FCF Reflection(s) Below Theta(Min).          8 Note
PLAT913_ALERT_3_C Missing # of Very Strong Reflections in FCF ....          4 Note
PLAT934_ALERT_3_C Number of (Iobs-Icalc)/Sigma(W) > 10 Outliers ..          1 Check
PLAT975_ALERT_2_C Check Calcd Resid. Dens.  0.80A   From O38             1.20 eA-3
PLAT975_ALERT_2_C Check Calcd Resid. Dens.  0.65A   From O7              0.72 eA-3
PLAT975_ALERT_2_C Check Calcd Resid. Dens.  0.95A   From O26             0.70 eA-3
PLAT975_ALERT_2_C Check Calcd Resid. Dens.  0.82A   From O48             0.66 eA-3
PLAT976_ALERT_2_C Check Calcd Resid. Dens.  0.49A   From O45            -0.57 eA-3
```

Alert level G

```
FORMU01_ALERT_2_G  There is a discrepancy between the atom counts in the
            _chemical_formula_sum and the formula from the _atom_site* data.
            Atom count from _chemical_formula_sum:H196 Ce6 Cl1 Mo64 Na9 Ni8 O289
            Atom count from the _atom_site data:  Ce6 Cl0.5 Mo64.00460 Na0.96 Ni8
CELLZ01_ALERT_1_G Difference between formula and atom_site contents detected.
CELLZ01_ALERT_1_G ALERT: Large difference may be due to a
            symmetry error - see SYMMG tests
           From the CIF: _cell_formula_units_Z    2
           From the CIF: _chemical_formula_sum  Ce6 H196 Cl Mo64 Na9 Ni8 O289
           TEST: Compare cell contents of formula and atom_site data

           atom    Z*formula  cif sites diff
           Ce        12.00     12.00    0.00
           H        392.00      0.00  392.00
           Cl         2.00      1.00    1.00
           Mo       128.00    128.00    0.00
           Na        18.00      1.92   16.08
           Ni        16.00     16.00    0.00
           O        578.00    525.33   52.67
PLAT041_ALERT_1_G Calc. and Reported SumFormula    Strings  Differ     Please Check
PLAT045_ALERT_1_G Calculated and Reported Z Differ by a Factor ...       0.50 Check
PLAT051_ALERT_1_G Mu(calc) and Mu(CIF) Ratio Differs from 1.0 by .       0.74 %
PLAT083_ALERT_2_G SHELXL Second Parameter in WGHT  Unusually Large      58.65 Why ?
PLAT172_ALERT_4_G The CIF-Embedded .res File Contains DFIX Records          1 Report
PLAT300_ALERT_4_G Atom Site Occupancy of Mo9        Constrained at     0.3333 Check
PLAT300_ALERT_4_G Atom Site Occupancy of Mo10       Constrained at     0.3333 Check
PLAT300_ALERT_4_G Atom Site Occupancy of O32        Constrained at     0.3333 Check
PLAT300_ALERT_4_G Atom Site Occupancy of O33        Constrained at     0.3333 Check
PLAT300_ALERT_4_G Atom Site Occupancy of O34        Constrained at     0.3333 Check
PLAT300_ALERT_4_G Atom Site Occupancy of O35        Constrained at     0.3333 Check
PLAT300_ALERT_4_G Atom Site Occupancy of Cl1        Constrained at        0.5 Check
PLAT300_ALERT_4_G Atom Site Occupancy of O36        Constrained at     0.6667 Check
```

```
PLAT300_ALERT_4_G Atom Site Occupancy of O37        Constrained at     0.6667 Check
PLAT300_ALERT_4_G Atom Site Occupancy of O42        Constrained at       0.55 Check
PLAT300_ALERT_4_G Atom Site Occupancy of O38        Constrained at     0.3333 Check
PLAT300_ALERT_4_G Atom Site Occupancy of O42’       Constrained at        0.4 Check
PLAT300_ALERT_4_G Atom Site Occupancy of O43        Constrained at        0.4 Check
PLAT300_ALERT_4_G Atom Site Occupancy of O43’       Constrained at        0.2 Check
PLAT300_ALERT_4_G Atom Site Occupancy of O44        Constrained at       0.45 Check
PLAT300_ALERT_4_G Atom Site Occupancy of O44’       Constrained at        0.3 Check
PLAT300_ALERT_4_G Atom Site Occupancy of O45        Constrained at     0.3333 Check
PLAT300_ALERT_4_G Atom Site Occupancy of O46        Constrained at     0.3333 Check
PLAT300_ALERT_4_G Atom Site Occupancy of O47        Constrained at        0.2 Check
PLAT300_ALERT_4_G Atom Site Occupancy of O48        Constrained at        0.3 Check
PLAT300_ALERT_4_G Atom Site Occupancy of Na1        Constrained at       0.24 Check
PLAT301_ALERT_3_G Main Residue  Disorder .............(Resd  1  )          5% Note
PLAT302_ALERT_4_G Anion/Solvent/Minor-Residue Disorder (Resd  2  )       100% Note
PLAT302_ALERT_4_G Anion/Solvent/Minor-Residue Disorder (Resd  3  )       100% Note
PLAT302_ALERT_4_G Anion/Solvent/Minor-Residue Disorder (Resd  4  )       100% Note
PLAT302_ALERT_4_G Anion/Solvent/Minor-Residue Disorder (Resd  5  )       100% Note
PLAT302_ALERT_4_G Anion/Solvent/Minor-Residue Disorder (Resd  6  )       100% Note
PLAT302_ALERT_4_G Anion/Solvent/Minor-Residue Disorder (Resd  7  )       100% Note
PLAT302_ALERT_4_G Anion/Solvent/Minor-Residue Disorder (Resd  8  )       100% Note
PLAT302_ALERT_4_G Anion/Solvent/Minor-Residue Disorder (Resd  9  )       100% Note
PLAT302_ALERT_4_G Anion/Solvent/Minor-Residue Disorder (Resd 10  )       100% Note
PLAT302_ALERT_4_G Anion/Solvent/Minor-Residue Disorder (Resd 11  )       100% Note
PLAT302_ALERT_4_G Anion/Solvent/Minor-Residue Disorder (Resd 12  )       100% Note
PLAT302_ALERT_4_G Anion/Solvent/Minor-Residue Disorder (Resd 13  )       100% Note
PLAT302_ALERT_4_G Anion/Solvent/Minor-Residue Disorder (Resd 14  )       100% Note
PLAT302_ALERT_4_G Anion/Solvent/Minor-Residue Disorder (Resd 15  )       100% Note
PLAT302_ALERT_4_G Anion/Solvent/Minor-Residue Disorder (Resd 16  )       100% Note
PLAT304_ALERT_4_G Non-Integer Number of Atoms in ..... (Resd  1  )     269.50 Check
PLAT304_ALERT_4_G Non-Integer Number of Atoms in ..... (Resd  2  )       0.06 Check
PLAT304_ALERT_4_G Non-Integer Number of Atoms in ..... (Resd  3  )       0.33 Check
PLAT304_ALERT_4_G Non-Integer Number of Atoms in ..... (Resd  4  )       0.67 Check
PLAT304_ALERT_4_G Non-Integer Number of Atoms in ..... (Resd  5  )       0.55 Check
PLAT304_ALERT_4_G Non-Integer Number of Atoms in ..... (Resd  6  )       0.33 Check
PLAT304_ALERT_4_G Non-Integer Number of Atoms in ..... (Resd  7  )       0.40 Check
PLAT304_ALERT_4_G Non-Integer Number of Atoms in ..... (Resd  8  )       0.20 Check
PLAT304_ALERT_4_G Non-Integer Number of Atoms in ..... (Resd  9  )       0.10 Check
PLAT304_ALERT_4_G Non-Integer Number of Atoms in ..... (Resd 10  )       0.45 Check
PLAT304_ALERT_4_G Non-Integer Number of Atoms in ..... (Resd 11  )       0.30 Check
PLAT304_ALERT_4_G Non-Integer Number of Atoms in ..... (Resd 12  )       0.17 Check
PLAT304_ALERT_4_G Non-Integer Number of Atoms in ..... (Resd 13  )       0.08 Check
PLAT304_ALERT_4_G Non-Integer Number of Atoms in ..... (Resd 14  )       0.20 Check
PLAT304_ALERT_4_G Non-Integer Number of Atoms in ..... (Resd 15  )       0.30 Check
PLAT304_ALERT_4_G Non-Integer Number of Atoms in ..... (Resd 16  )       0.12 Check
PLAT311_ALERT_2_G Isolated Disordered Oxygen Atom (No H’s ?) .....        O36 Check
PLAT311_ALERT_2_G Isolated Disordered Oxygen Atom (No H’s ?) .....        O37 Check
PLAT311_ALERT_2_G Isolated Disordered Oxygen Atom (No H’s ?) .....        O42 Check
PLAT311_ALERT_2_G Isolated Disordered Oxygen Atom (No H’s ?) .....        O38 Check
PLAT311_ALERT_2_G Isolated Disordered Oxygen Atom (No H’s ?) .....       O42’ Check
PLAT311_ALERT_2_G Isolated Disordered Oxygen Atom (No H’s ?) .....        O43 Check
PLAT311_ALERT_2_G Isolated Disordered Oxygen Atom (No H’s ?) .....       O43’ Check
PLAT311_ALERT_2_G Isolated Disordered Oxygen Atom (No H’s ?) .....        O44 Check
PLAT311_ALERT_2_G Isolated Disordered Oxygen Atom (No H’s ?) .....       O44’ Check
PLAT311_ALERT_2_G Isolated Disordered Oxygen Atom (No H’s ?) .....        O45 Check
PLAT311_ALERT_2_G Isolated Disordered Oxygen Atom (No H’s ?) .....        O46 Check
PLAT311_ALERT_2_G Isolated Disordered Oxygen Atom (No H’s ?) .....        O47 Check
PLAT311_ALERT_2_G Isolated Disordered Oxygen Atom (No H’s ?) .....        O48 Check
PLAT606_ALERT_4_G Solvent Accessible VOID(S) in Structure ........          ! Info
PLAT790_ALERT_4_G Centre of Gravity not Within Unit Cell: Resd.  #          4 Note
              O
PLAT790_ALERT_4_G Centre of Gravity not Within Unit Cell: Resd.  #          6 Note
```

```
              O
PLAT794_ALERT_5_G Tentative Bond Valency for Ce1       (III)     .       3.06 Info
PLAT794_ALERT_5_G Tentative Bond Valency for Ce2       (III)     .       3.05 Info
PLAT794_ALERT_5_G Tentative Bond Valency for Mo7       (VI)      .       6.07 Info
PLAT794_ALERT_5_G Tentative Bond Valency for Mo8       (VI)      .       6.09 Info
PLAT794_ALERT_5_G Tentative Bond Valency for Ni1       (II)      .       1.99 Info
PLAT869_ALERT_4_G ALERTS Related to the Use of SQUEEZE Suppressed           ! Info
PLAT912_ALERT_4_G Missing # of FCF Reflections Above STh/L=  0.600         67 Note
PLAT933_ALERT_2_G Number of OMIT Records in Embedded .res File ...          6 Note
PLAT941_ALERT_3_G Average HKL Measurement Multiplicity ...........        1.8 Low
PLAT965_ALERT_2_G The SHELXL WEIGHT Optimisation has not Converged     Please Check
```

```
  1 ALERT level A = Most likely a serious problem - resolve or explain
  1 ALERT level B = A potentially serious problem, consider carefully
 10 ALERT level C = Check. Ensure it is not caused by an omission or oversight
 87 ALERT level G = General information/check it is not something unexpected

  5 ALERT type 1 CIF construction/syntax error, inconsistent or missing data
 22 ALERT type 2 Indicator that the structure model may be wrong or deficient
  8 ALERT type 3 Indicator that the structure quality may be low
 59 ALERT type 4 Improvement, methodology, query or suggestion
  5 ALERT type 5 Informative message, check
```

It is advisable to attempt to resolve as many as possible of the alerts in all categories. Often the minor alerts point to easily fixed oversights, errors and omissions in your CIF or refinement strategy, so attention to these fine details can be worthwhile. In order to resolve some of the more serious problems it may be necessary to carry out additional measurements or structure refinements. However, the purpose of your study may justify the reported deviations and the more serious of these should normally be commented upon in the discussion or experimental section of a paper or in the "special_details" fields of the CIF. checkCIF was carefully designed to identify outliers and unusual parameters, but every test has its limitations and alerts that are not important in a particular case may appear. Conversely, the absence of alerts does not guarantee there are no aspects of the results needing attention. It is up to the individual to critically assess their own results and, if necessary, seek expert advice.

Publication of your CIF in IUCr journals

A basic structural check has been run on your CIF. These basic checks will be run on all CIFs submitted for publication in IUCr journals (*Acta Crystallographica*, *Journal of Applied Crystallography*, *Journal of Synchrotron Radiation*); however, if you intend to submit to *Acta Crystallographica Section C* or *E* or *IUCrData*, you should make sure that full publication checks are run on the final version of your CIF prior to submission.

Publication of your CIF in other journals

Please refer to the *Notes for Authors* of the relevant journal for any special instructions relating to CIF submission.

PLATON version of 03/06/2021; check.def file version of 02/06/2021

Datablock edu6851_sq - ellipsoid plot

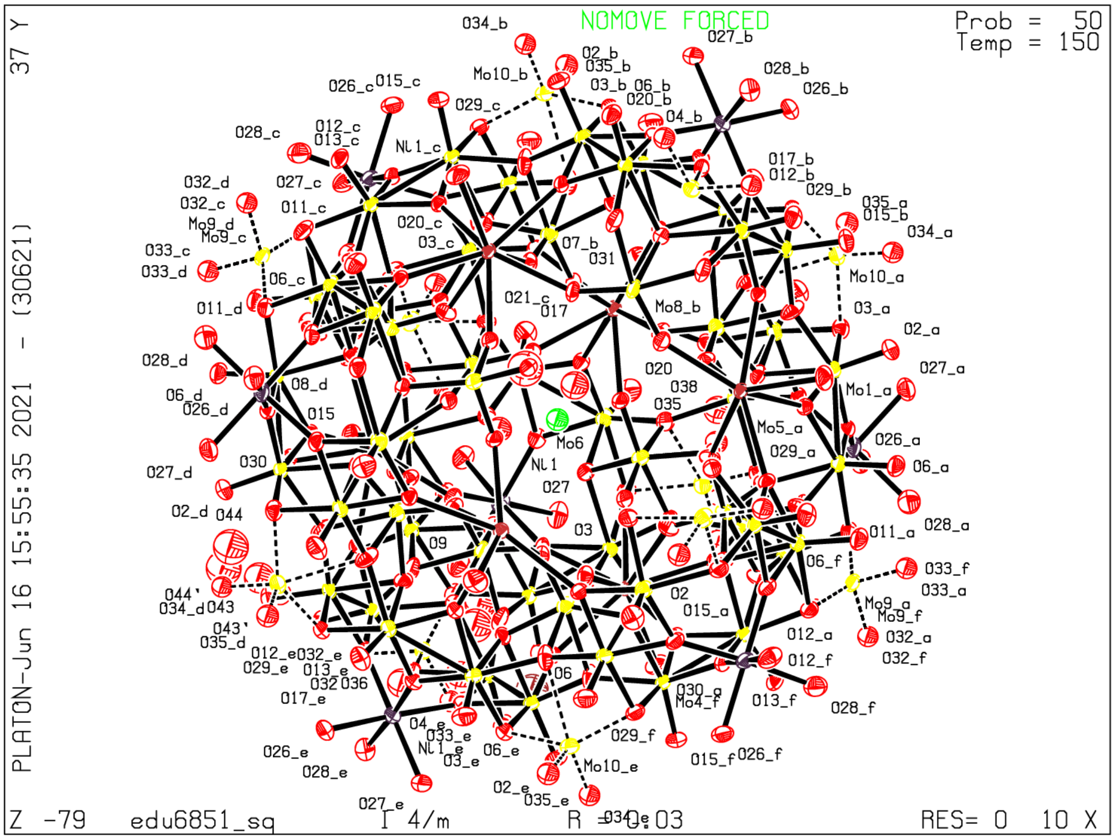

Supplement: Supplementary file 6 — Supporting Information [file ANIE-61-0-s013.pdf]
