# Supplementary material for: Engineering Highly Reduced Molybdenum Polyoxometalates via the Incorporation of d and f Block Metal Ions
Source: Angew Chem Int Ed Engl. 2022 Mar 23;61(21):e202201672. doi: 10.1002/anie.202201672 (PMC9401863; doi:10.1002/anie.202201672)

checkCIF/PLATON report

Structure factors have been supplied for datablock(s) edu862r_sq

THIS REPORT IS FOR GUIDANCE ONLY. IF USED AS PART OF A REVIEW PROCEDURE FOR PUBLICATION, IT SHOULD NOT REPLACE THE EXPERTISE OF AN EXPERIENCED CRYSTALLOGRAPHIC REFEREE.

No syntax errors found. CIF dictionary Interpreting this report

Datablock: edu862r_sq

Bond precision:	Pr- O = 0.0044 A		Wavelength=0.71073
Cell:	a=25.5441(2)	b=25.5441(2)	c=18.8128(2)
	alpha=90	beta=90	gamma=90
Temperature:	150 K		

	Calculated	Reported
Volume	12275.4(2)	12275.4(2)
Space group	I 4/m	I 4/m
Hall group	-I 4	-I 4
Moiety formula	Mo128 Ni16 O460.80 Pr12, Cl, 44.72(O) [+ solvent]	?
Sum formula	Cl Mo128 Ni16 O505.52 Pr12 [+ solvent]	Cl H146 Mo64 Na9 Ni8 O264 Pr6
Mr	23034.05	12068.82
Dx,g cm-3	3.116	3.265
Z	1	2
Mu (mm-1)	5.027	5.057
F000	10593.2	11280.0
F000’	10380.68	
h,k,lmax	31,31,23	31,31,23
Nref	6229	6221
Tmin,Tmax	0.886,0.951	0.745,1.000
Tmin’	0.603	

Correction method= # Reported T Limits: Tmin=0.745 Tmax=1.000
AbsCorr = GAUSSIAN

Data completeness= 0.999 Theta(max)= 25.992

R(reflections)= 0.0276(5795) wR2(reflections)= 0.0784(6221)

S = 1.049 Npar= 421

The following ALERTS were generated. Each ALERT has the format
test-name_ALERT_alert-type_alert-level.
Click on the hyperlinks for more details of the test.

Alert level B

```
PLAT975_ALERT_2_B Check Calcd Resid. Dens.  0.70A   From O38            1.91 eA-3
PLAT975_ALERT_2_B Check Calcd Resid. Dens.  0.62A   From O38            1.58 eA-3
```

Alert level C

```
PLAT094_ALERT_2_C Ratio of Maximum / Minimum Residual Density ....       2.13 Report
PLAT220_ALERT_2_C NonSolvent   Resd 1  O   Ueq(max)/Ueq(min) Range        3.3 Ratio
PLAT910_ALERT_3_C Missing # of FCF Reflection(s) Below Theta(Min).          7 Note
PLAT911_ALERT_3_C Missing FCF Refl Between Thmin & STh/L=    0.600          3 Report
PLAT918_ALERT_3_C Reflection(s) with I(obs) much Smaller I(calc) .          2 Check
PLAT975_ALERT_2_C Check Calcd Resid. Dens.  0.90A   From O40            0.93 eA-3
```

Alert level G

```
FORMU01_ALERT_2_G  There is a discrepancy between the atom counts in the
            _chemical_formula_sum and the formula from the _atom_site* data.
            Atom count from _chemical_formula_sum:H146 Cl1 Mo64 Na9 Ni8 O264 Pr6
            Atom count from the _atom_site data:  Cl0.5 Mo64 Ni8 O252.7600 Pr6
CELLZ01_ALERT_1_G Difference between formula and atom_site contents detected.
CELLZ01_ALERT_1_G ALERT: Large difference may be due to a
            symmetry error - see SYMMG tests
           From the CIF: _cell_formula_units_Z    2
           From the CIF: _chemical_formula_sum  Cl H146 Mo64 Na9 Ni8 O264 Pr6
           TEST: Compare cell contents of formula and atom_site data

           atom    Z*formula  cif sites diff
           Cl         2.00      1.00    1.00
           H        292.00      0.00  292.00
           Mo       128.00    128.00    0.00
           Na        18.00      0.00   18.00
           Ni        16.00     16.00    0.00
           O        528.00    505.52   22.48
           Pr        12.00     12.00    0.00
PLAT002_ALERT_2_G Number of Distance or Angle Restraints on AtSite          3 Note
PLAT041_ALERT_1_G Calc. and Reported SumFormula    Strings  Differ     Please Check
PLAT045_ALERT_1_G Calculated and Reported Z Differ by a Factor ...       0.50 Check
PLAT051_ALERT_1_G Mu(calc) and Mu(CIF) Ratio Differs from 1.0 by .       0.60 %
PLAT083_ALERT_2_G SHELXL Second Parameter in WGHT  Unusually Large     169.42 Why ?
PLAT172_ALERT_4_G The CIF-Embedded .res File Contains DFIX Records          1 Report
PLAT300_ALERT_4_G Atom Site Occupancy of Mo9       Constrained at        0.5 Check
PLAT300_ALERT_4_G Atom Site Occupancy of Mo10      Constrained at       0.25 Check
PLAT300_ALERT_4_G Atom Site Occupancy of Ni1       Constrained at       0.92 Check
PLAT300_ALERT_4_G Atom Site Occupancy of Ni1'      Constrained at       0.08 Check
PLAT300_ALERT_4_G Atom Site Occupancy of O33       Constrained at        0.5 Check
PLAT300_ALERT_4_G Atom Site Occupancy of O35       Constrained at        0.5 Check
PLAT300_ALERT_4_G Atom Site Occupancy of O32       Constrained at       0.25 Check
PLAT300_ALERT_4_G Atom Site Occupancy of O36       Constrained at        0.3 Check
PLAT300_ALERT_4_G Atom Site Occupancy of Cl1       Constrained at        0.5 Check
PLAT300_ALERT_4_G Atom Site Occupancy of O32'      Constrained at        0.5 Check
PLAT300_ALERT_4_G Atom Site Occupancy of O34       Constrained at        0.5 Check
PLAT300_ALERT_4_G Atom Site Occupancy of O37       Constrained at        0.5 Check
PLAT300_ALERT_4_G Atom Site Occupancy of O38       Constrained at        0.5 Check
PLAT300_ALERT_4_G Atom Site Occupancy of O39       Constrained at        0.5 Check
PLAT300_ALERT_4_G Atom Site Occupancy of O42       Constrained at        0.5 Check
PLAT300_ALERT_4_G Atom Site Occupancy of O43       Constrained at        0.5 Check
```

```
PLAT300_ALERT_4_G Atom Site Occupancy of O40        Constrained at        0.4 Check
PLAT300_ALERT_4_G Atom Site Occupancy of O41        Constrained at        0.4 Check
PLAT300_ALERT_4_G Atom Site Occupancy of O41'       Constrained at        0.4 Check
PLAT300_ALERT_4_G Atom Site Occupancy of O44        Constrained at       0.24 Check
PLAT300_ALERT_4_G Atom Site Occupancy of O45        Constrained at        0.2 Check
PLAT300_ALERT_4_G Atom Site Occupancy of O46        Constrained at        0.1 Check
PLAT301_ALERT_3_G Main Residue  Disorder .............(Resd  1  )          8% Note
PLAT302_ALERT_4_G Anion/Solvent/Minor-Residue Disorder (Resd  2  )       100% Note
PLAT302_ALERT_4_G Anion/Solvent/Minor-Residue Disorder (Resd  3  )       100% Note
PLAT302_ALERT_4_G Anion/Solvent/Minor-Residue Disorder (Resd  4  )       100% Note
PLAT302_ALERT_4_G Anion/Solvent/Minor-Residue Disorder (Resd  5  )       100% Note
PLAT302_ALERT_4_G Anion/Solvent/Minor-Residue Disorder (Resd  6  )       100% Note
PLAT302_ALERT_4_G Anion/Solvent/Minor-Residue Disorder (Resd  7  )       100% Note
PLAT302_ALERT_4_G Anion/Solvent/Minor-Residue Disorder (Resd  8  )       100% Note
PLAT302_ALERT_4_G Anion/Solvent/Minor-Residue Disorder (Resd  9  )       100% Note
PLAT302_ALERT_4_G Anion/Solvent/Minor-Residue Disorder (Resd 10  )       100% Note
PLAT302_ALERT_4_G Anion/Solvent/Minor-Residue Disorder (Resd 11  )       100% Note
PLAT302_ALERT_4_G Anion/Solvent/Minor-Residue Disorder (Resd 12  )       100% Note
PLAT302_ALERT_4_G Anion/Solvent/Minor-Residue Disorder (Resd 13  )       100% Note
PLAT302_ALERT_4_G Anion/Solvent/Minor-Residue Disorder (Resd 14  )       100% Note
PLAT302_ALERT_4_G Anion/Solvent/Minor-Residue Disorder (Resd 15  )       100% Note
PLAT304_ALERT_4_G Non-Integer Number of Atoms in ..... (Resd  1  )     269.85 Check
PLAT304_ALERT_4_G Non-Integer Number of Atoms in ..... (Resd  2  )       0.06 Check
PLAT304_ALERT_4_G Non-Integer Number of Atoms in ..... (Resd  3  )       0.50 Check
PLAT304_ALERT_4_G Non-Integer Number of Atoms in ..... (Resd  4  )       0.25 Check
PLAT304_ALERT_4_G Non-Integer Number of Atoms in ..... (Resd  5  )       0.25 Check
PLAT304_ALERT_4_G Non-Integer Number of Atoms in ..... (Resd  6  )       0.25 Check
PLAT304_ALERT_4_G Non-Integer Number of Atoms in ..... (Resd  7  )       0.25 Check
PLAT304_ALERT_4_G Non-Integer Number of Atoms in ..... (Resd  8  )       0.25 Check
PLAT304_ALERT_4_G Non-Integer Number of Atoms in ..... (Resd  9  )       0.13 Check
PLAT304_ALERT_4_G Non-Integer Number of Atoms in ..... (Resd 10  )       0.20 Check
PLAT304_ALERT_4_G Non-Integer Number of Atoms in ..... (Resd 11  )       0.20 Check
PLAT304_ALERT_4_G Non-Integer Number of Atoms in ..... (Resd 12  )       0.20 Check
PLAT304_ALERT_4_G Non-Integer Number of Atoms in ..... (Resd 13  )       0.12 Check
PLAT304_ALERT_4_G Non-Integer Number of Atoms in ..... (Resd 14  )       0.10 Check
PLAT304_ALERT_4_G Non-Integer Number of Atoms in ..... (Resd 15  )       0.10 Check
PLAT311_ALERT_2_G Isolated Disordered Oxygen Atom (No H's ?) .....       O32' Check
PLAT311_ALERT_2_G Isolated Disordered Oxygen Atom (No H's ?) .....        O34 Check
PLAT311_ALERT_2_G Isolated Disordered Oxygen Atom (No H's ?) .....        O37 Check
PLAT311_ALERT_2_G Isolated Disordered Oxygen Atom (No H's ?) .....        O38 Check
PLAT311_ALERT_2_G Isolated Disordered Oxygen Atom (No H's ?) .....        O39 Check
PLAT311_ALERT_2_G Isolated Disordered Oxygen Atom (No H's ?) .....        O42 Check
PLAT311_ALERT_2_G Isolated Disordered Oxygen Atom (No H's ?) .....        O43 Check
PLAT311_ALERT_2_G Isolated Disordered Oxygen Atom (No H's ?) .....        O40 Check
PLAT311_ALERT_2_G Isolated Disordered Oxygen Atom (No H's ?) .....        O41 Check
PLAT311_ALERT_2_G Isolated Disordered Oxygen Atom (No H's ?) .....       O41' Check
PLAT311_ALERT_2_G Isolated Disordered Oxygen Atom (No H's ?) .....        O44 Check
PLAT311_ALERT_2_G Isolated Disordered Oxygen Atom (No H's ?) .....        O45 Check
PLAT311_ALERT_2_G Isolated Disordered Oxygen Atom (No H's ?) .....        O46 Check
PLAT606_ALERT_4_G Solvent Accessible VOID(S) in Structure ........          ! Info
PLAT790_ALERT_4_G Centre of Gravity not Within Unit Cell: Resd.  #          3 Note
              O
PLAT794_ALERT_5_G Tentative Bond Valency for Pr1       (III)     .       3.02 Info
PLAT794_ALERT_5_G Tentative Bond Valency for Pr2       (III)     .       3.17 Info
PLAT794_ALERT_5_G Tentative Bond Valency for Mo7       (VI)      .       6.03 Info
PLAT860_ALERT_3_G Number of Least-Squares Restraints ............           2 Note
PLAT869_ALERT_4_G ALERTS Related to the Use of SQUEEZE Suppressed           ! Info
PLAT883_ALERT_1_G No Info/Value for _atom_sites_solution_primary .     Please Do !
PLAT933_ALERT_2_G Number of OMIT Records in Embedded .res File ...          4 Note
PLAT965_ALERT_2_G The SHELXL WEIGHT Optimisation has not Converged     Please Check
```

```
 0 ALERT level A = Most likely a serious problem - resolve or explain
 2 ALERT level B = A potentially serious problem, consider carefully
 6 ALERT level C = Check. Ensure it is not caused by an omission or oversight
84 ALERT level G = General information/check it is not something unexpected

 6 ALERT type 1 CIF construction/syntax error, inconsistent or missing data
23 ALERT type 2 Indicator that the structure model may be wrong or deficient
 5 ALERT type 3 Indicator that the structure quality may be low
55 ALERT type 4 Improvement, methodology, query or suggestion
 3 ALERT type 5 Informative message, check
```

It is advisable to attempt to resolve as many as possible of the alerts in all categories. Often the minor alerts point to easily fixed oversights, errors and omissions in your CIF or refinement strategy, so attention to these fine details can be worthwhile. In order to resolve some of the more serious problems it may be necessary to carry out additional measurements or structure refinements. However, the purpose of your study may justify the reported deviations and the more serious of these should normally be commented upon in the discussion or experimental section of a paper or in the "special_details" fields of the CIF. checkCIF was carefully designed to identify outliers and unusual parameters, but every test has its limitations and alerts that are not important in a particular case may appear. Conversely, the absence of alerts does not guarantee there are no aspects of the results needing attention. It is up to the individual to critically assess their own results and, if necessary, seek expert advice.

Publication of your CIF in IUCr journals

A basic structural check has been run on your CIF. These basic checks will be run on all CIFs submitted for publication in IUCr journals (*Acta Crystallographica*, *Journal of Applied Crystallography*, *Journal of Synchrotron Radiation*); however, if you intend to submit to *Acta Crystallographica Section C* or *E* or *IUCrData*, you should make sure that full publication checks are run on the final version of your CIF prior to submission.

Publication of your CIF in other journals

Please refer to the *Notes for Authors* of the relevant journal for any special instructions relating to CIF submission.

PLATON version of 03/06/2021; check.def file version of 02/06/2021

Datablock edu862r_sq - ellipsoid plot

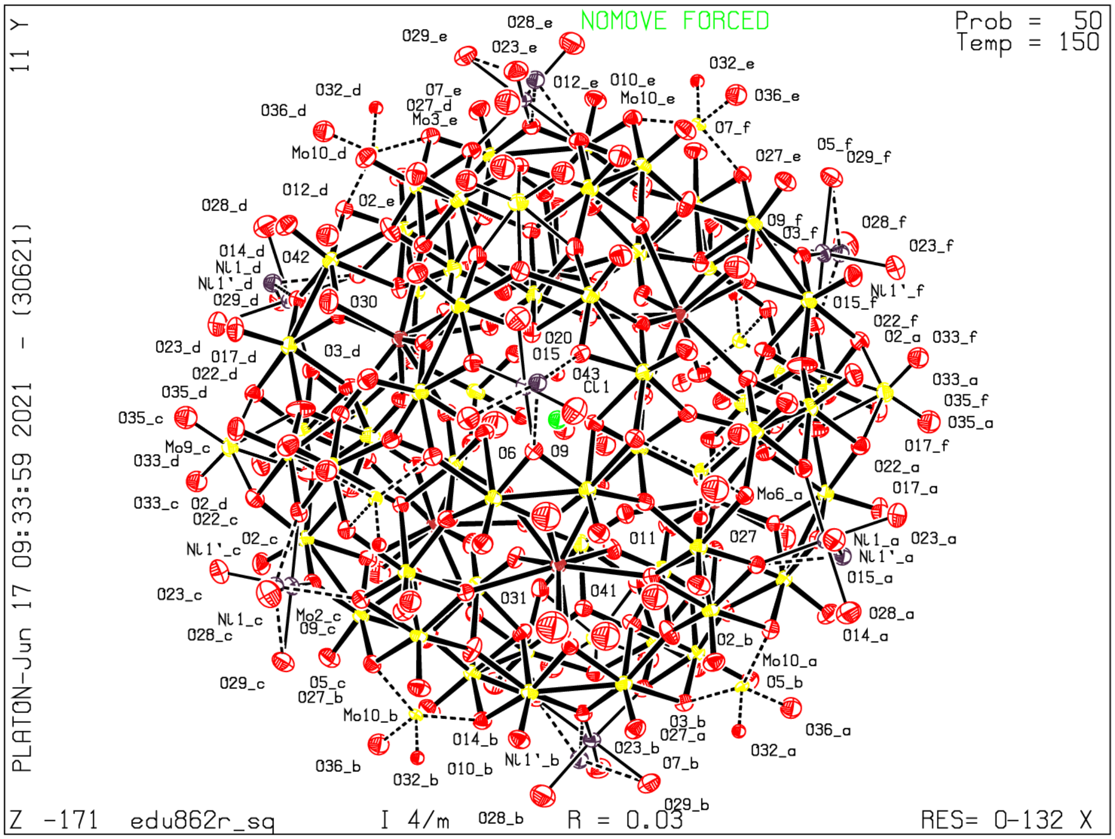

Supplement: Supplementary file 8 — Supporting Information [file ANIE-61-0-s006.pdf]
