# Supplementary material for: Engineering Highly Reduced Molybdenum Polyoxometalates via the Incorporation of d and f Block Metal Ions
Source: Angew Chem Int Ed Engl. 2022 Mar 23;61(21):e202201672. doi: 10.1002/anie.202201672 (PMC9401863; doi:10.1002/anie.202201672)

checkCIF/PLATON report

Structure factors have been supplied for datablock(s) edu7571_sq

THIS REPORT IS FOR GUIDANCE ONLY. IF USED AS PART OF A REVIEW PROCEDURE FOR PUBLICATION, IT SHOULD NOT REPLACE THE EXPERTISE OF AN EXPERIENCED CRYSTALLOGRAPHIC REFEREE.

No syntax errors found. CIF dictionary Interpreting this report

Datablock: edu7571_sq

```
Bond precision:  Ce- O = 0.0115 A        Wavelength=0.71073

Cell:            a=60.7906(7)     b=26.8065(2)       c=50.4181(6)
                 alpha=90         beta=91.678(2)     gamma=90
Temperature:     150 K
```

	Calculated	Reported
Volume	82125.3(15)	82125.2(15)
Space group	C 2/c	C 2/c
Hall group	-C 2yc	-C 2yc
Moiety formula	Ce36 Mo560 O881.60, 8(Ce O8.20), 8(Ni0.50 O), 8(Cl0.50), 139(O0	?
Sum formula	Ce44 Cl4 Mo560 Na8 Ni4 O092.80 [+ solvent]	Ce5.50 H530 Cl0.50 Mo70 Na3 Ni0.50 O465
Mr	93936.99	15576.72
Dx,g cm-3	1.899	2.520
Z	1	8
Mu (mm-1)	2.750	2.820
F000	43082.4	60516.0
F000’	42129.71	
h,k,lmax	74,33,62	74,32,62
Nref	80679	80376
Tmin,Tmax	0.809,0.903	0.878,0.983
Tmin’	0.809	

```
Correction method= # Reported T Limits: Tmin=0.878 Tmax=0.983
AbsCorr = GAUSSIAN

Data completeness= 0.996          Theta(max)= 26.000

R(reflections)= 0.0728( 43816)    wR2(reflections)= 0.1790( 80376)

S = 0.987                  Npar= 3067
```

The following ALERTS were generated. Each ALERT has the format
test-name_ALERT_alert-type_alert-level.
Click on the hyperlinks for more details of the test.

Alert level A

```
RINTA01_ALERT_3_A  The value of Rint is greater than 0.25
            Rint given   0.342
```

Author Response: poor crystal with weak diffraction

```
PLAT020_ALERT_3_A The Value of Rint is Greater Than 0.12 .........      0.342 Report
```

Author Response: poor crystal with weak diffraction

```
PLAT214_ALERT_2_A Atom Na1       (Anion/Solvent) ADP max/min Ratio        6.9 prolat
```

Author Response: possible disorder of position splitting

```
PLAT910_ALERT_3_A Missing # of FCF Reflection(s) Below Theta(Min).         58 Note
```

Author Response: poor crystal with weak diffraction caused data missing

```
PLAT975_ALERT_2_A Check Calcd Resid. Dens.  1.09A   From O247            2.43 eA-3
```

Author Response: disordered solvent area unmodelled

Alert level B

```
PLAT213_ALERT_2_B Atom O109            has ADP max/min Ratio .....        4.6 prolat
PLAT306_ALERT_2_B Isolated Oxygen Atom (H-atoms Missing ?) .......       O257 Check
PLAT306_ALERT_2_B Isolated Oxygen Atom (H-atoms Missing ?) .......       O258 Check
PLAT306_ALERT_2_B Isolated Oxygen Atom (H-atoms Missing ?) .......       O259 Check
PLAT306_ALERT_2_B Isolated Oxygen Atom (H-atoms Missing ?) .......       O260 Check
PLAT306_ALERT_2_B Isolated Oxygen Atom (H-atoms Missing ?) .......       O261 Check
PLAT306_ALERT_2_B Isolated Oxygen Atom (H-atoms Missing ?) .......       O262 Check
PLAT306_ALERT_2_B Isolated Oxygen Atom (H-atoms Missing ?) .......       O263 Check
PLAT306_ALERT_2_B Isolated Oxygen Atom (H-atoms Missing ?) .......       O264 Check
PLAT306_ALERT_2_B Isolated Oxygen Atom (H-atoms Missing ?) .......       O265 Check
PLAT306_ALERT_2_B Isolated Oxygen Atom (H-atoms Missing ?) .......       O266 Check
PLAT306_ALERT_2_B Isolated Oxygen Atom (H-atoms Missing ?) .......       O267 Check
PLAT306_ALERT_2_B Isolated Oxygen Atom (H-atoms Missing ?) .......       O268 Check
PLAT306_ALERT_2_B Isolated Oxygen Atom (H-atoms Missing ?) .......       O269 Check
PLAT306_ALERT_2_B Isolated Oxygen Atom (H-atoms Missing ?) .......       O270 Check
PLAT975_ALERT_2_B Check Calcd Resid. Dens.  0.97A   From O247            1.79 eA-3
```

Author Response: disordered solvent area unmodelled

Alert level C

```
PLAT202_ALERT_3_C Isotropic non-H Atoms in Anion/Solvent .........          3 Check
              O246    O251    O255
PLAT213_ALERT_2_C Atom O7               has ADP max/min Ratio .....        3.1 prolat
PLAT213_ALERT_2_C Atom O18              has ADP max/min Ratio .....        3.4 prolat
PLAT213_ALERT_2_C Atom O27              has ADP max/min Ratio .....        3.6 oblate
PLAT213_ALERT_2_C Atom O40              has ADP max/min Ratio .....        3.5 prolat
PLAT213_ALERT_2_C Atom O46              has ADP max/min Ratio .....        3.2 oblate
PLAT213_ALERT_2_C Atom O53              has ADP max/min Ratio .....        3.3 oblate
PLAT213_ALERT_2_C Atom O104             has ADP max/min Ratio .....        3.1 prolat
PLAT213_ALERT_2_C Atom O117             has ADP max/min Ratio .....        3.7 oblate
PLAT213_ALERT_2_C Atom O162             has ADP max/min Ratio .....        3.3 prolat
PLAT213_ALERT_2_C Atom O179             has ADP max/min Ratio .....        3.1 prolat
PLAT213_ALERT_2_C Atom O227             has ADP max/min Ratio .....        3.1 prolat
PLAT213_ALERT_2_C Atom O232             has ADP max/min Ratio .....        3.9 prolat
PLAT214_ALERT_2_C Atom O244       (Anion/Solvent) ADP max/min Ratio        4.1 prolat
```

Author Response: possible disorder of position splitting

```
PLAT220_ALERT_2_C NonSolvent   Resd 1  O   Ueq(max)/Ueq(min) Range        5.1 Ratio
PLAT221_ALERT_2_C Solv./Anion  Resd 2  O   Ueq(max)/Ueq(min) Range        4.6 Ratio
PLAT242_ALERT_2_C Low    'MainMol' Ueq as Compared to Neighbors of        Ce2 Check
PLAT242_ALERT_2_C Low    'MainMol' Ueq as Compared to Neighbors of        Ce3 Check
PLAT242_ALERT_2_C Low    'MainMol' Ueq as Compared to Neighbors of        Ce4 Check
PLAT244_ALERT_4_C Low    'Solvent' Ueq as Compared to Neighbors of        Ce5 Check
PLAT260_ALERT_2_C Large Average Ueq of Residue Including      O269      0.103 Check
PLAT905_ALERT_3_C Negative K value in the Analysis of Variance ...     -1.125 Report
PLAT911_ALERT_3_C Missing FCF Refl Between Thmin & STh/L=    0.600        130 Report
PLAT975_ALERT_2_C Check Calcd Resid. Dens.  0.77A   From O275          1.44 eA-3
```

Author Response: disordered solvent area unmodelled

```
PLAT975_ALERT_2_C Check Calcd Resid. Dens.  0.67A   From O275          1.44 eA-3
```

Author Response: disordered solvent area unmodelled

```
PLAT975_ALERT_2_C Check Calcd Resid. Dens.  0.80A   From O257          1.42 eA-3
```

Author Response: disordered solvent area unmodelled

```
PLAT975_ALERT_2_C Check Calcd Resid. Dens.  0.68A   From O238          1.35 eA-3
```

Author Response: disordered solvent area unmodelled

```
PLAT975_ALERT_2_C Check Calcd Resid. Dens.  0.89A   From O238          1.33 eA-3
```

Author Response: disordered solvent area unmodelled

```
PLAT975_ALERT_2_C Check Calcd Resid. Dens.  0.64A   From O266           1.29 eA-3
```

Author Response: disordered solvent area unmodelled

```
PLAT976_ALERT_2_C Check Calcd Resid. Dens.  0.72A   From O251          -1.08 eA-3
```

Alert level G

```
FORMU01_ALERT_2_G  There is a discrepancy between the atom counts in the
            _chemical_formula_sum and the formula from the _atom_site* data.
            Atom count from _chemical_formula_sum:H530 Ce5.5 Cl0.5 Mo70 Na3 Ni0.5
            Atom count from the _atom_site data:  Ce5.5 Cl0.5 Mo70 Na1 Ni0.5 O261.
CELLZ01_ALERT_1_G Difference between formula and atom_site contents detected.
CELLZ01_ALERT_1_G ALERT: Large difference may be due to a
            symmetry error - see SYMMG tests
           From the CIF: _cell_formula_units_Z    8
           From the CIF: _chemical_formula_sum  Ce5.50 H530 Cl0.50 Mo70 Na3 Ni0.50
           TEST: Compare cell contents of formula and atom_site data
```

atom	Z*formula	cif sites	diff
Ce	44.00	44.00	0.00
H	4240.00	0.00	4240.00
Cl	4.00	4.00	0.00
Mo	560.00	560.00	0.00
Na	24.00	8.00	16.00
Ni	4.00	4.00	0.00
O	3720.00	2092.80	1627.20

```
PLAT002_ALERT_2_G Number of Distance or Angle Restraints on AtSite          4 Note
PLAT003_ALERT_2_G Number of Uiso or Uij Restrained non-H Atoms ...         15 Report
PLAT004_ALERT_5_G Polymeric Structure Found with Maximum Dimension          1 Info
PLAT041_ALERT_1_G Calc. and Reported SumFormula    Strings  Differ     Please Check
PLAT045_ALERT_1_G Calculated and Reported Z Differ by a Factor ...       0.13 Check
PLAT051_ALERT_1_G Mu(calc) and Mu(CIF) Ratio Differs from 1.0 by .       2.49 %
PLAT142_ALERT_4_G s.u. on b - Axis Small or Missing .............    0.00020 Ang.
PLAT172_ALERT_4_G The CIF-Embedded .res File Contains DFIX Records          2 Report
PLAT186_ALERT_4_G The CIF-Embedded .res File Contains ISOR Records          2 Report
PLAT300_ALERT_4_G Atom Site Occupancy of Ce6       Constrained at        0.25 Check
PLAT300_ALERT_4_G Atom Site Occupancy of Ce6'      Constrained at         0.2 Check
PLAT300_ALERT_4_G Atom Site Occupancy of Ce6"      Constrained at        0.05 Check
PLAT300_ALERT_4_G Atom Site Occupancy of O238      Constrained at         0.8 Check
PLAT300_ALERT_4_G Atom Site Occupancy of O272      Constrained at         0.7 Check
PLAT300_ALERT_4_G Atom Site Occupancy of O273      Constrained at         0.7 Check
PLAT300_ALERT_4_G Atom Site Occupancy of O275      Constrained at         0.5 Check
PLAT300_ALERT_4_G Atom Site Occupancy of O276      Constrained at         0.5 Check
PLAT300_ALERT_4_G Atom Site Occupancy of O246      Constrained at         0.7 Check
PLAT300_ALERT_4_G Atom Site Occupancy of O247      Constrained at         0.5 Check
PLAT300_ALERT_4_G Atom Site Occupancy of Ni1       Constrained at         0.5 Check
PLAT300_ALERT_4_G Atom Site Occupancy of O254      Constrained at         0.5 Check
PLAT300_ALERT_4_G Atom Site Occupancy of O256      Constrained at         0.5 Check
PLAT300_ALERT_4_G Atom Site Occupancy of Cl1       Constrained at         0.5 Check
PLAT300_ALERT_4_G Atom Site Occupancy of O251      Constrained at         0.8 Check
PLAT300_ALERT_4_G Atom Site Occupancy of O255      Constrained at         0.8 Check
PLAT300_ALERT_4_G Atom Site Occupancy of O252      Constrained at         0.4 Check
PLAT300_ALERT_4_G Atom Site Occupancy of O253      Constrained at         0.4 Check
PLAT300_ALERT_4_G Atom Site Occupancy of O274      Constrained at        0.25 Check
PLAT300_ALERT_4_G Atom Site Occupancy of O277      Constrained at        0.25 Check
PLAT300_ALERT_4_G Atom Site Occupancy of O278      Constrained at         0.3 Check
PLAT301_ALERT_3_G Main Residue  Disorder .............(Resd  1  )          1% Note
PLAT302_ALERT_4_G Anion/Solvent/Minor-Residue Disorder (Resd  2  )        13% Note
PLAT302_ALERT_4_G Anion/Solvent/Minor-Residue Disorder (Resd  3  )       100% Note
```

```
PLAT302_ALERT_4_G Anion/Solvent/Minor-Residue Disorder (Resd  4  )       100% Note
PLAT302_ALERT_4_G Anion/Solvent/Minor-Residue Disorder (Resd  5  )       100% Note
PLAT302_ALERT_4_G Anion/Solvent/Minor-Residue Disorder (Resd  6  )       100% Note
PLAT302_ALERT_4_G Anion/Solvent/Minor-Residue Disorder (Resd 21  )       100% Note
PLAT302_ALERT_4_G Anion/Solvent/Minor-Residue Disorder (Resd 22  )       100% Note
PLAT302_ALERT_4_G Anion/Solvent/Minor-Residue Disorder (Resd 23  )       100% Note
PLAT302_ALERT_4_G Anion/Solvent/Minor-Residue Disorder (Resd 24  )       100% Note
PLAT302_ALERT_4_G Anion/Solvent/Minor-Residue Disorder (Resd 25  )       100% Note
PLAT311_ALERT_2_G Isolated Disordered Oxygen Atom (No H's ?) .....       O272 Check
PLAT311_ALERT_2_G Isolated Disordered Oxygen Atom (No H's ?) .....       O273 Check
PLAT311_ALERT_2_G Isolated Disordered Oxygen Atom (No H's ?) .....       O251 Check
PLAT311_ALERT_2_G Isolated Disordered Oxygen Atom (No H's ?) .....       O255 Check
PLAT311_ALERT_2_G Isolated Disordered Oxygen Atom (No H's ?) .....       O252 Check
PLAT311_ALERT_2_G Isolated Disordered Oxygen Atom (No H's ?) .....       O253 Check
PLAT311_ALERT_2_G Isolated Disordered Oxygen Atom (No H's ?) .....       O274 Check
PLAT311_ALERT_2_G Isolated Disordered Oxygen Atom (No H's ?) .....       O277 Check
PLAT311_ALERT_2_G Isolated Disordered Oxygen Atom (No H's ?) .....       O278 Check
PLAT606_ALERT_4_G Solvent Accessible VOID(S) in Structure ........          ! Info
PLAT794_ALERT_5_G Tentative Bond Valency for Ce1       (III)     .       3.28 Info
PLAT794_ALERT_5_G Tentative Bond Valency for Ce2       (III)     .       3.36 Info
PLAT794_ALERT_5_G Tentative Bond Valency for Ce3       (III)     .       3.31 Info
PLAT794_ALERT_5_G Tentative Bond Valency for Mo2       (VI)      .       6.00 Info
PLAT794_ALERT_5_G Tentative Bond Valency for Mo3       (VI)      .       6.08 Info
PLAT794_ALERT_5_G Tentative Bond Valency for Mo4       (VI)      .       5.91 Info
PLAT794_ALERT_5_G Tentative Bond Valency for Mo5       (VI)      .       6.04 Info
PLAT794_ALERT_5_G Tentative Bond Valency for Mo6       (VI)      .       6.13 Info
PLAT794_ALERT_5_G Tentative Bond Valency for Mo7       (VI)      .       6.02 Info
PLAT794_ALERT_5_G Tentative Bond Valency for Mo13      (VI)      .       6.09 Info
PLAT794_ALERT_5_G Tentative Bond Valency for Mo14      (VI)      .       5.97 Info
PLAT794_ALERT_5_G Tentative Bond Valency for Mo20      (VI)      .       6.16 Info
PLAT794_ALERT_5_G Tentative Bond Valency for Mo21      (VI)      .       6.06 Info
PLAT794_ALERT_5_G Tentative Bond Valency for Mo22      (VI)      .       5.99 Info
PLAT794_ALERT_5_G Tentative Bond Valency for Mo23      (VI)      .       6.01 Info
PLAT794_ALERT_5_G Tentative Bond Valency for Mo24      (VI)      .       5.73 Info
PLAT794_ALERT_5_G Tentative Bond Valency for Mo25      (VI)      .       5.65 Info
PLAT794_ALERT_5_G Tentative Bond Valency for Mo29      (VI)      .       6.06 Info
PLAT794_ALERT_5_G Tentative Bond Valency for Mo33      (VI)      .       5.98 Info
PLAT794_ALERT_5_G Tentative Bond Valency for Mo34      (VI)      .       6.12 Info
PLAT794_ALERT_5_G Tentative Bond Valency for Mo35      (VI)      .       6.03 Info
PLAT794_ALERT_5_G Tentative Bond Valency for Mo41      (VI)      .       5.97 Info
PLAT794_ALERT_5_G Tentative Bond Valency for Mo43      (VI)      .       5.99 Info
PLAT794_ALERT_5_G Tentative Bond Valency for Mo45      (VI)      .       5.73 Info
PLAT794_ALERT_5_G Tentative Bond Valency for Mo46      (VI)      .       5.72 Info
PLAT794_ALERT_5_G Tentative Bond Valency for Mo49      (VI)      .       6.03 Info
PLAT794_ALERT_5_G Tentative Bond Valency for Mo52      (VI)      .       6.11 Info
PLAT794_ALERT_5_G Tentative Bond Valency for Mo57      (VI)      .       6.04 Info
PLAT794_ALERT_5_G Tentative Bond Valency for Mo58      (VI)      .       5.99 Info
PLAT794_ALERT_5_G Tentative Bond Valency for Mo59      (VI)      .       5.75 Info
PLAT794_ALERT_5_G Tentative Bond Valency for Mo64      (VI)      .       6.06 Info
PLAT794_ALERT_5_G Tentative Bond Valency for Mo68      (VI)      .       6.13 Info
PLAT794_ALERT_5_G Tentative Bond Valency for Mo70      (VI)      .       5.96 Info
PLAT860_ALERT_3_G Number of Least-Squares Restraints .............         92 Note
PLAT869_ALERT_4_G ALERTS Related to the Use of SQUEEZE Suppressed          ! Info
PLAT912_ALERT_4_G Missing # of FCF Reflections Above STh/L=  0.600        101 Note
PLAT913_ALERT_3_G Missing # of Very Strong Reflections in FCF ....          3 Note
PLAT933_ALERT_2_G Number of OMIT Records in Embedded .res File ...         11 Note
PLAT960_ALERT_3_G Number of Intensities with I  <   - 2*sig(I) ...         20 Check
```

---

5 **ALERT level A** = Most likely a serious problem - resolve or explain
16 **ALERT level B** = A potentially serious problem, consider carefully
30 **ALERT level C** = Check. Ensure it is not caused by an omission or oversight

```
93 ALERT level G = General information/check it is not something unexpected

 5 ALERT type 1 CIF construction/syntax error, inconsistent or missing data
57 ALERT type 2 Indicator that the structure model may be wrong or deficient
10 ALERT type 3 Indicator that the structure quality may be low
38 ALERT type 4 Improvement, methodology, query or suggestion
34 ALERT type 5 Informative message, check
```

It is advisable to attempt to resolve as many as possible of the alerts in all categories. Often the minor alerts point to easily fixed oversights, errors and omissions in your CIF or refinement strategy, so attention to these fine details can be worthwhile. In order to resolve some of the more serious problems it may be necessary to carry out additional measurements or structure refinements. However, the purpose of your study may justify the reported deviations and the more serious of these should normally be commented upon in the discussion or experimental section of a paper or in the "special_details" fields of the CIF. checkCIF was carefully designed to identify outliers and unusual parameters, but every test has its limitations and alerts that are not important in a particular case may appear. Conversely, the absence of alerts does not guarantee there are no aspects of the results needing attention. It is up to the individual to critically assess their own results and, if necessary, seek expert advice.

Publication of your CIF in IUCr journals

A basic structural check has been run on your CIF. These basic checks will be run on all CIFs submitted for publication in IUCr journals (*Acta Crystallographica*, *Journal of Applied Crystallography*, *Journal of Synchrotron Radiation*); however, if you intend to submit to *Acta Crystallographica Section C* or *E* or *IUCrData*, you should make sure that full publication checks are run on the final version of your CIF prior to submission.

Publication of your CIF in other journals

Please refer to the *Notes for Authors* of the relevant journal for any special instructions relating to CIF submission.

PLATON version of 03/06/2021; check.def file version of 02/06/2021

Datablock edu7571_sq - ellipsoid plot

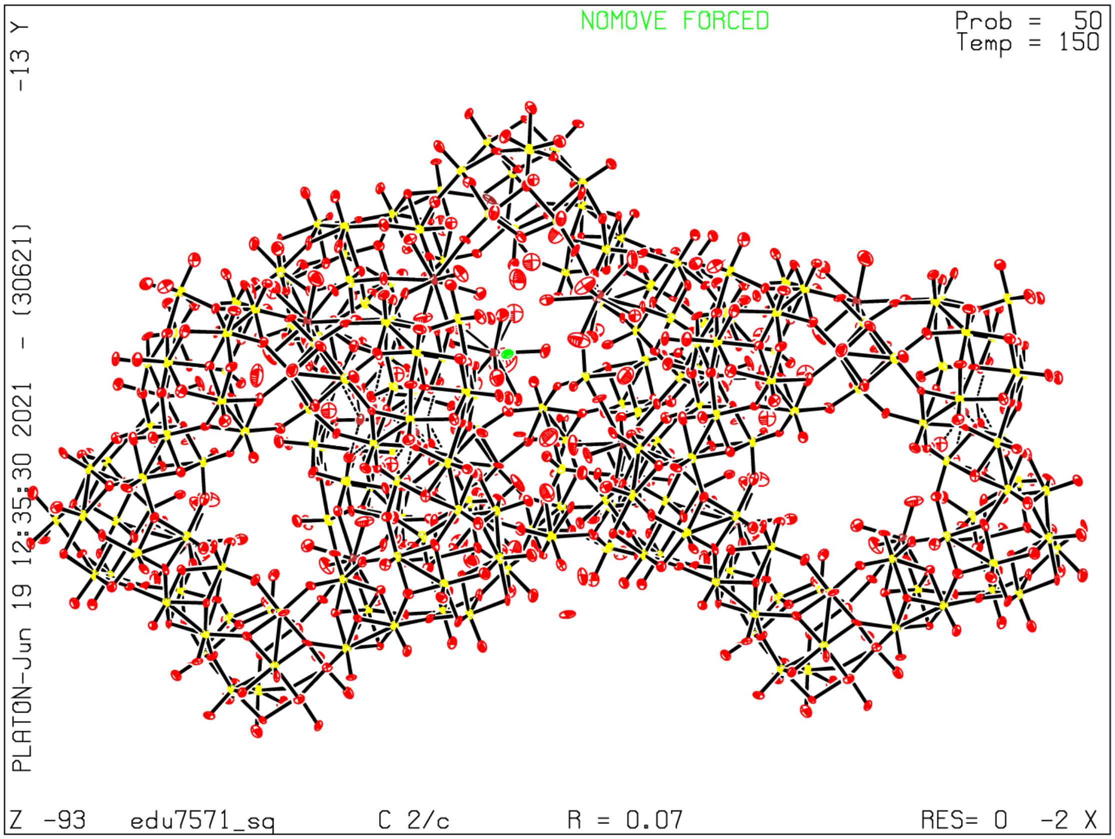

Supplement: Supplementary file 10 — Supporting Information [file ANIE-61-0-s001.pdf]
