# Supplementary material for: Engineering Highly Reduced Molybdenum Polyoxometalates via the Incorporation of d and f Block Metal Ions
Source: Angew Chem Int Ed Engl. 2022 Mar 23;61(21):e202201672. doi: 10.1002/anie.202201672 (PMC9401863; doi:10.1002/anie.202201672)

checkCIF/PLATON report

Structure factors have been supplied for datablock(s) edu7772_sq

THIS REPORT IS FOR GUIDANCE ONLY. IF USED AS PART OF A REVIEW PROCEDURE FOR PUBLICATION, IT SHOULD NOT REPLACE THE EXPERTISE OF AN EXPERIENCED CRYSTALLOGRAPHIC REFEREE.

No syntax errors found. CIF dictionary Interpreting this report

Datablock: edu7772_sq

```
Bond precision:  Nd- O = 0.0148 A        Wavelength=0.71073

Cell:            a=31.5796(7)    b=27.7392(4)        c=40.6670(8)
                 alpha=90        beta=110.138(2)     gamma=90
Temperature:     150 K
```

	Calculated	Reported
Volume	33446.2(12)	33446.2(12)
Space group	P 21/c	P 21/c
Hall group	-P 2ybc	-P 2ybc
Moiety formula	Mo140 Nd10 O479, 2(Nd0.67 O6.20), 3.4(O0.50), 12(O0.50), 2.8(Na	?
Sum formula	Mo140 Na2.80 Nd11.33 O504.80 [+ solvent]	H390 Mo70 Na3 Nd5.67 O395
Mr	23207.52	14315.24
Dx,g cm-3	2.304	2.843
Z	2	4
Mu (mm-1)	3.472	3.533
F000	21258.4	27452.0
F000’	20781.93	
h,k,lmax	38,34,50	38,34,50
Nref	65727	65667
Tmin,Tmax	0.919,0.942	0.848,0.984
Tmin’	0.798	

```
Correction method= # Reported T Limits: Tmin=0.848 Tmax=0.984
AbsCorr = GAUSSIAN

Data completeness= 0.999          Theta(max)= 26.000

R(reflections)= 0.0767( 30310)    wR2(reflections)= 0.2142( 65667)

S = 0.990                   Npar= 2964
```

The following ALERTS were generated. Each ALERT has the format
test-name_ALERT_alert-type_alert-level.
Click on the hyperlinks for more details of the test.

Alert level A

```
RINTA01_ALERT_3_A  The value of Rint is greater than 0.25
            Rint given   0.375
```

Author Response: poor crystal with weak diffraction

```
PLAT020_ALERT_3_A The Value of Rint is Greater Than 0.12 .........      0.375 Report
```

Author Response: poor crystal with weak diffraction

Alert level B

```
PLAT306_ALERT_2_B Isolated Oxygen Atom (H-atoms Missing ?) .......       O271 Check
PLAT306_ALERT_2_B Isolated Oxygen Atom (H-atoms Missing ?) .......       O272 Check
PLAT306_ALERT_2_B Isolated Oxygen Atom (H-atoms Missing ?) .......       O274 Check
PLAT306_ALERT_2_B Isolated Oxygen Atom (H-atoms Missing ?) .......       O275 Check
PLAT306_ALERT_2_B Isolated Oxygen Atom (H-atoms Missing ?) .......       O276 Check
PLAT910_ALERT_3_B Missing # of FCF Reflection(s) Below Theta(Min).         46 Note
PLAT975_ALERT_2_B Check Calcd Resid. Dens.  0.92A   From O272          1.82 eA-3
PLAT975_ALERT_2_B Check Calcd Resid. Dens.  0.79A   From O272          1.76 eA-3
PLAT975_ALERT_2_B Check Calcd Resid. Dens.  0.93A   From O232          1.64 eA-3
PLAT975_ALERT_2_B Check Calcd Resid. Dens.  0.83A   From O235          1.56 eA-3
PLAT975_ALERT_2_B Check Calcd Resid. Dens.  0.74A   From O238          1.54 eA-3
PLAT975_ALERT_2_B Check Calcd Resid. Dens.  0.79A   From O230          1.53 eA-3
```

Alert level C

```
PLAT077_ALERT_4_C Unitcell Contains Non-integer Number of Atoms ..     Please Check
PLAT202_ALERT_3_C Isotropic non-H Atoms in Anion/Solvent .........          1 Check
              O256
PLAT213_ALERT_2_C Atom O26              has ADP max/min Ratio .....        3.1 prolat
PLAT213_ALERT_2_C Atom O29              has ADP max/min Ratio .....        3.7 oblate
PLAT213_ALERT_2_C Atom O79              has ADP max/min Ratio .....        3.2 prolat
PLAT213_ALERT_2_C Atom O138             has ADP max/min Ratio .....        3.2 oblate
PLAT213_ALERT_2_C Atom O157             has ADP max/min Ratio .....        3.8 oblate
PLAT220_ALERT_2_C NonSolvent   Resd 1  O   Ueq(max)/Ueq(min) Range         5.8 Ratio
PLAT242_ALERT_2_C Low    'MainMol' Ueq as Compared to Neighbors of         Nd1 Check
PLAT242_ALERT_2_C Low    'MainMol' Ueq as Compared to Neighbors of         Nd2 Check
PLAT242_ALERT_2_C Low    'MainMol' Ueq as Compared to Neighbors of         Nd3 Check
PLAT242_ALERT_2_C Low    'MainMol' Ueq as Compared to Neighbors of         Nd4 Check
PLAT250_ALERT_2_C Large U3/U1 Ratio for Average U(i,j) Tensor ....         3.4 Note
PLAT905_ALERT_3_C Negative K value in the Analysis of Variance ...     -4.971 Report
PLAT911_ALERT_3_C Missing FCF Refl Between Thmin & STh/L=    0.600         14 Report
PLAT913_ALERT_3_C Missing # of Very Strong Reflections in FCF ....          7 Note
PLAT975_ALERT_2_C Check Calcd Resid. Dens.  1.01A   From O236          1.40 eA-3
PLAT975_ALERT_2_C Check Calcd Resid. Dens.  0.71A   From O235          1.34 eA-3
```

Alert level G

```
FORMU01_ALERT_2_G  There is a discrepancy between the atom counts in the
            _chemical_formula_sum and the formula from the _atom_site* data.
```

```
            Atom count from _chemical_formula_sum:H390 Mo70 Na3 Nd5.67 O395
            Atom count from the _atom_site data:  Mo70 Na1.4 Nd5.666699 O252.4
CELLZ01_ALERT_1_G Difference between formula and atom_site contents detected.
CELLZ01_ALERT_1_G ALERT: Large difference may be due to a
            symmetry error - see SYMMG tests
           From the CIF: _cell_formula_units_Z    4
           From the CIF: _chemical_formula_sum  H390 Mo70 Na3 Nd5.67 O395
           TEST: Compare cell contents of formula and atom_site data

           atom    Z*formula  cif sites diff
           H       1560.00      0.00 1560.00
           Mo       280.00    280.00    0.00
           Na        12.00      5.60    6.40
           Nd        22.68     22.67    0.01
           O       1580.00   1009.60  570.40
PLAT003_ALERT_2_G Number of Uiso or Uij Restrained non-H Atoms ...         16 Report
PLAT004_ALERT_5_G Polymeric Structure Found with Maximum Dimension          1 Info
PLAT041_ALERT_1_G Calc. and Reported SumFormula    Strings  Differ     Please Check
PLAT045_ALERT_1_G Calculated and Reported Z Differ by a Factor ...       0.50 Check
PLAT051_ALERT_1_G Mu(calc) and Mu(CIF) Ratio Differs from 1.0 by .       1.72 %
PLAT186_ALERT_4_G The CIF-Embedded .res File Contains ISOR Records          2 Report
PLAT300_ALERT_4_G Atom Site Occupancy of O230       Constrained at        0.7 Check
PLAT300_ALERT_4_G Atom Site Occupancy of O231       Constrained at        0.6 Check
PLAT300_ALERT_4_G Atom Site Occupancy of O232       Constrained at        0.6 Check
PLAT300_ALERT_4_G Atom Site Occupancy of O235       Constrained at        0.7 Check
PLAT300_ALERT_4_G Atom Site Occupancy of O236       Constrained at        0.7 Check
PLAT300_ALERT_4_G Atom Site Occupancy of O237       Constrained at        0.7 Check
PLAT300_ALERT_4_G Atom Site Occupancy of O238       Constrained at        0.5 Check
PLAT300_ALERT_4_G Atom Site Occupancy of Nd6        Constrained at     0.6667 Check
PLAT300_ALERT_4_G Atom Site Occupancy of O257       Constrained at        0.3 Check
PLAT300_ALERT_4_G Atom Site Occupancy of O259       Constrained at        0.3 Check
PLAT300_ALERT_4_G Atom Site Occupancy of O260       Constrained at        0.3 Check
PLAT300_ALERT_4_G Atom Site Occupancy of O261       Constrained at        0.3 Check
PLAT300_ALERT_4_G Atom Site Occupancy of O256       Constrained at        0.7 Check
PLAT300_ALERT_4_G Atom Site Occupancy of O258       Constrained at        0.5 Check
PLAT300_ALERT_4_G Atom Site Occupancy of O277       Constrained at        0.5 Check
PLAT300_ALERT_4_G Atom Site Occupancy of Na1        Constrained at        0.7 Check
PLAT300_ALERT_4_G Atom Site Occupancy of Na2        Constrained at        0.7 Check
PLAT301_ALERT_3_G Main Residue  Disorder .............(Resd  1  )         1% Note
PLAT302_ALERT_4_G Anion/Solvent/Minor-Residue Disorder (Resd  2  )        27% Note
PLAT302_ALERT_4_G Anion/Solvent/Minor-Residue Disorder (Resd  3  )       100% Note
PLAT302_ALERT_4_G Anion/Solvent/Minor-Residue Disorder (Resd  4  )       100% Note
PLAT302_ALERT_4_G Anion/Solvent/Minor-Residue Disorder (Resd 10  )       100% Note
PLAT302_ALERT_4_G Anion/Solvent/Minor-Residue Disorder (Resd 11  )       100% Note
PLAT302_ALERT_4_G Anion/Solvent/Minor-Residue Disorder (Resd 12  )       100% Note
PLAT311_ALERT_2_G Isolated Disordered Oxygen Atom (No H’s ?) .....       O257 Check
PLAT311_ALERT_2_G Isolated Disordered Oxygen Atom (No H’s ?) .....       O259 Check
PLAT311_ALERT_2_G Isolated Disordered Oxygen Atom (No H’s ?) .....       O260 Check
PLAT311_ALERT_2_G Isolated Disordered Oxygen Atom (No H’s ?) .....       O261 Check
PLAT311_ALERT_2_G Isolated Disordered Oxygen Atom (No H’s ?) .....       O256 Check
PLAT311_ALERT_2_G Isolated Disordered Oxygen Atom (No H’s ?) .....       O258 Check
PLAT311_ALERT_2_G Isolated Disordered Oxygen Atom (No H’s ?) .....       O277 Check
PLAT606_ALERT_4_G Solvent Accessible VOID(S) in Structure ........          ! Info
PLAT794_ALERT_5_G Tentative Bond Valency for Nd1       (II)      .       2.12 Info
PLAT794_ALERT_5_G Tentative Bond Valency for Nd4       (II)      .       2.04 Info
PLAT794_ALERT_5_G Tentative Bond Valency for Nd5       (II)      .       1.99 Info
PLAT794_ALERT_5_G Tentative Bond Valency for Mo10      (VI)      .       6.00 Info
PLAT794_ALERT_5_G Tentative Bond Valency for Mo11      (VI)      .       5.94 Info
PLAT794_ALERT_5_G Tentative Bond Valency for Mo13      (VI)      .       6.12 Info
PLAT794_ALERT_5_G Tentative Bond Valency for Mo14      (VI)      .       5.97 Info
PLAT794_ALERT_5_G Tentative Bond Valency for Mo17      (VI)      .       6.15 Info
PLAT794_ALERT_5_G Tentative Bond Valency for Mo18      (VI)      .       6.11 Info
```

```
PLAT794_ALERT_5_G Tentative Bond Valency for Mo19      (VI)      .       5.93 Info
PLAT794_ALERT_5_G Tentative Bond Valency for Mo20      (VI)      .       5.90 Info
PLAT794_ALERT_5_G Tentative Bond Valency for Mo21      (VI)      .       6.12 Info
PLAT794_ALERT_5_G Tentative Bond Valency for Mo23      (VI)      .       6.23 Info
PLAT794_ALERT_5_G Tentative Bond Valency for Mo25      (VI)      .       6.16 Info
PLAT794_ALERT_5_G Tentative Bond Valency for Mo31      (VI)      .       5.91 Info
PLAT794_ALERT_5_G Tentative Bond Valency for Mo32      (VI)      .       5.90 Info
PLAT794_ALERT_5_G Tentative Bond Valency for Mo33      (VI)      .       6.06 Info
PLAT794_ALERT_5_G Tentative Bond Valency for Mo34      (VI)      .       5.99 Info
PLAT794_ALERT_5_G Tentative Bond Valency for Mo35      (VI)      .       6.15 Info
PLAT794_ALERT_5_G Tentative Bond Valency for Mo37      (VI)      .       5.95 Info
PLAT794_ALERT_5_G Tentative Bond Valency for Mo42      (VI)      .       6.03 Info
PLAT794_ALERT_5_G Tentative Bond Valency for Mo44      (VI)      .       5.93 Info
PLAT794_ALERT_5_G Tentative Bond Valency for Mo46      (VI)      .       6.14 Info
PLAT794_ALERT_5_G Tentative Bond Valency for Mo48      (VI)      .       5.97 Info
PLAT794_ALERT_5_G Tentative Bond Valency for Mo52      (VI)      .       5.96 Info
PLAT794_ALERT_5_G Tentative Bond Valency for Mo53      (VI)      .       5.92 Info
PLAT794_ALERT_5_G Tentative Bond Valency for Mo54      (VI)      .       5.84 Info
PLAT794_ALERT_5_G Tentative Bond Valency for Mo55      (VI)      .       6.03 Info
PLAT794_ALERT_5_G Tentative Bond Valency for Mo61      (VI)      .       6.03 Info
PLAT794_ALERT_5_G Tentative Bond Valency for Mo62      (VI)      .       6.08 Info
PLAT794_ALERT_5_G Tentative Bond Valency for Mo63      (VI)      .       6.05 Info
PLAT794_ALERT_5_G Tentative Bond Valency for Mo65      (VI)      .       6.03 Info
PLAT794_ALERT_5_G Tentative Bond Valency for Mo68      (VI)      .       6.26 Info
PLAT860_ALERT_3_G Number of Least-Squares Restraints .............         96 Note
PLAT869_ALERT_4_G ALERTS Related to the Use of SQUEEZE Suppressed           ! Info
PLAT933_ALERT_2_G Number of OMIT Records in Embedded .res File ...         14 Note
PLAT960_ALERT_3_G Number of Intensities with I  <   - 2*sig(I) ...         57 Check
```

---

2 **ALERT level A** = Most likely a serious problem - resolve or explain
12 **ALERT level B** = A potentially serious problem, consider carefully
18 **ALERT level C** = Check. Ensure it is not caused by an omission or oversight
78 **ALERT level G** = General information/check it is not something unexpected

5 ALERT type 1 CIF construction/syntax error, inconsistent or missing data
34 ALERT type 2 Indicator that the structure model may be wrong or deficient
10 ALERT type 3 Indicator that the structure quality may be low
27 ALERT type 4 Improvement, methodology, query or suggestion
34 ALERT type 5 Informative message, check

It is advisable to attempt to resolve as many as possible of the alerts in all categories. Often the minor alerts point to easily fixed oversights, errors and omissions in your CIF or refinement strategy, so attention to these fine details can be worthwhile. In order to resolve some of the more serious problems it may be necessary to carry out additional measurements or structure refinements. However, the purpose of your study may justify the reported deviations and the more serious of these should normally be commented upon in the discussion or experimental section of a paper or in the "special_details" fields of the CIF. checkCIF was carefully designed to identify outliers and unusual parameters, but every test has its limitations and alerts that are not important in a particular case may appear. Conversely, the absence of alerts does not guarantee there are no aspects of the results needing attention. It is up to the individual to critically assess their own results and, if necessary, seek expert advice.

Publication of your CIF in IUCr journals

A basic structural check has been run on your CIF. These basic checks will be run on all CIFs submitted for publication in IUCr journals (*Acta Crystallographica*, *Journal of Applied Crystallography*, *Journal of Synchrotron Radiation*); however, if you intend to submit to *Acta Crystallographica Section C* or *E* or *IUCrData*, you should make sure that full publication checks are run on the final version of your CIF prior to submission.

Publication of your CIF in other journals

Please refer to the *Notes for Authors* of the relevant journal for any special instructions relating to CIF submission.

PLATON version of 03/06/2021; check.def file version of 02/06/2021

Datablock edu7772_sq - ellipsoid plot

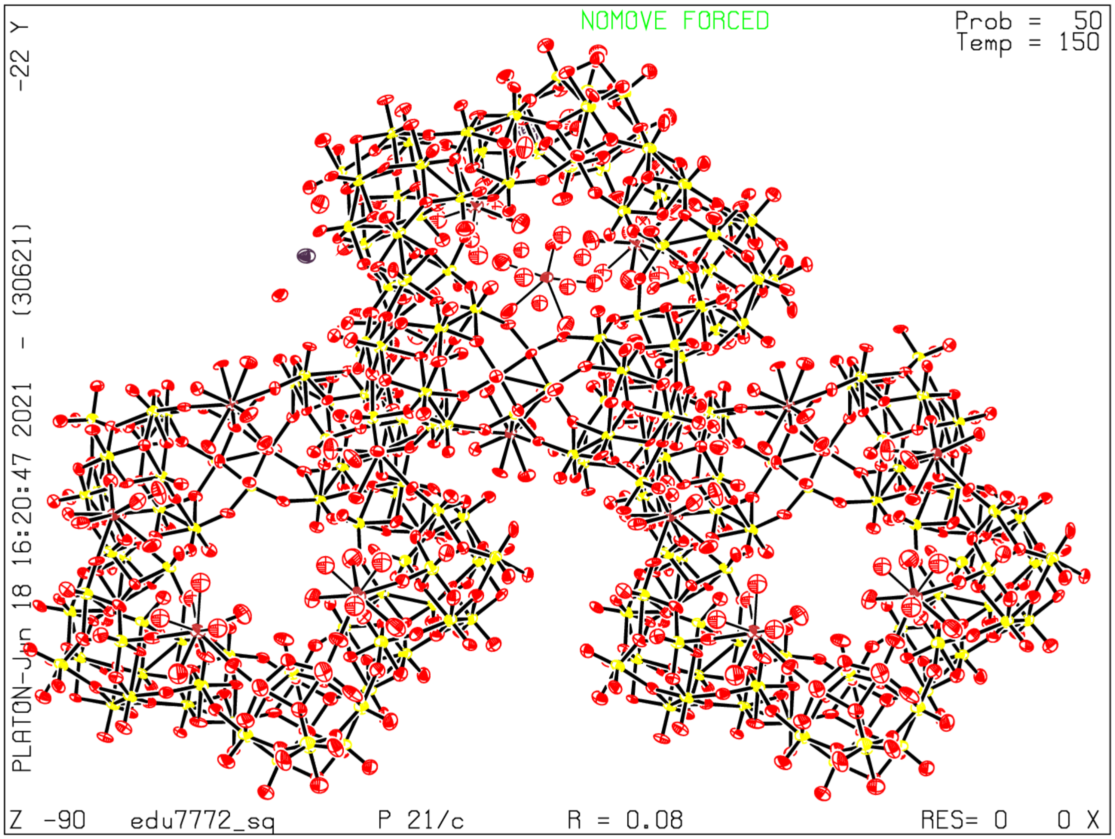

Supplement: Supplementary file 12 — Supporting Information [file ANIE-61-0-s008.pdf]
