# Supplementary material for: Engineering Highly Reduced Molybdenum Polyoxometalates via the Incorporation of d and f Block Metal Ions
Source: Angew Chem Int Ed Engl. 2022 Mar 23;61(21):e202201672. doi: 10.1002/anie.202201672 (PMC9401863; doi:10.1002/anie.202201672)

checkCIF/PLATON report

Structure factors have been supplied for datablock(s) edu6451_sq

THIS REPORT IS FOR GUIDANCE ONLY. IF USED AS PART OF A REVIEW PROCEDURE FOR PUBLICATION, IT SHOULD NOT REPLACE THE EXPERTISE OF AN EXPERIENCED CRYSTALLOGRAPHIC REFEREE.

No syntax errors found.   CIF dictionary   Interpreting this report

Datablock: edu6451_sq

Bond precision: Sm- O = 0.0247 A   Wavelength=0.71073

Cell:	a=20.8564(2)	b=26.5422(4)	c=73.4293(9)
	alpha=90	beta=95.396(2)	gamma=90
Temperature:	150 K		

	Calculated	Reported
Volume	40468.5(9)	40468.5(9)
Space group	P 21/n	P 21/n
Hall group	-P 2yn	-P 2yn
Moiety formula	Mo280 O963.60 Sm20, 4(O2 Sm0.50), 28.4(O) [+ solvent]	?
Sum formula	Mo280 O1000 Sm22 [+ solvent]	H530 Mo70 Na3.50 O465 Sm5.50
Mr	46171.14	15597.40
Dx,g cm-3	1.895	2.560
Z	1	4
Mu (mm-1)	2.939	3.015
F000	21124.0	30278.0
F000’	20648.09	
h,k,lmax	25,32,89	25,32,89
Nref	77268	77389
Tmin,Tmax	0.875,0.947	0.943,1.000
Tmin’	0.765	

Correction method= # Reported T Limits: Tmin=0.943 Tmax=1.000
AbsCorr = GAUSSIAN

Data completeness= 1.002   Theta(max)= 25.726

R(reflections)= 0.1455(55054)   wR2(reflections)= 0.3704(77389)

S = 1.031   Npar= 2914

The following ALERTS were generated. Each ALERT has the format
test-name_ALERT_alert-type_alert-level.
Click on the hyperlinks for more details of the test.

Alert level A

```
PLAT910_ALERT_3_A Missing # of FCF Reflection(s) Below Theta(Min).         56 Note
```

Author Response: poor crystal with weak diffraction

Alert level B

```
RINTA01_ALERT_3_B  The value of Rint is greater than 0.18
            Rint given   0.238
PLAT020_ALERT_3_B The Value of Rint is Greater Than 0.12 .........      0.238 Report
PLAT084_ALERT_3_B High wR2 Value (i.e. > 0.25) ...................       0.37 Report
              O2        O7        O8        O17       O19               etc.
PLAT213_ALERT_2_B Atom O15             has ADP max/min Ratio .....        4.1 prolat
PLAT306_ALERT_2_B Isolated Oxygen Atom (H-atoms Missing ?) .......       O261 Check
PLAT306_ALERT_2_B Isolated Oxygen Atom (H-atoms Missing ?) .......       O262 Check
PLAT306_ALERT_2_B Isolated Oxygen Atom (H-atoms Missing ?) .......       O263 Check
PLAT306_ALERT_2_B Isolated Oxygen Atom (H-atoms Missing ?) .......       O264 Check
```

Alert level C

```
PLAT082_ALERT_2_C High R1 Value .................................       0.15 Report
PLAT213_ALERT_2_C Atom O40             has ADP max/min Ratio .....        3.1 oblate
PLAT213_ALERT_2_C Atom O52             has ADP max/min Ratio .....        3.6 oblate
PLAT213_ALERT_2_C Atom O82             has ADP max/min Ratio .....        3.2 oblate
PLAT213_ALERT_2_C Atom O109            has ADP max/min Ratio .....        3.2 oblate
PLAT220_ALERT_2_C NonSolvent   Resd 1  O   Ueq(max)/Ueq(min) Range        5.7 Ratio
PLAT241_ALERT_2_C High   'MainMol' Ueq as Compared to Neighbors of       O104 Check
PLAT241_ALERT_2_C High   'MainMol' Ueq as Compared to Neighbors of       O147 Check
PLAT241_ALERT_2_C High   'MainMol' Ueq as Compared to Neighbors of       O153 Check
PLAT241_ALERT_2_C High   'MainMol' Ueq as Compared to Neighbors of       O160 Check
PLAT241_ALERT_2_C High   'MainMol' Ueq as Compared to Neighbors of       O201 Check
PLAT242_ALERT_2_C Low    'MainMol' Ueq as Compared to Neighbors of        Sm5 Check
PLAT242_ALERT_2_C Low    'MainMol' Ueq as Compared to Neighbors of        O36 Check
PLAT242_ALERT_2_C Low    'MainMol' Ueq as Compared to Neighbors of        O85 Check
PLAT242_ALERT_2_C Low    'MainMol' Ueq as Compared to Neighbors of       O158 Check
PLAT906_ALERT_3_C Large K Value in the Analysis of Variance ......      2.677 Check
PLAT911_ALERT_3_C Missing FCF Refl Between Thmin & STh/L=    0.600          6 Report
PLAT913_ALERT_3_C Missing # of Very Strong Reflections in FCF ....          7 Note
```

Alert level G

```
FORMU01_ALERT_2_G  There is a discrepancy between the atom counts in the
            _chemical_formula_sum and the formula from the _atom_site* data.
            Atom count from _chemical_formula_sum:H530 Mo70 Na3.5 O465 Sm5.5
            Atom count from the _atom_site data:  Mo70 O250 Sm5.5
CELLZ01_ALERT_1_G Difference between formula and atom_site contents detected.
CELLZ01_ALERT_1_G ALERT: Large difference may be due to a
            symmetry error - see SYMMG tests
           From the CIF: _cell_formula_units_Z    4
           From the CIF: _chemical_formula_sum  H530 Mo70 Na3.50 O465 Sm5.50
           TEST: Compare cell contents of formula and atom_site data

           atom    Z*formula  cif sites diff
```

```
           H       2120.00      0.00 2120.00
           Mo       280.00    280.00    0.00
           Na        14.00      0.00   14.00
           O       1860.00   1000.00  860.00
           Sm        22.00     22.00    0.00
PLAT003_ALERT_2_G Number of Uiso or Uij Restrained non-H Atoms ...        243 Report
PLAT004_ALERT_5_G Polymeric Structure Found with Maximum Dimension          1 Info
PLAT041_ALERT_1_G Calc. and Reported SumFormula    Strings  Differ     Please Check
PLAT045_ALERT_1_G Calculated and Reported Z Differ by a Factor ...       0.25 Check
PLAT051_ALERT_1_G Mu(calc) and Mu(CIF) Ratio Differs from 1.0 by .       2.53 %
PLAT083_ALERT_2_G SHELXL Second Parameter in WGHT  Unusually Large    6552.69 Why ?
PLAT186_ALERT_4_G The CIF-Embedded .res File Contains ISOR Records          1 Report
PLAT300_ALERT_4_G Atom Site Occupancy of O241       Constrained at        0.7 Check
PLAT300_ALERT_4_G Atom Site Occupancy of O242       Constrained at        0.7 Check
PLAT300_ALERT_4_G Atom Site Occupancy of O243       Constrained at        0.7 Check
PLAT300_ALERT_4_G Atom Site Occupancy of O249       Constrained at        0.8 Check
PLAT300_ALERT_4_G Atom Site Occupancy of Sm6        Constrained at        0.5 Check
PLAT300_ALERT_4_G Atom Site Occupancy of O251       Constrained at        0.7 Check
PLAT300_ALERT_4_G Atom Site Occupancy of O253       Constrained at        0.8 Check
PLAT300_ALERT_4_G Atom Site Occupancy of O255       Constrained at        0.8 Check
PLAT300_ALERT_4_G Atom Site Occupancy of O256       Constrained at        0.4 Check
PLAT300_ALERT_4_G Atom Site Occupancy of O257       Constrained at        0.4 Check
PLAT301_ALERT_3_G Main Residue  Disorder .............(Resd  1  )         1% Note
PLAT302_ALERT_4_G Anion/Solvent/Minor-Residue Disorder (Resd  2  )        20% Note
PLAT302_ALERT_4_G Anion/Solvent/Minor-Residue Disorder (Resd  3  )       100% Note
PLAT302_ALERT_4_G Anion/Solvent/Minor-Residue Disorder (Resd  4  )       100% Note
PLAT302_ALERT_4_G Anion/Solvent/Minor-Residue Disorder (Resd  5  )       100% Note
PLAT302_ALERT_4_G Anion/Solvent/Minor-Residue Disorder (Resd 10  )       100% Note
PLAT302_ALERT_4_G Anion/Solvent/Minor-Residue Disorder (Resd 11  )       100% Note
PLAT311_ALERT_2_G Isolated Disordered Oxygen Atom (No H’s ?) .....       O251 Check
PLAT311_ALERT_2_G Isolated Disordered Oxygen Atom (No H’s ?) .....       O253 Check
PLAT311_ALERT_2_G Isolated Disordered Oxygen Atom (No H’s ?) .....       O255 Check
PLAT311_ALERT_2_G Isolated Disordered Oxygen Atom (No H’s ?) .....       O256 Check
PLAT311_ALERT_2_G Isolated Disordered Oxygen Atom (No H’s ?) .....       O257 Check
PLAT606_ALERT_4_G Solvent Accessible VOID(S) in Structure ........          ! Info
PLAT794_ALERT_5_G Tentative Bond Valency for Sm1       (III)     .       3.33 Info
PLAT794_ALERT_5_G Tentative Bond Valency for Sm2       (III)     .       3.18 Info
PLAT794_ALERT_5_G Tentative Bond Valency for Sm3       (III)     .       3.27 Info
PLAT794_ALERT_5_G Tentative Bond Valency for Mo1       (VI)      .       5.59 Info
PLAT794_ALERT_5_G Tentative Bond Valency for Mo3       (VI)      .       5.99 Info
PLAT794_ALERT_5_G Tentative Bond Valency for Mo4       (VI)      .       5.82 Info
PLAT794_ALERT_5_G Tentative Bond Valency for Mo5       (VI)      .       5.56 Info
PLAT794_ALERT_5_G Tentative Bond Valency for Mo6       (VI)      .       6.19 Info
PLAT794_ALERT_5_G Tentative Bond Valency for Mo7       (VI)      .       5.99 Info
PLAT794_ALERT_5_G Tentative Bond Valency for Mo16      (VI)      .       6.01 Info
PLAT794_ALERT_5_G Tentative Bond Valency for Mo17      (VI)      .       5.90 Info
PLAT794_ALERT_5_G Tentative Bond Valency for Mo18      (VI)      .       6.14 Info
PLAT794_ALERT_5_G Tentative Bond Valency for Mo19      (VI)      .       5.70 Info
PLAT794_ALERT_5_G Tentative Bond Valency for Mo20      (VI)      .       5.68 Info
PLAT794_ALERT_5_G Tentative Bond Valency for Mo21      (VI)      .       6.23 Info
PLAT794_ALERT_5_G Tentative Bond Valency for Mo26      (VI)      .       6.11 Info
PLAT794_ALERT_5_G Tentative Bond Valency for Mo27      (VI)      .       5.87 Info
PLAT794_ALERT_5_G Tentative Bond Valency for Mo30      (VI)      .       6.00 Info
PLAT794_ALERT_5_G Tentative Bond Valency for Mo32      (VI)      .       5.92 Info
PLAT794_ALERT_5_G Tentative Bond Valency for Mo34      (VI)      .       6.03 Info
PLAT794_ALERT_5_G Tentative Bond Valency for Mo37      (VI)      .       6.30 Info
PLAT794_ALERT_5_G Tentative Bond Valency for Mo40      (VI)      .       6.52 Info
PLAT794_ALERT_5_G Tentative Bond Valency for Mo42      (VI)      .       5.53 Info
PLAT794_ALERT_5_G Tentative Bond Valency for Mo48      (VI)      .       5.89 Info
PLAT794_ALERT_5_G Tentative Bond Valency for Mo49      (VI)      .       5.86 Info
PLAT794_ALERT_5_G Tentative Bond Valency for Mo51      (VI)      .       5.79 Info
PLAT794_ALERT_5_G Tentative Bond Valency for Mo53      (VI)      .       5.74 Info
```

```
PLAT794_ALERT_5_G Tentative Bond Valency for Mo55      (VI)      .       6.11 Info
PLAT794_ALERT_5_G Tentative Bond Valency for Mo59      (VI)      .       6.63 Info
PLAT794_ALERT_5_G Tentative Bond Valency for Mo64      (VI)      .       6.19 Info
PLAT794_ALERT_5_G Tentative Bond Valency for Mo65      (V)       .       5.49 Info
PLAT794_ALERT_5_G Tentative Bond Valency for Mo66      (VI)      .       5.78 Info
PLAT794_ALERT_5_G Tentative Bond Valency for Mo67      (VI)      .       5.97 Info
PLAT860_ALERT_3_G Number of Least-Squares Restraints .............       1458 Note
PLAT869_ALERT_4_G ALERTS Related to the Use of SQUEEZE Suppressed           ! Info
PLAT870_ALERT_4_G ALERTS Related to Twinning Effects Suppressed ..          ! Info
PLAT912_ALERT_4_G Missing # of FCF Reflections Above STh/L=  0.600        436 Note
PLAT933_ALERT_2_G Number of OMIT Records in Embedded .res File ...          6 Note
PLAT960_ALERT_3_G Number of Intensities with I  <   - 2*sig(I) ...          2 Check
PLAT965_ALERT_2_G The SHELXL WEIGHT Optimisation has not Converged     Please Check
```

```
  1 ALERT level A = Most likely a serious problem - resolve or explain
  8 ALERT level B = A potentially serious problem, consider carefully
 18 ALERT level C = Check. Ensure it is not caused by an omission or oversight
 73 ALERT level G = General information/check it is not something unexpected

  5 ALERT type 1 CIF construction/syntax error, inconsistent or missing data
 30 ALERT type 2 Indicator that the structure model may be wrong or deficient
 10 ALERT type 3 Indicator that the structure quality may be low
 21 ALERT type 4 Improvement, methodology, query or suggestion
 34 ALERT type 5 Informative message, check
```

It is advisable to attempt to resolve as many as possible of the alerts in all categories. Often the minor alerts point to easily fixed oversights, errors and omissions in your CIF or refinement strategy, so attention to these fine details can be worthwhile. In order to resolve some of the more serious problems it may be necessary to carry out additional measurements or structure refinements. However, the purpose of your study may justify the reported deviations and the more serious of these should normally be commented upon in the discussion or experimental section of a paper or in the "special_details" fields of the CIF. checkCIF was carefully designed to identify outliers and unusual parameters, but every test has its limitations and alerts that are not important in a particular case may appear. Conversely, the absence of alerts does not guarantee there are no aspects of the results needing attention. It is up to the individual to critically assess their own results and, if necessary, seek expert advice.

Publication of your CIF in IUCr journals

A basic structural check has been run on your CIF. These basic checks will be run on all CIFs submitted for publication in IUCr journals (*Acta Crystallographica*, *Journal of Applied Crystallography*, *Journal of Synchrotron Radiation*); however, if you intend to submit to *Acta Crystallographica Section C* or *E* or *IUCrData*, you should make sure that full publication checks are run on the final version of your CIF prior to submission.

Publication of your CIF in other journals

Please refer to the *Notes for Authors* of the relevant journal for any special instructions relating to CIF submission.

PLATON version of 03/06/2021; check.def file version of 02/06/2021

Datablock edu6451_sq - ellipsoid plot

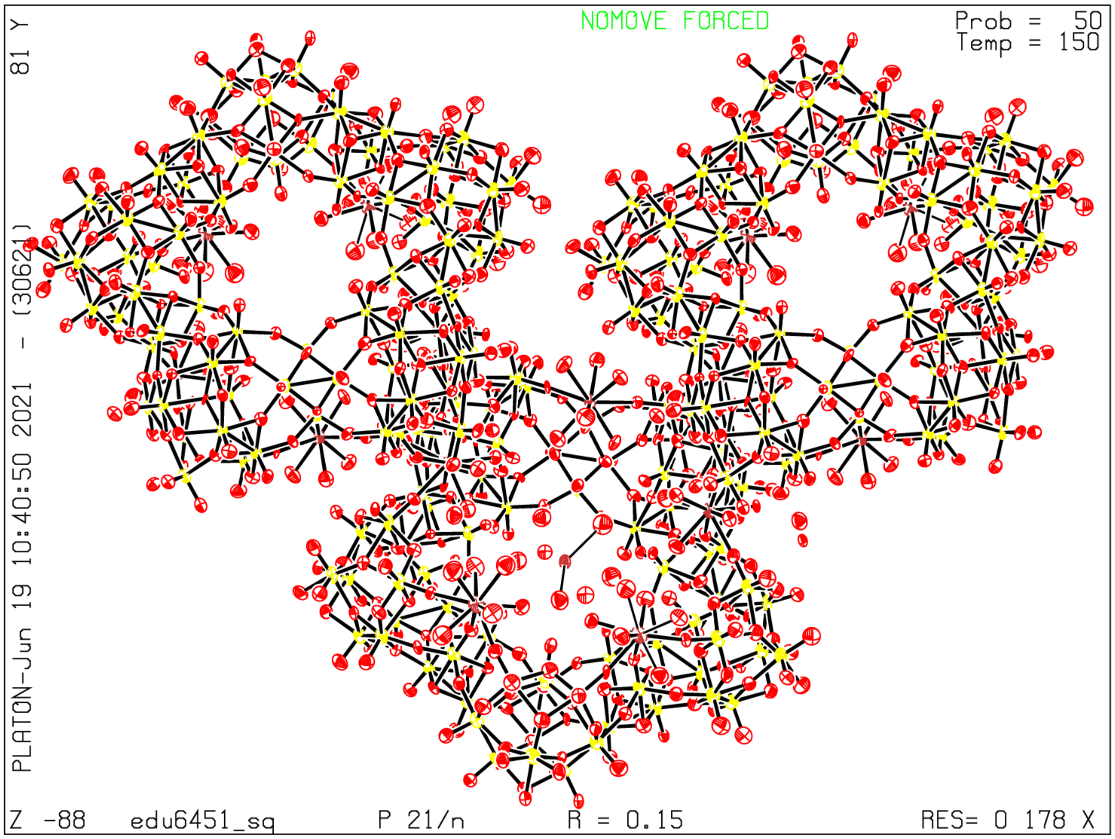

Supplement: Supplementary file 14 — Supporting Information [file ANIE-61-0-s012.pdf]
