# Supplementary material for: Engineering Highly Reduced Molybdenum Polyoxometalates via the Incorporation of d and f Block Metal Ions
Source: Angew Chem Int Ed Engl. 2022 Mar 23;61(21):e202201672. doi: 10.1002/anie.202201672 (PMC9401863; doi:10.1002/anie.202201672)
Supplement: Supplementary file 15 — Supporting Information [file ANIE-61-0-s002.pdf]

Angewandte Chemie
Eine Zeitschrift der Gesellschaft Deutscher Chemiker

Supporting Information

Engineering Highly Reduced Molybdenum Polyoxometalates via the Incorporation of *d* and *f* Block Metal Ions

*E. G. Ribó, N. L. Bell, D.-L. Long, L. Cronin**

Engineering Highly Reduced Molybdenum Polyoxometalates via the Incorporation of *d* and *f* Block Metal Ions.

Eduard Garrido Ribó, Nicola L. Bell, De-Liang Long, and Leroy Cronin *

School of Chemistry, University of Glasgow, University Avenue, Glasgow, G12 8QQ, United Kingdom

Table of Contents

1. Materials and instrumentation 2

2. Synthetic procedures 3

3. Formula determination 4

4. Lanthanide separation 5

5. Crystallographic data 7

6. Bond valence sum (BVS) studies 12

7. Structure descriptions 19

8. {Mo_{70}} star VS {Mo_{240}} cage 24

9. Thermogravimetric analysis (TGA) 27

10. Infrared spectroscopy (IR) 30

11. UV-VIS spectrums 31

12. References 31

1. Materials and instrumentation

All reagents and solvents were purchased from commercial sources and used as received. The vials used for the synthesis were 20 mL, 22.5 mm x 75.5 mm, clear glass with screw top caps. The lids were 18 mm x 11 mm stainless steel magnetic with a 1.6 mm thick PTFE-faced butyl septum. Both were purchased from SIGMA-Aldrich.

Elemental Analyses: Element analyses for Mo, La, Ce, Pr, Ni and Na were performed on a Leeman inductivity-coupled plasma (ICP) spectrometer while hydrogen content was determined by the microanalysis services within the School of Chemistry, University of Glasgow using an EA 1110 CHNS, CE-440 Elemental Analyzer.

Single Crystal X-Ray Diffraction: A suitable single crystal was selected and mounted onto a rubber loop using Fomblin or Parabar oil. Single-crystal datasets and unit cells for compound **1-6** were collected at 150(2) K on a Rigaku XtaLAB Synergy R HyPix-Arc diffractometer equipped with a graphite monochromator (λ (MoKα) = 0.71073 Å) on a microfocus X-ray source of rotating anode (50 kV, 24 mA). Data collection and reduction were performed using CrysAlisPro software package. Structure solution and refinement were carried out by SHELXT-2018/3[1] and SHELXL-2018/3[1] using Olex2[2] and finalized using WinGX.[3] Most of the non-hydrogen atoms (including those disordered) were anisotropically refined. Corrections for incident and diffracted beam absorption effects were applied using analytical numeric absorption correction on multifaceted crystal models.[4]

pH Measurements: Measurements were taken on a Hanna Instruments HI 9025C microcomputer pH meter, with a BCH combination pH electrode (309-1065) and HI 7669/2W temperature probe.

Infrared Spectroscopy: All samples were collected in transmission mode using a Thermo Scientific Nicolet iS5 spectrometer fitted with a Specac Golden Gate ATR.

UV-vis Spectroscopy: UV-vis spectra were recorded on a JASCO V-670 spectrophotometer using 0.5 cm and 1 cm pathlength cuvettes

Thermogravimetric Analysis: Analysis for characterisation was performed on a TA Instruments Q 500 Thermogravimetric analyser under air flow with a heating rate of 10 °C min^{-1} up to 1000 °C. Analysis for Solution Studies were performed on TA Discovery TGA 550 where ~5 mg solid samples were loaded onto a platinum pan, the weight-temperature changes were recorded under N_2 protection.

Oven: Samples were heated in an LTE OP60-UF oven equipped with fan circulation.

2. Synthetic procedures

{$Mo_{64}Ni_8La_6$}, **1**

In a 20mL vial equipped with a magnetic stirrer bar, $Na_2MoO_4\cdot 2H_2O$ (50 mg, 0.2 mmol) and $NiCl_2\cdot 6H_2O$ (95 mg, 0.4 mmol) were dissolved in 1.5 mL of H_2O. The mixture was then acidified by adding 1 mL of 6 M $HClO_4$. This was followed by the addition of $LaCl_3\cdot 7H_2O$ (30 mg, 0.08 mmol) and $N_2H_4\cdot 2HCl$ (5 mg, 0.05 mmol) dissolved in 1.1 mL of H_2O. The mixture was sealed with a metallic lid with a rubber septum and heated at ~ 90°C for ~2h. The mixture changes from light green to a red-brown colour. Then, the pH is increased slowly by first, adding 0.95 mL of 6 M NaOH at 1 mL/min, which produces yet another colour change to deep blue, and then 1 M NaOH (approx. 0.35 mL) at 0.3 mL/min to bring the final pH to 2.8. The sample is then degassed in a sonicator for 10min, flushed with Ar gas and sealed again. The sample is left in an oven at 100°C undisturbed for 4 days for crystal growth. Big red rod-like crystals of compound **1** were collected by first washing the solid with iced cold water and removing the suspended impurities and then by filtration through a filter with 100 µm pore size. The crystals were then left to dry in a fume hood cupboard for 2 days. Yield: 5.86 mg (15%, based on Mo). Elemental analysis, $Mo_{64}Ni_8La_6Na_9ClO_{289}H_{196}$ M_w = 12507.2, calc. (%): Mo 49.09, Ni 3.75, La 6.66, Na 1.65, H 1.58; found (%): Mo 45.15, Ni 4.33, La 7.04, Na 1.53, H 1.56.

{$Mo_{64}Ni_8Ce_6$}, **2**

Compound **2** was obtained following the same synthetic procedure as for compound **1** with the difference of using $CeCl_3\cdot 7H_2O$ (30 mg, 0.08 mmol) instead of $LaCl_3\cdot 7H_2O$. Yield: 3.91 mg (10%, based on Mo). Elemental analysis, $Mo_{64}Ni_8Ce_6Na_9ClO_{289}H_{196}$ M_w = 12514.5, calc. (%): Mo 49.07, Ni 3.75, Ce 6.72, Na 1.65, H 1.58; found (%): Mo 45.73, Ni 3.99, Ce 8.23, Na 1.47, H 1.41.

{$Mo_{64}Ni_8Pr_6$}, **3**

Compound **3** was obtained following the same synthetic procedure as for compound **1** with the difference of using $PrCl_3\cdot 6H_2O$ (30 mg, 0.08 mmol) instead of $LaCl_3\cdot 7H_2O$. Yield: 2.64 mg (7%, based on Mo). Elemental analysis, $Mo_{64}Ni_8Pr_6Na_9ClO_{264}H_{146}$ M_w = 12068.8, calc. (%): Mo 50.87, Ni 3.89, Pr 7.00, Na 1.71, H 1.22; found (%): Mo 46.18, Ni 3.87, Pr 8.01, Na 1.50, H 1.52.

{$Mo_{70}Ce_{4.5}$}, **4**

In a 20mL vial equipped with a magnetic stirrer bar, $Na_2MoO_4\cdot 2H_2O$ (50 mg, 0.2 mmol) and $NiCl_2\cdot 6H_2O$ (95 mg, 0.4 mmol) were dissolved in 1.5 mL of H_2O. The mixture was then acidified by adding 1 mL of 6 M $HClO_4$. This was followed by the addition of $CeCl_3\cdot 7H_2O$ (30 mg, 0.08 mmol) and $N_2H_4\cdot 2HCl$ (3 mg, 0.03 mmol) dissolved in 1.1 mL of H_2O. The mixture was sealed with a metallic lid with a rubber septum and heated at ~ 90 °C for ~ 2 h. The mixture changes from light green to a red-brown colour. Then, the pH

is increased slowly by first, adding 0.95 mL of 6 M NaOH at 1 mL/min, which produces yet another colour change to deep blue, and then 0.2 mL 1 M NaOH at 0.3 mL/min. The sample is then degassed in a sonicator for 10 min, flushed with Ar gas and sealed again. The sample is left in an oven at 100°C undisturbed for 4 days for crystal growth. Small red rod-like crystals of compound **4** appeared after 4 days.

{$Mo_{70}Nd_5$}, **5**

Compound **5** was obtained following the same synthetic procedure as for compound **4** with the difference of using $NdCl_3 \cdot 6H_2O$ (30 mg, 0.08 mmol) instead of $CeCl_3 \cdot 7H_2O$.

{$Mo_{70}Sm_5$}, **6**

Compound **6** was obtained following the same synthetic procedure as for compound **4** with the difference of using $SmCl_3 \cdot 6H_2O$ (30 mg, 0.08 mmol) instead of $CeCl_3 \cdot 7H_2O$.

3. Formula determination

The determination of the formulas of the mix-valence Mo clusters has been well established in the past and requires a series of analytical techniques including IR and UV−vis spectroscopy, bond valence sum analysis (BVS), elemental analysis and thermogravimetric analysis (TGA), in addition to single-crystal X-ray diffraction analysis.[5] Here **1** was selected to exemplify the general approach used to determine the formula of all the compounds **1** to **3**.

Firstly, BVS calculations were carried out on all the Mo and O centres, revealing that **1** is composed of a 52-electron reduced anionic cubic structure containing 26 singly protonated oxygen atoms.[6] BVS indicated that the reduced Mo centres are localised at the {Mo^V_2} pairs that have a Mo-Mo bond around 2.5 Å, consistent with previous work. [7] UV−vis spectroscopy showed an absorption band centred at around 310 nm, which can be attributed to the Mo-Mo charge transfer and the IR showed characteristic bands that can be assigned to the symmetric and asymmetric bending of O-Mo-O bridges at 959 cm^{-1} and 878 cm^{-1}, respectively. A strong broad peak around 1100 cm^{-1} can be assigned to both Mo=O bonds scattered throughout the structure and to a ClO^{4-} anion present in the cavity at the centre of the cluster. Finally, a peak at 704 cm^{-1} can be attributed to La-OH_2 bond stretches. Elemental analysis confirms the framework of **1** consists of a Mo:Ni:La ratio of 64:8:6. This is consistent with the structural refinement done using the single-crystal X-ray diffraction data. Taking into consideration of the information obtained from the calculations

above, along with elemental analysis, it is possible to determine the main framework and overall charge for **1** as $[Mo_{64}Ni_8La_6H_{26}O_{200}(H_2O)_{30}]^{8-}$ plus a charge separated ClO_4^-.[8] The charge is countered with nine Na atoms, as confirmed by elemental analysis and is consistent with the protonation level determined by BVS. Finally, the TGA curve of **1** exhibits a total weight loss of 11.5% from r.t. to 200 °C, which corresponds to ~85 water molecules that include 30 ligand water molecules on the cluster. On the basis of the discussion above, the formula of **1** could therefore be determined as $Na_9[Mo_{64}Ni_8La_6H_{26}O_{200}(H_2O)_{30}][ClO_4]\cdot 55H_2O$. Formulae for compounds **2** and **3** were determined in the same way as for **1**.

Formulae of compounds **4**, **5** and **6** were primarily determined by crystallography due to difficulty in isolating enough materials for chemical analyses. The contents of Ni, lanthanide, Cl and Mo were found in structure refinements with occupancies refined freely first and then fixed. The number of protons on clusters were tentatively determined from BVS calculated for oxo ligand protonation for compound **4** and **5**. For compound **6**, due to poor crystal diffraction data, no BVS calculation was performed but the number of protons was adopted from compound **4** and **5** based on structure similarity.

4. Lanthanide separation

Ln Mixtures synthesis

For all binary mixtures the same procedure was followed

In a 20 mL vial equipped with a magnetic stirrer bar, $Na_2MoO_4\cdot 2H_2O$ (50 mg, 0.2 mmol) and $NiCl_2\cdot 6H_2O$ (95 mg, 0.4 mmol) were dissolved in 1.5 mL of H_2O. The mixture was then acidified by adding 1 mL of 6 M $HClO_4$. This was followed by the addition of $LnCl_3\cdot nH_2O$ (0.08 mmol), $Ln_2Cl_3\cdot nH_2O$ (0.08 mmol) and $N_2H_4\cdot 2HCl$ (5 mg, 0.05 mmol) dissolved in 1.1 mL of H_2O. The mixture was sealed with a metallic lid with a rubber septum and heated at ~ 90 °C for ~ 2 h. The mixture changes from light green to a red-brown colour. Then, the pH is increased slowly by first, adding 0.95 mL of 6 M NaOH at 1 mL/min, which produces yet another colour change to deep blue, and then 1 M NaOH (approx. 0.35 mL) at 0.3 mL/min to bring the final pH to 2.8. The sample is then degassed in a sonicator for 10 min, flushed with Ar gas and sealed again. The sample is left in an oven at 100°C undisturbed for 4 days for crystal growth. Big red rod-like crystals of were collected were collected by first washing the solid with iced cold water and removing the suspended impurities and then by filtration through a filter with 100 µm pore size. The crystals were

then left to dry in a fume hood cupboard for 2 days. In case of the 3:1 mixture, the same procedure was followed with the exception of using 0.06 mmol of $LnCl_3 \cdot nH_2O$ and 0.02 mmol of $Ln_2Cl_3 \cdot nH_2O$.

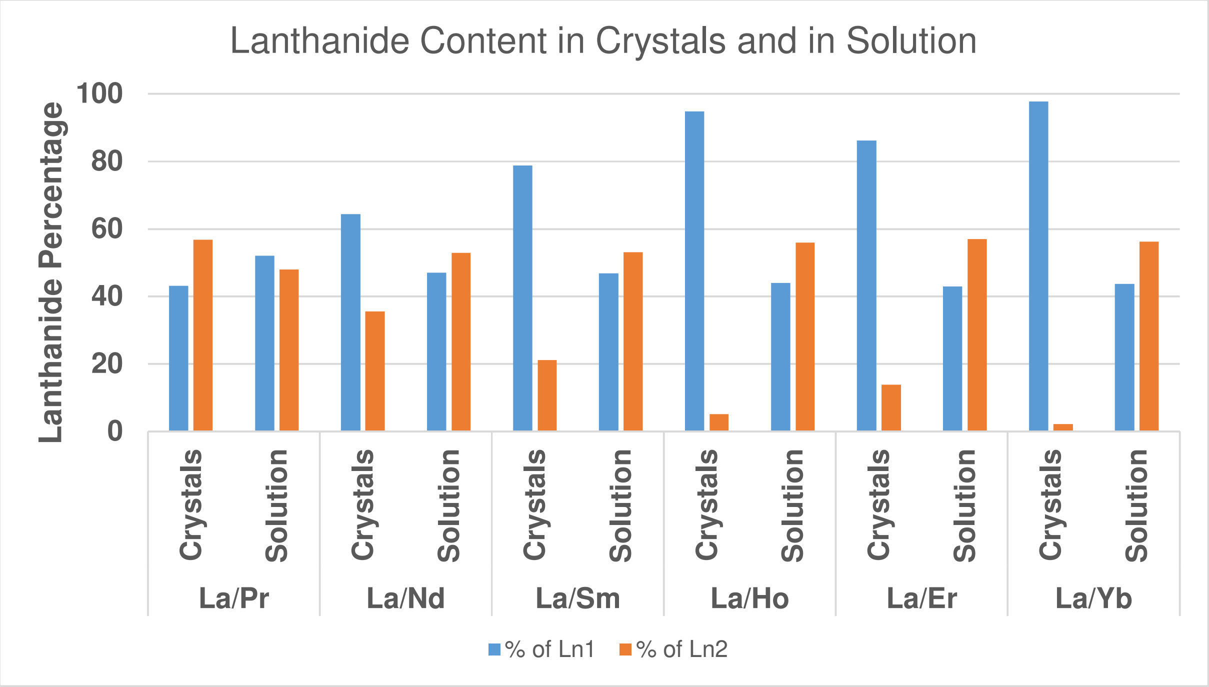

Figure S1. Comparison of the lanthanide composition between the different states (in the isolated crystals and in solution) of all the binary mixtures attempted at a 1:1 ratio.

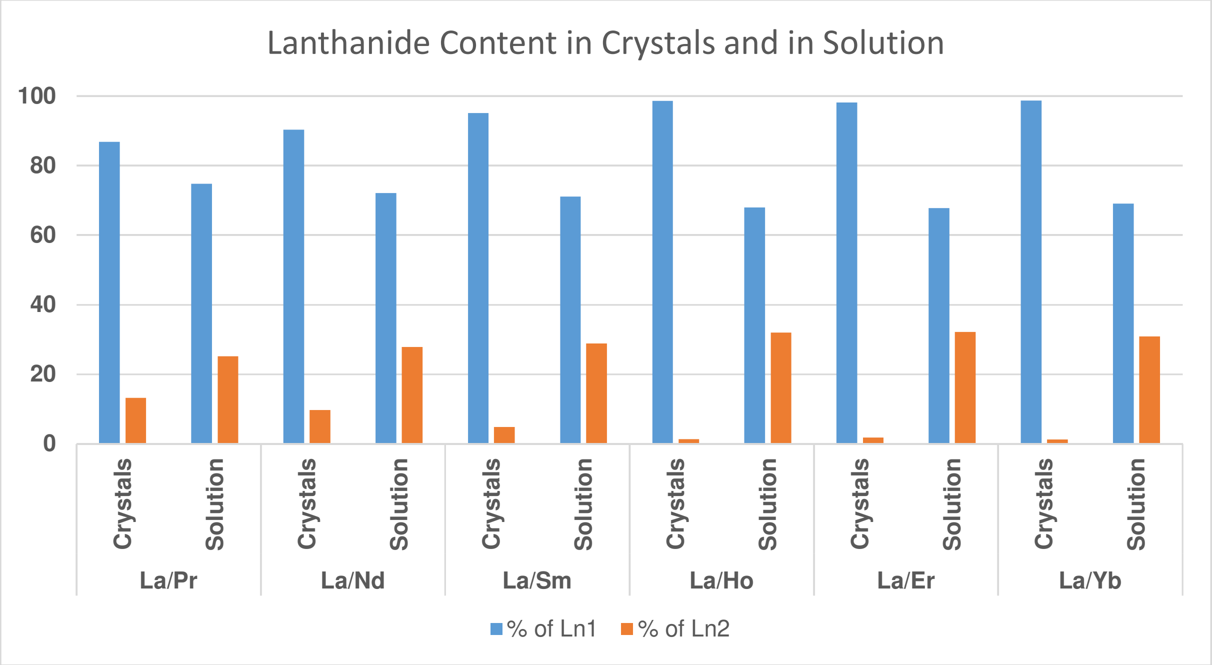

Figure S2. Comparison of the lanthanide composition between the different states (in the isolated crystals and in solution) of all the binary mixtures attempted at a 3:1 ratio.

5. Crystallographic data

Table S1. Brief summary of crystal data for comparison of lattices, unit cells and volumes per cluster†

Compounds	Struct type	Crystal Lattice	Space Group	Z	Unit cell parameters					Vol per cluster (Å^3)
					a (Å)	b (Å)	c (Å)	β (º)	Vol (Å^3)	
**1** {$Mo_{64}Ni_8La_6$}	cube	Tetragonal	*I*4/*m*	2	24.421	24.421	22.838	90	13620	6810
**2** {$Mo_{64}Ni_8Ce_6$}	cube	Tetragonal	*I*4/*m*	2	24.394	24.394	22.808	90	13572	6786
**2'** {$Mo_{64}Ni_8Ce_6$}	cube	Tetragonal	*I*4/*m*	2	25.725	25.725	18.949	90	12540	6270
**3** {$Mo_{64}Ni_8Pr_6$}	cube	Tetragonal	*I*4/*m*	2	25.544	25.544	18.813	90	12275	6138
**4** {$Mo_{70}Ce_{4.5}$}	star	Monoclinic	C2/c	8	60.791	26.806	50.418	91.678	82125	10266
**5** {$Mo_{70}Nd_5$}	star	Monoclinic	$P2_1/c$	4	31.579	27.739	40.667	110.14	33492	8373
**6** {$Mo_{70}Sm_5$}	star	Monoclinic	$P2_1/n$	4	20.856	26.542	73.429	95.396	40264	10066

† Volume per cluster (VPC) is defined by the volume that each cluster with associated cation and anion and solvent molecules averagely occupies in solid state single crystal structure. It is calculated from the unit cell volume divided by number of the clusters (Z) in the unit cell.

Compound **1**, **2**, **2'** and **3** all have the cube-type structures of similar formula but with different number of solvent water molecules and crystallise in the two significantly different unit cells. **1** and **2** are isomorphous while **2'** and **3** are isomorphous. Their formulae were determined jointly by structure refinements and chemical analyses. At the centre of cube structures, there are electron densities much higher than normal contributions from disordered solvent waters and shaped as small polyhedrons. Due to high symmetry and heavy disorder, no clear molecule was modelled, but heavily disordered ClO_4^- anions were proposed and assigned to the formulae based on best evaluation of all possibilities. A tetrahedral MoO_4^{2-} as template at the cube centre is secondly possible but no further evidence yet to verify. The number of protons on clusters were roughly determined from BVS calculated for oxo ligand protonation and the charge balances required. The number of solvent water molecules were estimated from thermal analyses, SQUEEZE and the calculation of volume per non-H atom. The tetrahedral Mo centres on the cube edge centres have 1/3 occupancy found in structure refinements with occupancies refined first and then fixed. We proposed lowest energy and most symmetric state for 4 Mo centres to locate on furthest separated four edge centres of the twelve cube edges.

Compounds **4**, **5** and **6** all have star-shaped structures but crystallise in three different lattice systems. Their formulae were mainly determined by crystallography. The contents of Ni, lanthanide, Cl and Mo were found in structure refinements with occupancies refined freely first and then fixed. Number of protons on clusters were roughly determined from BVS calculated for oxo ligand protonation for compounds **4** and **5**. For compound **6**, due to poor crystal diffraction data, no BVS calculation was performed but the number of protons was adopted from compound **4** and **5** based on structure similarity. Na contents are estimated for final charge balances. Three star-type compounds all contain large solvent area and yield crystals with heavy disorders around the solvent areas and crystal packing. Low crystallinity and weak diffraction in high angle ranges result in poor crystal data, which show high R(int), wR2 and R1 values. Nevertheless, the connectivities of Mo and lanthanide centres with associated oxo or water ligands in the clusters are clearly shown, good enough for element recognitions and definitions. The clusters were reasonably modelled and refined. In compound **6**, due to poor data, a few Mo-O bonds are not well defined, for example some Mo=O terminals showing bond distances as short as 1.63 Å.

SQUEEZE (from PLATON) was used to calculate the void space, the electron counts for the disordered solvent areas and to produce new HKL files for further refinements for all structures. SQUEEZE procedures improved the quality of all structures significantly. CCDC 2105014-2105020 contain the supplementary crystallographic data and can be obtained free of charge via www.ccdc.cam.ac.uk./data_request/cif.

Table S2. Crystal data and structure refinement details for compounds **1** and **2**.

Compound code	**1**	**2**
Empirical formula	$ClH_{196}La_6Mo_{64}Na_9Ni_8O_{289}$	$Ce_6ClH_{196}Mo_{64}Na_9Ni_8O_{289}$
Formula weight	12507.22	12514.48
Temperature (K)	150(2)	150(2)
Crystal system	Tetragonal	Tetragonal
Space group	$I4/m$	$I4/m$
a (Å)	24.4208(2)	24.3944(3)
b (Å)	24.4208(2)	24.3944(3)
c (Å)	22.8385(2)	22.8076(5)
Volume (Å^3)	13620.3(3)	13572.5(4)
Z	2	2
Density calculated (Mg/m^3)	3.050	3.062
Absorption coefficient (mm^{-1})	4.437	4.515
F(000)	11756	11768
θ range for data collection	2.359 to 25.999°.	2.445 to 25.999°.
Index ranges	$-30<=h<=30$,<br>$-30<=k<=30$,<br>$-28<=l<=28$	-30<=h<=27,<br>-29<=k<=29,<br>-28<=l<=21
Reflections collected	102240	11762
Independent reflections	6883 [R(int) = 0.0166]	6385 [R(int) = 0.0202]
Completeness (θ = 25.242)	99.8 %	93.4 %
Data/restraints/parameters	6883 / 0 / 422	6385 / 0 / 424
Goodness-of-fit on F^2	1.085	1.073
Final R indices [I>2σ(I)]	$R1$ = 0.0243, w$R2$ = 0.0709	$R1$ = 0.0271, w$R2$ = 0.0736
R indices (all data)	$R1$ = 0.0251, w$R2$ = 0.0713	$R1$ = 0.0346, w$R2$ = 0.0763
Max/min Δρ (e Å^{-3})	1.83 and -1.16	1.08 and -0.62

Table S3. Crystal data and structure refinement details for compounds **2’** and **3**.

Compound code	**2’**	**3**
Empirical formula	$Ce_6ClH_{146}Mo_{64}Na_9Ni_8O_{264}$	$ClH_{146}Mo_{64}Na_9Ni_8O_{264}Pr_6$
Formula weight	12064.08	12068.82
Temperature (K)	150(2)	150(2)
Crystal system	Tetragonal	Tetragonal
Space group	*I*4/*m*	*I*4/*m*
a (Å)	25.7249(2)	25.5441(2)
b (Å)	25.7249(2)	25.5441(2)
c (Å)	18.9493(2)	18.8128(2)
Volume ($Å^3$)	12540.1(2)	12275.4(2)
Z	2	2
Density calculated (Mg/m^3)	3.195	3.265
Absorption coefficient (mm^{-1})	4.873	5.057
F(000)	11268	11280
θ range for data collection	2.239 to 25.990°.	2.441 to 25.992°.
Index ranges	-31<=*h*<=31,<br>-31<=*k*<=31,<br>-23<=*l*<=23	-31<=*h*<=31,<br>-31<=*k*<=31,<br>-22<=*l*<=23
Reflections collected	80998	94134
Independent reflections	6357 [*R*(int) = 0.0363]	6221 [*R*(int) = 0.0257]
Completeness (θ = 25.242)	99.8 %	99.8 %
Data/restraints/parameters	6357 / 0 / 420	6221 / 2 / 421
Goodness-of-fit on F^2	1.042	1.049
Final *R* indices [$I>2\sigma(I)$]	*R*1 = 0.0280, w*R*2 = 0.0766	*R*1 = 0.0276, w*R*2 = 0.0772
R indices (all data)	*R*1 = 0.0326, w*R*2 = 0.0789	*R*1 = 0.0301, w*R*2 = 0.0784
Max/min $\Delta\rho$ (e $Å^{-3}$)	1.903 and -1.259	2.41 and -1.13 e.$Å^{-3}$

Table S4. Crystal data and structure refinement details for compounds **4, 5** and **6**.

Compound code	**4**	**5**	**6**
Empirical formula	$Ce_{5.5}Cl_{0.5}H_{530}Mo_{70}Na_3Ni_{0.5}O_{465}$	$H_{390}Mo_{70}Na_3Nd_{5.67}O_{395}$	$H_{530}Mo_{70}Na_{3.5}O_{465}Sm_{5.5}$
Formula weight	15576.72	14315.24	15597.40
Temperature (K)	150(2)	150(2)	150(2)
Crystal system	Monoclinic	Monoclinic	Monoclinic
Space group	*C*2/*c*	$P2_1/c$	$P2_1/n$
a (Å)	60.7906(7)	31.5796(7)	20.8564(2)
b (Å)	26.8065(2)	27.7392(4)	26.5422(4)
c (Å)	50.4181(6)	40.6670(8)	73.4293(9)
β (°)	91.678(2)°	110.138(2)°	95.396(2)°
Volume (Å^3)	82125.2(15)	33446.2(12)	40468.5(9)
Z	8	4	4
Density calculated (Mg/m^3)	2.520	2.843	2.560
Absorption coefficient (mm^{-1})	2.820	3.533	3.015
F(000)	60516	27452	30278
θ range for data collection	2.230 to 26.000°.	2.227 to 26.000°.	2.235 to 25.726°.
Index ranges	-74<=*h*<=74, -32<=*k*<=31, -62<=*l*<=62	-38<=*h*<=38, -34<=*k*<=34, -50<=*l*<=50	-25<=*h*<=25, -31<=*k*<=32, -89<=*l*<=89
Reflections collected	882345	680815	659049
Independent reflections	80376 [*R*(int) = 0.3420]	65667 [*R*(int) = 0.3755]	77389 [*R*(int) = 0.2376]
Completeness (θ = 25.242)	99.7 %	99.9 %	99.9 %
Data/restraints/parameters	80376 / 92 / 3067	65667 / 96 / 2964	77389 / 1458 / 2914
Goodness-of-fit on F^2	0.987	0.990	1.031
Final *R* indices [I>2σ(I)]	*R*1 = 0.0728, w*R*2 = 0.1504	*R*1 = 0.0767, w*R*2 = 0.1661	*R*1 = 0.1455, w*R*2 = 0.3522
R indices (all data)	*R*1 = 0.1474, w*R*2 = 0.1790	*R*1 = 0.1860, w*R*2 = 0.2142	*R*1 = 0.1746, w*R*2 = 0.3704
Max/min Δρ (e Å^{-3})	1.72 and -1.10	2.61 and -1.73	3.42 and -3.03

6. Bond valence sum (BVS) studies

Bond valence calculations were performed for each bond using the bond distance (R) measured and empirical parameters R0 and B: bond valence = EXP((R0-R)/B). BVS for each metal centre was then summed from all bond valences of the bonds listed. The parameters R0 and B were taken from Gagne & Hawthorne.[8] Mo(6+) and Mo(5+) centres were calculated separately using corresponding parameters listed below. For compound **6**, due to poor crystal diffraction data, no BVS calculation was performed.

Parameter list:

Bond	R0	B
Mo(6+)-O	1.903	0.349
Mo(5+)-O	1.888	0.314
La(3+)-O	2.179	0.359
Ce(3+)-O	2.114	0.389
Pr(3+)-O	2.071	0.411
Nd(3+)-O	2.103	0.371
Sm(3+)-O	2.049	0.404
Ni(2+)-O	1.689	0.347

Detailed bond valence sum calculations for each compound:

Compound 1

Bond		R	BVS	Bond		R	BVS	Bond		R	BVS	Bond		R	BVS	Bond		R	BVS
Mo1	O2	1.689	1.885	Mo2	O6	1.687	1.897	Mo3	O12	1.685	1.909	Mo4	O15	1.686	1.903	La1	O30	2.484	0.428
Mo1	O4	1.953	0.813	Mo2	O4	1.949	0.823	Mo3	O13	1.953	0.813	Mo4	O13	1.953	0.813	La1	O22	2.508	0.400
Mo1	O5	1.957	0.803	Mo2	O5	1.958	0.800	Mo3	O14	1.959	0.798	Mo4	O14	1.957	0.803	La1	O25	2.512	0.396
Mo1	O1	2.017	0.663	Mo2	O7	2.013	0.672	Mo3	O9	2.016	0.665	Mo4	O1	2.016	0.665	La1	O25	2.512	0.396
Mo1	O3	2.134	0.457	Mo2	O11	2.129	0.464	Mo3	O11	2.132	0.460	Mo4	O29	2.134	0.457	La1	O10	2.512	0.396
Mo1	O23	2.294	0.274	Mo2	O8	2.306	0.264	Mo3	O8	2.303	0.267	Mo4	O16	2.304	0.266	La1	O14	2.649	0.270
			4.895				4.920				4.911				4.906	La1	O14	2.649	0.270
Mo5	O17	1.692	1.867	Mo6	O20	1.694	1.855	Mo7	O22	1.725	1.665	Mo8	O25	1.723	1.675	La1	O5	2.668	0.256
Mo5	O19	1.954	0.810	Mo6	O19	1.948	0.826	Mo7	O10	1.731	1.637	Mo8	O24	1.728	1.651	La1	O5	2.668	0.256
Mo5	O18	1.956	0.805	Mo6	O18	1.954	0.810	Mo7	O8	1.949	0.877	Mo8	O16	1.931	0.923				3.066
Mo5	O21	2.016	0.665	Mo6	O21	2.016	0.665	Mo7	O8	1.946	0.884	Mo8	O23	1.949	0.877	La2	O31	2.484	0.428
Mo5	O29	2.138	0.451	Mo6	O3	2.130	0.463	Mo7	O9	2.145	0.500	Mo8	O1	2.162	0.476	La2	O24	2.509	0.399
Mo5	O16	2.284	0.283	Mo6	O23	2.300	0.269	Mo7	O7	2.171	0.464	Mo8	O21	2.168	0.468	La2	O24	2.509	0.399
			4.882				4.889				6.027				6.070	La2	O24	2.509	0.399
Mo9	O33	1.703	1.802	Mo10	O34	1.699	1.826	Ni1	O19	2.035	0.369					La2	O24	2.509	0.399
Mo9	O32	1.704	1.797	Mo10	O45	1.709	1.768	Ni1	O13	2.039	0.365					La2	O18	2.665	0.258
Mo9	O11	2.044	0.608	Mo10	O29	2.039	0.618	Ni1	O4	2.043	0.361					La2	O18	2.665	0.258
Mo9	O11	2.044	0.608	Mo10	O3	2.040	0.616	Ni1	O26	2.056	0.347					La2	O18	2.665	0.258
			4.816				4.828	Ni1	O27	2.077	0.327					La2	O18	2.665	0.258
								Ni1	O28	2.084	0.320								3.056
											2.089								

Compound 2

Bond		R	BVS	Bond		R	BVS	Bond		R	BVS	Bond		R	BVS	Bond		R	BVS
Mo1	O2	1.688	1.891	Mo2	O6	1.689	1.885	Mo3	O12	1.683	1.921	Mo4	O15	1.687	1.897	Ce1	O30	2.454	0.417
Mo1	O4	1.949	0.823	Mo2	O4	1.951	0.818	Mo3	O13	1.952	0.816	Mo4	O13	1.949	0.823	Ce1	O22	2.482	0.388
Mo1	O5	1.958	0.800	Mo2	O5	1.960	0.795	Mo3	O14	1.956	0.805	Mo4	O14	1.954	0.810	Ce1	O25	2.489	0.381
Mo1	O1	2.014	0.669	Mo2	O7	2.010	0.678	Mo3	O9	2.015	0.667	Mo4	O1	2.008	0.682	Ce1	O25	2.500	0.371
Mo1	O3	2.136	0.454	Mo2	O11	2.129	0.464	Mo3	O11	2.134	0.457	Mo4	O29	2.136	0.454	Ce1	O10	2.500	0.371
Mo1	O23	2.297	0.272	Mo2	O8	2.307	0.263	Mo3	O8	2.300	0.269	Mo4	O16	2.304	0.266	Ce1	O14	2.646	0.255
			4.910				4.904				4.935				4.933	Ce1	O14	2.646	0.255
Mo5	O17	1.683	1.921	Mo6	O20	1.688	1.891	Mo7	O22	1.729	1.646	Mo8	O25	1.718	1.699	Ce1	O5	2.662	0.244
Mo5	O19	1.953	0.813	Mo6	O19	1.947	0.829	Mo7	O10	1.729	1.646	Mo8	O24	1.729	1.646	Ce1	O5	2.662	0.244
Mo5	O18	1.956	0.805	Mo6	O18	1.948	0.826	Mo7	O8	1.943	0.892	Mo8	O16	1.935	0.912				2.927
Mo5	O21	2.018	0.661	Mo6	O21	2.005	0.689	Mo7	O8	1.943	0.892	Mo8	O23	1.952	0.869	Ce2	O31	2.451	0.420
Mo5	O29	2.139	0.450	Mo6	O3	2.130	0.463	Mo7	O9	2.148	0.496	Mo8	O1	2.162	0.476	Ce2	O24	2.487	0.383
Mo5	O16	2.280	0.287	Mo6	O23	2.304	0.266	Mo7	O7	2.171	0.464	Mo8	O21	2.168	0.468	Ce2	O24	2.487	0.383
			4.937				4.963				6.036				6.071	Ce2	O24	2.487	0.383
Mo9	O33	1.673	1.983	Mo10	O34	1.673	1.983	Ni1	O19	2.038	0.366					Ce2	O24	2.487	0.383
Mo9	O32	1.705	1.791	Mo10	O45	1.695	1.849	Ni1	O13	2.040	0.364					Ce2	O18	2.664	0.243
Mo9	O11	2.030	0.636	Mo10	O29	2.032	0.632	Ni1	O4	2.041	0.363					Ce2	O18	2.664	0.243
Mo9	O11	2.030	0.636	Mo10	O3	2.052	0.593	Ni1	O26	2.062	0.341					Ce2	O18	2.664	0.243
			5.047				5.058	Ni1	O27	2.092	0.313					Ce2	O18	2.664	0.243
								Ni1	O28	2.110	0.297								2.927
											2.044								

Compound 2'

Bond		R	BVS	Bond		R	BVS	Bond		R	BVS	Bond		R	BVS	Bond		R	BVS
Mo1	O2	1.683	1.921	Mo2	O3	1.682	1.927	Mo3	O9	1.682	1.927	Mo4	O11	1.686	1.903	Ce1	O34	2.456	0.415
Mo1	O1	1.956	0.805	Mo2	O4	1.951	0.818	Mo3	O8	1.955	0.808	Mo4	O8	1.952	0.816	Ce1	O18	2.472	0.398
Mo1	O4	1.954	0.810	Mo2	O1	1.960	0.795	Mo3	O10	1.964	0.785	Mo4	O10	1.960	0.795	Ce1	O18	2.472	0.398
Mo1	O7	2.012	0.674	Mo2	O7	2.005	0.689	Mo3	O15	2.016	0.665	Mo4	O12	2.010	0.678	Ce1	O18	2.472	0.398
Mo1	O22	2.123	0.473	Mo2	O5	2.123	0.473	Mo3	O26	2.129	0.464	Mo4	O5	2.118	0.481	Ce1	O18	2.472	0.398
Mo1	O19	2.308	0.262	Mo2	O6	2.302	0.268	Mo3	O14	2.297	0.272	Mo4	O6	2.311	0.260	Ce1	O1	2.663	0.244
			4.946				4.970				4.921				4.932	Ce1	O1	2.663	0.244
Mo5	O13	1.727	1.656	Mo6	O17	1.726	1.661	Mo7	O20	1.685	1.868	Mo8	O25	1.680	1.939	Ce1	O1	2.663	0.244
Mo5	O16	1.730	1.642	Mo6	O18	1.731	1.637	Mo7	O23	1.945	0.887	Mo8	O23	1.954	0.810	Ce1	O1	2.663	0.244
Mo5	O14	1.938	0.905	Mo6	O19	1.937	0.907	Mo7	O21	1.953	0.867	Mo8	O21	1.964	0.785				2.984
Mo5	O14	1.938	0.905	Mo6	O6	1.943	0.892	Mo7	O12	2.015	0.725	Mo8	O24	2.018	0.661	Ce2	O17	2.483	0.387
Mo5	O15	2.167	0.469	Mo6	O7	2.163	0.475	Mo7	O22	2.131	0.520	Mo8	O26	2.129	0.464	Ce2	O17	2.483	0.387
Mo5	O24	2.167	0.469	Mo6	O12	2.164	0.473	Mo7	O19	2.284	0.336	Mo8	O14	2.311	0.260	Ce2	O13	2.507	0.364
			6.045				6.045				5.202				4.920	Ce2	O16	2.511	0.360
Mo9	O29	1.685	1.909	Mo10	O27	1.678	1.952	Ni1	O4	2.025	0.380					Ce2	O35	2.634	0.263
Mo9	O30	1.695	1.849	Mo10	O28	1.684	1.915	Ni1	O8	2.040	0.364					Ce2	O21	2.655	0.249
Mo9	O22	2.010	0.678	Mo10	O26	2.035	0.626	Ni1	O23	2.046	0.357					Ce2	O21	2.655	0.249
Mo9	O5	2.031	0.634	Mo10	O26	2.035	0.626	Ni1	O33	2.083	0.321					Ce2	O10	2.660	0.246
			5.070				5.119	Ni1	O32	2.089	0.316					Ce2	O10	2.660	0.246
								Ni1	O31	2.092	0.313								2.751
											2.051								

Comound 3

Bond		R	BVS	Bond		R	BVS	Bond		R	BVS	Bond		R	BVS	Bond		R	BVS
Mo1	O2	1.683	1.921	Mo2	O5	1.689	1.885	Mo3	O7	1.681	1.933	Mo4	O10	1.681	1.933	Pr1	O25	2.442	0.405
Mo1	O3	1.952	0.816	Mo2	O3	1.954	0.810	Mo3	O9	1.953	0.813	Mo4	O9	1.949	0.823	Pr1	O25	2.442	0.405
Mo1	O4	1.961	0.793	Mo2	O4	1.961	0.793	Mo3	O8	1.957	0.803	Mo4	O8	1.960	0.795	Pr1	O30	2.481	0.369
Mo1	O1	2.017	0.663	Mo2	O13	2.008	0.682	Mo3	O11	2.001	0.698	Mo4	O11	2.002	0.696	Pr1	O21	2.490	0.361
Mo1	O22	2.139	0.450	Mo2	O27	2.126	0.469	Mo3	O27	2.117	0.482	Mo4	O12	2.125	0.470	Pr1	O20	2.491	0.360
Mo1	O18	2.296	0.273	Mo2	O6	2.290	0.278	Mo3	O6	2.317	0.255	Mo4	O26	2.309	0.262	Pr1	O4	2.657	0.240
			4.915				4.917				4.984				4.979	Pr1	O4	2.657	0.240
Mo5	O14	1.691	1.873	Mo6	O17	1.688	1.891	Mo7	O25	1.729	1.646	Mo8	O20	1.732	1.632	Pr1	O16	2.663	0.237
Mo5	O16	1.961	0.793	Mo6	O15	1.956	0.805	Mo7	O24	1.733	1.628	Mo8	O21	1.733	1.628	Pr1	O16	2.663	0.237
Mo5	O15	1.962	0.790	Mo6	O16	1.959	0.798	Mo7	O6	1.944	0.889	Mo8	O18	1.932	0.920				2.855
Mo5	O13	2.009	0.680	Mo6	O19	2.018	0.661	Mo7	O26	1.947	0.882	Mo8	O18	1.932	0.920	Pr2	O31	2.411	0.437
Mo5	O12	2.117	0.482	Mo6	O22	2.133	0.458	Mo7	O11	2.155	0.486	Mo8	O19	2.186	0.444	Pr2	O24	2.458	0.390
Mo5	O26	2.314	0.258	Mo6	O18	2.284	0.283	Mo7	O13	2.161	0.477	Mo8	O1	2.198	0.429	Pr2	O24	2.458	0.390
			4.875				4.896				6.008				5.974	Pr2	O24	2.458	0.390
Mo9	O33	1.675	1.971	Mo10	O32	1.652	2.120	Ni1	O9	2.021	0.384					Pr2	O24	2.458	0.390
Mo9	O35	1.709	1.768	Mo10	O36	1.681	1.933	Ni1	O3	2.034	0.370					Pr2	O8	2.647	0.246
Mo9	O22	2.023	0.651	Mo10	O27	2.025	0.646	Ni1	O15	2.038	0.366					Pr2	O8	2.647	0.246
Mo9	O22	2.023	0.651	Mo10	O12	2.027	0.642	Ni1	O29	2.092	0.313					Pr2	O8	2.647	0.246
Mo9	O19	2.467	0.158				5.342	Ni1	O23	2.093	0.312					Pr2	O8	2.647	0.246
Mo9	O1	2.509	0.138					Ni1	O28	2.095	0.310								2.982
			5.337								2.055								

Compound 4

Bond		R	BVS	Bond		R	BVS	Bond		R	BVS	Bond		R	BVS	Bond		R	BVS
Mo1	O137	1.710	1.763	Mo2	O144	1.710	1.738	Mo3	O199	1.699	1.794	Mo4	O211	1.720	1.689	Mo5	O100	1.712	1.729
Mo1	O73	1.937	0.856	Mo2	O196	1.720	1.689	Mo3	O104	1.734	1.623	Mo4	O210	1.723	1.675	Mo5	O208	1.717	1.704
Mo1	O214	1.975	0.758	Mo2	O46	1.863	1.121	Mo3	O94	1.860	1.131	Mo4	O57	1.803	1.332	Mo5	O92	1.800	1.343
Mo1	O61	2.069	0.562	Mo2	O198	1.958	0.854	Mo3	O198	1.947	0.882	Mo4	O48	2.190	0.439	Mo5	O114	2.203	0.423
Mo1	O192	2.090	0.526	Mo2	O92	2.294	0.326	Mo3	O62	2.306	0.315	Mo4	O213	2.202	0.425	Mo5	O183	2.205	0.421
Mo1	O67	2.132	0.460	Mo2	O77	2.353	0.275	Mo3	O92	2.307	0.314	Mo4	O32	2.274	0.345	Mo5	O158	2.222	0.401
			4.923				6.005				6.059				5.905				6.021
Mo6	O112	1.723	1.675	Mo7	O71	1.722	1.680	Mo8	O202	1.647	2.154	Mo9	O148	1.662	2.054	Mo10	O86	1.703	1.802
Mo6	O35	1.790	1.382	Mo7	O60	1.794	1.367	Mo8	O22	1.963	0.788	Mo9	O43	1.984	0.737	Mo10	O78	1.970	0.770
Mo6	O63	1.799	1.347	Mo7	O33	1.807	1.317	Mo8	O36	1.986	0.732	Mo9	O156	1.996	0.709	Mo10	O183	1.978	0.751
Mo6	O12	2.012	0.732	Mo7	O3	2.022	0.711	Mo8	O74	2.029	0.638	Mo9	O157	2.081	0.541	Mo10	O11	2.049	0.599
Mo6	O3	2.042	0.671	Mo7	O12	2.034	0.687	Mo8	O47	2.032	0.632	Mo9	O36	2.085	0.534	Mo10	O172	2.127	0.467
Mo6	O186	2.332	0.293	Mo7	O34	2.404	0.238	Mo8	O52	2.219	0.348	Mo9	O159	2.228	0.339	Mo10	O29	2.138	0.451
			6.100				5.999				5.293				4.913				4.840
Mo11	O107	1.694	1.855	Mo12	O194	1.681	1.933	Mo13	O123	1.729	1.646	Mo14	O80	1.714	1.719	Mo15	O193	1.698	1.831
Mo11	O13	1.973	0.763	Mo12	O5	1.977	0.753	Mo13	O84	1.732	1.632	Mo14	O168	1.806	1.320	Mo15	O65	1.961	0.793
Mo11	O158	1.986	0.732	Mo12	O166	1.983	0.739	Mo13	O103	1.832	1.226	Mo14	O81	1.811	1.302	Mo15	O64	1.973	0.763
Mo11	O163	1.999	0.702	Mo12	O187	2.075	0.551	Mo13	O49	1.954	0.864	Mo14	O165	2.033	0.689	Mo15	O30	2.090	0.526
Mo11	O46	2.014	0.669	Mo12	O192	2.082	0.539	Mo13	O21	2.248	0.372	Mo14	O25	2.066	0.627	Mo15	O22	2.103	0.504
Mo11	O77	2.279	0.288	Mo12	O61	2.103	0.504	Mo13	O95	2.292	0.328	Mo14	O38	2.341	0.285	Mo15	O7	2.203	0.367
			5.009				5.020				6.068				5.942				4.783
Mo16	O120	1.667	2.021	Mo17	O128	1.661	2.060	Mo18	O132	1.675	1.971	Mo19	O96	1.670	2.002	Mo20	O154	1.696	1.810
Mo16	O32	1.962	0.790	Mo17	O18	1.972	0.765	Mo18	O157	1.988	0.727	Mo19	O26	1.977	0.753	Mo20	O93	1.713	1.724
Mo16	O124	2.007	0.685	Mo17	O178	1.976	0.756	Mo18	O41	2.000	0.700	Mo19	O10	1.996	0.709	Mo20	O179	1.854	1.151
Mo16	O70	2.026	0.644	Mo17	O176	2.073	0.555	Mo18	O53	2.005	0.689	Mo19	O35	2.020	0.657	Mo20	O177	1.969	0.828
Mo16	O185	2.027	0.642	Mo17	O169	2.080	0.543	Mo18	O72	2.018	0.661	Mo19	O103	2.035	0.626	Mo20	O174	2.281	0.339
Mo16	O66	2.257	0.309	Mo17	O87	2.092	0.522	Mo18	O39	2.288	0.280	Mo19	O21	2.289	0.279	Mo20	O131	2.304	0.317
			5.092				5.201				5.028				5.026				6.167
Mo21	O151	1.706	1.759	Mo22	O117	1.702	1.779	Mo23	O155	1.705	1.764								
Mo21	O138	1.720	1.689	Mo22	O209	1.728	1.651	Mo23	O133	1.743	1.582								
Mo21	O51	1.807	1.317	Mo22	O174	1.811	1.302	Mo23	O97	1.840	1.198	Mo24	O162	1.734	1.623	Mo25	O69	1.715	1.714
Mo21	O41	2.181	0.451	Mo22	O44	2.184	0.447	Mo23	O91	1.971	0.823	Mo24	O83	1.744	1.577	Mo25	O125	1.739	1.600
Mo21	O23	2.207	0.419	Mo22	O207	2.184	0.447	Mo23	O51	2.282	0.338	Mo24	O1	1.804	1.328	Mo25	O21	1.837	1.208
Mo21	O191	2.210	0.415	Mo22	O73	2.263	0.356	Mo23	O68	2.320	0.303	Mo24	O66	1.822	1.261	Mo25	O19	1.844	1.184
			6.049				5.982				6.006				5.789				5.706
Mo26	O108	1.690	1.879	Mo27	O153	1.668	2.015	Mo28	O116	1.699	1.826	Mo29	O88	1.708	1.748	Mo30	O150	1.671	1.996
Mo26	O9	1.958	0.800	Mo27	O64	1.970	0.770	Mo28	O160	1.973	0.763	Mo29	O54	1.805	1.324	Mo30	O34	1.986	0.732
Mo26	O2	1.976	0.756	Mo27	O65	1.983	0.739	Mo28	O75	1.981	0.744	Mo29	O16	1.811	1.302	Mo30	O186	1.990	0.723
Mo26	O161	2.067	0.565	Mo27	O166	2.074	0.553	Mo28	O167	2.076	0.550	Mo29	O28	2.021	0.713	Mo30	O20	2.063	0.573
Mo26	O11	2.081	0.541	Mo27	O214	2.094	0.519	Mo28	O8	2.094	0.519	Mo29	O4	2.026	0.703	Mo30	O10	2.079	0.544
Mo26	O172	2.108	0.496	Mo27	O15	2.232	0.334	Mo28	O28	2.243	0.323	Mo29	O160	2.394	0.245	Mo30	O12	2.224	0.343
			5.037				4.930				4.723				6.035				4.910
Mo31	O111	1.678	1.952	Mo32	O147	1.663	2.047	Mo33	O118	1.715	1.714	Mo34	O188	1.691	1.836	Mo35	O115	1.730	1.642
Mo31	O160	1.980	0.746	Mo32	O45	1.978	0.751	Mo33	O31	1.794	1.367	Mo34	O85	1.799	1.347	Mo35	O206	1.745	1.573
Mo31	O75	2.004	0.691	Mo32	O38	1.986	0.732	Mo33	O163	1.823	1.258	Mo34	O42	1.817	1.279	Mo35	O175	1.829	1.236
Mo31	O2	2.061	0.576	Mo32	O17	2.089	0.527	Mo33	O4	2.036	0.683	Mo34	O25	2.023	0.709	Mo35	O49	1.941	0.897
Mo31	O13	2.085	0.534	Mo32	O5	2.090	0.526	Mo33	O28	2.057	0.643	Mo34	O165	2.031	0.693	Mo35	O95	2.269	0.350
Mo31	O4	2.232	0.334	Mo32	O165	2.255	0.311	Mo33	O75	2.327	0.297	Mo34	O45	2.397	0.243	Mo35	O19	2.304	0.317
			4.834				4.894				5.961				6.107				6.015
Mo36	O76	1.658	2.080	Mo37	O135	1.668	2.015	Mo38	O89	1.674	1.977	Mo39	O215	1.668	2.015	Mo40	O130	1.703	1.802
Mo36	O5	1.988	0.727	Mo37	O186	1.965	0.783	Mo38	O2	2.007	0.685	Mo39	O178	1.996	0.709	Mo40	O43	1.969	0.773
Mo36	O166	1.988	0.727	Mo37	O34	1.970	0.770	Mo38	O9	2.015	0.667	Mo39	O18	1.998	0.704	Mo40	O156	1.988	0.727
Mo36	O85	2.017	0.663	Mo37	O178	2.072	0.557	Mo38	O42	2.018	0.661	Mo39	O33	2.006	0.687	Mo40	O18	2.076	0.550
Mo36	O50	2.020	0.657	Mo37	O170	2.083	0.537	Mo38	O16	2.025	0.646	Mo39	O171	2.016	0.665	Mo40	O124	2.090	0.526
Mo36	O182	2.241	0.325	Mo37	O3	2.194	0.377	Mo38	O164	2.188	0.385	Mo39	O162	2.193	0.379	Mo40	O14	2.205	0.364
			5.180				5.039				5.021				5.159				4.742

Compound 4 (continued)

Mo41	O55	1.729	1.646	Mo42	O149	1.710	1.763	Mo43	O126	1.714	1.719	Mo44	O204	1.694	1.855				
Mo41	O185	1.795	1.363	Mo42	O167	1.976	0.756	Mo43	O74	1.808	1.313	Mo44	O78	1.985	0.734				
Mo41	O53	1.813	1.294	Mo42	O173	1.987	0.730	Mo43	O171	1.816	1.283	Mo44	O183	1.986	0.732	Mo45	O119	1.725	1.665
Mo41	O14	2.045	0.666	Mo42	O31	2.009	0.680	Mo43	O159	2.025	0.705	Mo44	O168	1.996	0.709	Mo45	O164	1.745	1.573
Mo41	O159	2.052	0.653	Mo42	O175	2.047	0.603	Mo43	O14	2.036	0.683	Mo44	O94	2.001	0.698	Mo45	O77	1.805	1.324
Mo41	O156	2.305	0.316	Mo42	O19	2.251	0.315	Mo43	O43	2.383	0.253	Mo44	O62	2.283	0.284	Mo45	O62	1.829	1.236
			5.938				4.846				5.955				5.012				5.798
				Mo47	O189	1.690	1.879	Mo48	O122	1.674	1.977	Mo49	O101	1.707	1.780	Mo50	O152	1.666	2.028
				Mo47	O45	1.968	0.775	Mo48	O8	1.978	0.751	Mo49	O47	1.793	1.353	Mo50	O170	1.990	0.723
Mo46	O102	1.720	1.689	Mo47	O38	1.968	0.775	Mo48	O20	2.006	0.687	Mo49	O50	1.809	1.286	Mo50	O48	2.005	0.689
Mo46	O52	1.726	1.661	Mo47	O9	2.066	0.567	Mo48	O60	2.017	0.663	Mo49	O15	2.042	0.612	Mo50	O63	2.013	0.672
Mo46	O68	1.831	1.229	Mo47	O78	2.089	0.527	Mo48	O54	2.022	0.653	Mo49	O7	2.046	0.605	Mo50	O145	2.015	0.667
Mo46	O39	1.834	1.219	Mo47	O25	2.205	0.364	Mo48	O69	2.230	0.336	Mo49	O64	2.391	0.202	Mo50	O1	2.282	0.285
			5.798				4.888				5.067				5.838				5.064
Mo51	O106	1.687	1.897	Mo52	O113	1.703	1.774	Mo53	O121	1.663	2.047	Mo54	O134	1.687	1.897	Mo55	O200	1.699	1.826
Mo51	O36	1.975	0.758	Mo52	O79	1.787	1.394	Mo53	O10	1.957	0.803	Mo54	O173	1.976	0.756	Mo55	O158	1.964	0.785
Mo51	O22	1.984	0.737	Mo52	O184	1.791	1.378	Mo53	O26	1.964	0.785	Mo54	O167	1.993	0.716	Mo55	O13	1.973	0.763
Mo51	O59	2.048	0.601	Mo52	O15	2.064	0.630	Mo53	O27	2.065	0.569	Mo54	O27	2.048	0.601	Mo55	O11	2.072	0.557
Mo51	O6	2.110	0.493	Mo52	O7	2.076	0.609	Mo53	O181	2.091	0.524	Mo54	O98	2.088	0.529	Mo55	O161	2.081	0.541
Mo51	O37	2.126	0.469	Mo52	O65	2.317	0.305	Mo53	O56	2.137	0.452	Mo54	O56	2.114	0.487	Mo55	O29	2.112	0.490
			4.954				6.091				5.181				4.985				4.961
Mo56	O129	1.652	2.120	Mo57	O109	1.711	1.734	Mo58	O127	1.718	1.699					Mo60	O142	1.701	1.814
Mo56	O17	1.957	0.803	Mo57	O212	1.755	1.528	Mo58	O99	1.723	1.675					Mo60	O48	1.951	0.818
Mo56	O44	1.992	0.718	Mo57	O40	1.838	1.205	Mo58	O145	1.841	1.194	Mo59	O182	1.711	1.734	Mo60	O170	1.966	0.780
Mo56	O81	2.000	0.700	Mo57	O177	1.946	0.884	Mo58	O24	1.952	0.869	Mo59	O105	1.738	1.604	Mo60	O169	2.044	0.608
Mo56	O40	2.018	0.661	Mo57	O180	2.280	0.340	Mo58	O57	2.337	0.288	Mo59	O131	1.821	1.265	Mo60	O87	2.132	0.460
Mo56	O180	2.291	0.277	Mo57	O174	2.290	0.330	Mo58	O1	2.364	0.267	Mo59	O180	1.836	1.212	Mo60	O82	2.154	0.429
			5.279				6.020				5.993				5.814				4.909
Mo61	O203	1.725	1.681	Mo62	O143	1.693	1.861	Mo63	O195	1.663	2.047	Mo64	O190	1.728	1.651	Mo65	O110	1.690	1.879
Mo61	O41	1.971	0.768	Mo62	O30	1.971	0.768	Mo63	O8	1.968	0.775	Mo64	O205	1.735	1.618	Mo65	O73	1.964	0.785
Mo61	O157	1.973	0.763	Mo62	O23	1.997	0.707	Mo63	O20	1.975	0.758	Mo64	O72	1.842	1.191	Mo65	O214	1.978	0.751
Mo61	O6	2.052	0.593	Mo62	O97	2.038	0.620	Mo63	O181	2.079	0.544	Mo64	O91	1.921	0.950	Mo65	O179	2.027	0.642
Mo61	O59	2.083	0.537	Mo62	O184	2.050	0.597	Mo63	O98	2.095	0.517	Mo64	O39	2.269	0.350	Mo65	O79	2.040	0.616
Mo61	O58	2.139	0.450	Mo62	O68	2.272	0.294	Mo63	O27	2.126	0.469	Mo64	O51	2.327	0.297	Mo65	O131	2.298	0.271
			4.791				4.847				5.111				6.057				4.944
Mo66	O197	1.687	1.897	Mo67	O201	1.686	1.903	Mo68	O141	1.716	1.709	Mo69	O136	1.699	1.826	Mo70	O139	1.698	1.799
Mo66	O44	1.973	0.763	Mo67	O32	1.957	0.803	Mo68	O146	1.724	1.670	Mo69	O23	1.978	0.751	Mo70	O140	1.726	1.661
Mo66	O17	1.990	0.723	Mo67	O124	1.970	0.770	Mo68	O70	1.850	1.164	Mo69	O30	1.991	0.720	Mo70	O95	1.820	1.268
Mo66	O187	2.069	0.562	Mo67	O169	2.093	0.521	Mo68	O24	1.918	0.958	Mo69	O6	2.053	0.591	Mo70	O173	2.192	0.437
Mo66	O61	2.077	0.548	Mo67	O176	2.116	0.484	Mo68	O57	2.259	0.361	Mo69	O58	2.119	0.479	Mo70	O90	2.220	0.403
Mo66	O67	2.103	0.504	Mo67	O82	2.129	0.464	Mo68	O66	2.348	0.279	Mo69	O37	2.135	0.455	Mo70	O26	2.227	0.395
			4.996				4.944				6.141				4.823				5.964
Ce1	O112	2.460	0.411	Ce2	O205	2.470	0.400	Ce3	O212	2.457	0.414	Ce4	O234	2.486	0.384	Ce5	O247	2.490	0.380
Ce1	O99	2.501	0.370	Ce2	O104	2.484	0.386	Ce3	O232	2.492	0.378	Ce4	O118	2.514	0.358	Ce5	O246	2.490	0.380
Ce1	O123	2.517	0.355	Ce2	O225	2.490	0.380	Ce3	O55	2.496	0.375	Ce4	O196	2.532	0.341	Ce5	O239	2.510	0.361
Ce1	O221	2.518	0.354	Ce2	O80	2.500	0.371	Ce3	O141	2.505	0.366	Ce4	O233	2.535	0.339	Ce5	O242	2.528	0.345
Ce1	O222	2.519	0.353	Ce2	O226	2.506	0.365	Ce3	O230	2.508	0.363	Ce4	O237	2.545	0.330	Ce5	O240	2.572	0.308
Ce1	O224	2.532	0.341	Ce2	O228	2.523	0.349	Ce3	O231	2.526	0.347	Ce4	O115	2.550	0.326	Ce5	O243	2.576	0.305
Ce1	O84	2.544	0.331	Ce2	O227	2.525	0.348	Ce3	O229	2.533	0.341	Ce4	O235	2.554	0.323	Ce5	O241	2.581	0.301
Ce1	O206	2.549	0.327	Ce2	O109	2.566	0.313	Ce3	O190	2.547	0.329	Ce4	O236	2.577	0.304	Ce5	O244	2.607	0.282
Ce1	O223	2.588	0.296	Ce2	O133	2.592	0.293	Ce3	O93	2.651	0.251	Ce4	O238	2.583	0.299	Ce5	O245	2.619	0.273
			3.138				3.206				3.164				3.005				2.936

Compound 5

Bond		R	BVS	Bond		R	BVS	Bond		R	BVS	Bond		R	BVS	Bond		R	BVS
Mo1	O157	1.682	1.927	Mo2	O148	1.658	2.080	Mo3	O135	1.699	1.826	Mo4	O119	1.714	1.740	Mo5	O69	1.675	1.971
Mo1	O86	1.946	0.831	Mo2	O48	1.962	0.790	Mo3	O78	1.954	0.810	Mo4	O3	1.987	0.730	Mo5	O34	1.959	0.798
Mo1	O38	1.971	0.768	Mo2	O78	1.973	0.763	Mo3	O48	1.962	0.790	Mo4	O17	1.990	0.723	Mo5	O101	1.972	0.765
Mo1	O106	2.067	0.565	Mo2	O6	2.018	0.661	Mo3	O8	2.064	0.571	Mo4	O106	2.071	0.558	Mo5	O3	2.080	0.543
Mo1	O8	2.077	0.548	Mo2	O22	2.031	0.634	Mo3	O29	2.117	0.482	Mo4	O8	2.086	0.532	Mo5	O86	2.105	0.501
Mo1	O29	2.145	0.441	Mo2	O33	2.259	0.307	Mo3	O23	2.132	0.460	Mo4	O23	2.130	0.463	Mo5	O11	2.218	0.350
			5.081				5.235				4.939				4.746				4.927
Mo6	O67	1.685	1.909	Mo7	O156	1.703	1.802	Mo8	O1	1.681	1.933	Mo9	O161	1.679	1.946	Mo10	O117	1.715	1.735
Mo6	O34	1.972	0.765	Mo7	O45	1.968	0.775	Mo8	O3	1.983	0.739	Mo9	O18	1.977	0.753	Mo10	O39	1.804	1.307
Mo6	O101	1.980	0.746	Mo7	O41	1.970	0.770	Mo8	O138	2.006	0.687	Mo9	O118	1.996	0.709	Mo10	O138	1.824	1.226
Mo6	O99	2.080	0.543	Mo7	O17	2.063	0.573	Mo8	O17	2.007	0.685	Mo9	O24	2.022	0.653	Mo10	O11	2.012	0.674
Mo6	O18	2.093	0.521	Mo7	O78	2.122	0.475	Mo8	O32	2.039	0.618	Mo9	O2	2.035	0.626	Mo10	O4	2.024	0.648
Mo6	O4	2.210	0.359	Mo7	O27	2.177	0.398	Mo8	O28	2.195	0.376	Mo9	O25	2.337	0.239	Mo10	O101	2.395	0.199
			4.842				4.793				5.038				4.926				5.789
				Mo12	O131	1.617	2.370	Mo13	O49	1.718	1.699	Mo14	O198	1.693	1.825	Mo15	O181	1.654	2.107
				Mo12	O99	1.991	0.720	Mo13	O158	1.725	1.665	Mo14	O160	1.724	1.670	Mo15	O103	1.969	0.773
Mo11	O165	1.717	1.704	Mo12	O16	2.008	0.682	Mo13	O24	1.832	1.226	Mo14	O125	1.827	1.243	Mo15	O43	1.984	0.737
Mo11	O172	1.750	1.550	Mo12	O39	2.017	0.663	Mo13	O95	1.964	0.840	Mo14	O38	2.205	0.421	Mo15	O14	2.101	0.507
Mo11	O116	1.789	1.386	Mo12	O98	2.022	0.653	Mo13	O25	2.243	0.377	Mo14	O113	2.213	0.411	Mo15	O7	2.116	0.484
Mo11	O104	1.793	1.371	Mo12	O87	2.221	0.346	Mo13	O129	2.317	0.305	Mo14	O48	2.228	0.394	Mo15	O31	2.230	0.336
			6.011				5.435				6.113				5.965				4.944
Mo16	O65	1.696	1.843	Mo17	O96	1.700	1.789	Mo18	O213	1.693	1.825	Mo19	O126	1.722	1.697				
Mo16	O7	1.949	0.823	Mo17	O32	1.789	1.386	Mo18	O79	1.710	1.738	Mo19	O2	1.790	1.366	Mo20	O133	1.718	1.699
Mo16	O42	1.966	0.780	Mo17	O111	1.817	1.279	Mo18	O188	1.810	1.305	Mo19	O10	1.820	1.242	Mo20	O87	1.723	1.675
Mo16	O92	2.066	0.567	Mo17	O13	1.993	0.773	Mo18	O84	2.203	0.423	Mo19	O4	2.053	0.591	Mo20	O121	1.806	1.320
Mo16	O26	2.074	0.553	Mo17	O27	2.042	0.671	Mo18	O179	2.214	0.410	Mo19	O11	2.063	0.573	Mo20	O25	1.814	1.290
Mo16	O36	2.140	0.448	Mo17	O45	2.396	0.244	Mo18	O209	2.216	0.408	Mo19	O34	2.334	0.242	Mo20	O95	2.478	0.193
			5.015				6.142				6.110				5.710				6.177
Mo21	O147	1.716	1.709	Mo22	O153	1.708	1.774	Mo23	O55	1.700	1.789	Mo24	O140	1.683	1.921	Mo25	O132	1.702	1.779
Mo21	O51	1.787	1.394	Mo22	O105	1.965	0.783	Mo23	O20	1.766	1.481	Mo24	O103	1.973	0.763	Mo25	O98	1.805	1.324
Mo21	O35	1.799	1.347	Mo22	O14	1.971	0.768	Mo23	O6	1.795	1.363	Mo24	O43	1.977	0.753	Mo25	O109	1.826	1.247
Mo21	O31	2.034	0.687	Mo22	O26	2.084	0.536	Mo23	O27	2.042	0.671	Mo24	O16	2.062	0.575	Mo25	O115	1.989	0.782
Mo21	O115	2.044	0.668	Mo22	O92	2.090	0.526	Mo23	O13	2.050	0.656	Mo24	O12	2.099	0.511	Mo25	O31	1.997	0.764
Mo21	O43	2.324	0.299	Mo22	O30	2.123	0.473	Mo23	O41	2.343	0.283	Mo24	O115	2.231	0.335	Mo25	O103	2.398	0.242
			6.104				4.859				6.244				4.858				6.137
Mo26	O57	1.660	2.067	Mo27	O53	1.676	1.964	Mo28	O187	1.665	2.034	Mo29	O182	1.688	1.891	Mo30	O204	1.694	1.855
Mo26	O7	1.973	0.763	Mo27	O118	1.952	0.816	Mo28	O52	1.991	0.720	Mo29	O19	1.977	0.753	Mo30	O123	1.971	0.768
Mo26	O109	2.000	0.700	Mo27	O18	1.982	0.741	Mo28	O40	2.003	0.693	Mo29	O91	1.982	0.741	Mo30	O163	1.990	0.723
Mo26	O59	2.003	0.693	Mo27	O9	2.074	0.553	Mo28	O111	2.014	0.669	Mo29	O130	2.000	0.700	Mo30	O42	2.072	0.557
Mo26	O42	2.014	0.669	Mo27	O47	2.115	0.485	Mo28	O76	2.033	0.630	Mo29	O146	2.025	0.646	Mo30	O91	2.080	0.543
Mo26	O165	2.200	0.370	Mo27	O107	2.128	0.466	Mo28	O102	2.161	0.419	Mo29	O104	2.308	0.262	Mo30	O151	2.176	0.400
			5.263				5.025				5.167				4.994				4.844
Mo31	O66	1.720	1.708					Mo33	O164	1.707	1.753	Mo34	O137	1.724	1.670	Mo35	O178	1.682	1.884
Mo31	O128	1.803	1.311					Mo33	O166	1.714	1.719	Mo34	O76	1.802	1.336	Mo35	O186	1.706	1.759
Mo31	O59	1.832	1.195	Mo32	O63	1.721	1.685	Mo33	O146	1.848	1.171	Mo34	O202	1.837	1.208	Mo35	O162	1.809	1.309
Mo31	O151	2.025	0.646	Mo32	O102	1.743	1.582	Mo33	O93	1.970	0.825	Mo34	O110	1.998	0.762	Mo35	O19	2.188	0.442
Mo31	O94	2.041	0.614	Mo32	O143	1.789	1.386	Mo33	O162	2.315	0.307	Mo34	O37	2.015	0.725	Mo35	O83	2.232	0.390
Mo31	O123	2.393	0.200	Mo32	O167	1.804	1.328	Mo33	O104	2.339	0.287	Mo34	O58	2.374	0.259	Mo35	O105	2.238	0.383
			5.675				5.980				6.062				5.960				6.166
Mo36	O183	1.683	1.921	Mo37	O180	1.730	1.654	Mo38	O189	1.674	1.977	Mo39	O168	1.723	1.691	Mo40	O177	1.685	1.909
Mo36	O134	1.972	0.765	Mo37	O130	1.805	1.303	Mo38	O154	1.993	0.716	Mo39	O134	1.961	0.793	Mo40	O175	1.994	0.713
Mo36	O44	1.991	0.720	Mo37	O141	1.809	1.286	Mo38	O174	2.003	0.693	Mo39	O44	1.962	0.790	Mo40	O58	1.996	0.709
Mo36	O88	2.010	0.678	Mo37	O94	2.026	0.644	Mo38	O202	2.006	0.687	Mo39	O112	2.056	0.586	Mo40	O52	2.071	0.558
Mo36	O20	2.043	0.610	Mo37	O151	2.058	0.582	Mo38	O128	2.032	0.632	Mo39	O142	2.078	0.546	Mo40	O124	2.074	0.553
Mo36	O143	2.291	0.277	Mo37	O163	2.329	0.245	Mo38	O145	2.192	0.380	Mo39	O201	2.151	0.433	Mo40	O37	2.222	0.345
			4.972				5.714				5.085				4.838				4.788

Compound 5 (continued)

Mo41	O207	1.647	2.154	Mo42	O197	1.717	1.704	Mo43	O152	1.705	1.791	Mo44	O193	1.732	1.632	Mo45	O194	1.713	1.746
Mo41	O163	1.993	0.716	Mo42	O75	1.721	1.685	Mo43	O40	1.986	0.732	Mo44	O173	1.802	1.336	Mo45	O70	1.949	0.823
Mo41	O123	2.013	0.672	Mo42	O192	1.853	1.154	Mo43	O52	2.006	0.687	Mo44	O136	1.817	1.279	Mo45	O124	1.992	0.718
Mo41	O174	2.066	0.567	Mo42	O50	1.946	0.884	Mo43	O150	2.071	0.558	Mo44	O110	2.040	0.675	Mo45	O112	2.075	0.551
Mo41	O185	2.077	0.548	Mo42	O155	2.277	0.342	Mo43	O112	2.078	0.546	Mo44	O37	2.045	0.666	Mo45	O150	2.092	0.522
Mo41	O94	2.221	0.346	Mo42	O167	2.356	0.273	Mo43	O142	2.085	0.534	Mo44	O175	2.319	0.304	Mo45	O201	2.129	0.464
			5.003				6.042				4.848				5.892				4.825
Mo46	O77	1.684	1.873	Mo47	O205	1.662	2.054					Mo49	O196	1.692	1.867	Mo50	O82	1.658	2.080
Mo46	O210	1.699	1.794	Mo47	O70	1.950	0.821					Mo49	O175	1.963	0.788	Mo50	O179	1.988	0.727
Mo46	O155	1.810	1.305	Mo47	O124	1.977	0.753	Mo48	O195	1.722	1.680	Mo49	O58	1.975	0.758	Mo50	O185	1.997	0.707
Mo46	O44	2.208	0.417	Mo47	O136	2.004	0.691	Mo48	O145	1.729	1.646	Mo49	O154	2.067	0.565	Mo50	O141	1.998	0.704
Mo46	O73	2.217	0.407	Mo47	O192	2.005	0.689	Mo48	O200	1.796	1.359	Mo49	O62	2.078	0.546	Mo50	O206	2.021	0.655
Mo46	O70	2.262	0.357	Mo47	O167	2.273	0.293	Mo48	O60	1.801	1.339	Mo49	O110	2.225	0.342	Mo50	O60	2.296	0.273
			6.154				5.301				6.024				4.866				5.146
Mo51	O199	1.626	2.303	Mo52	O85	1.729	1.646					Mo54	O120	1.732	1.632	Mo55	O46	1.715	1.714
Mo51	O174	1.963	0.788	Mo52	O208	1.741	1.591					Mo54	O90	1.752	1.541	Mo55	O21	1.728	1.651
Mo51	O154	1.984	0.737	Mo52	O206	1.832	1.226	Mo53	O68	1.710	1.738	Mo54	O22	1.850	1.164	Mo55	O5	1.868	1.105
Mo51	O81	2.066	0.567	Mo52	O191	1.964	0.840	Mo53	O28	1.730	1.642	Mo54	O89	1.945	0.887	Mo55	O89	1.939	0.902
Mo51	O169	2.098	0.512	Mo52	O188	2.284	0.336	Mo53	O33	1.801	1.339	Mo54	O125	2.287	0.333	Mo55	O125	2.303	0.318
Mo51	O54	2.109	0.495	Mo52	O60	2.322	0.301	Mo53	O100	1.820	1.268	Mo54	O33	2.379	0.256	Mo55	O100	2.310	0.312
			5.402				5.939				5.988				5.813				6.002
Mo56	O56	1.672	1.990	Mo57	O122	1.667	2.021	Mo58	O171	1.656	2.094	Mo59	O159	1.714	1.740	Mo60	O64	1.668	2.015
Mo56	O38	1.974	0.760	Mo57	O12	1.970	0.770	Mo58	O45	1.972	0.765	Mo59	O97	1.943	0.839	Mo60	O14	1.962	0.790
Mo56	O86	1.974	0.760	Mo57	O97	1.983	0.739	Mo58	O41	1.997	0.707	Mo59	O12	1.969	0.773	Mo60	O105	1.987	0.730
Mo56	O5	1.981	0.744	Mo57	O144	2.027	0.642	Mo58	O40	2.071	0.558	Mo59	O9	2.067	0.565	Mo60	O15	2.022	0.653
Mo56	O10	1.987	0.730	Mo57	O51	2.033	0.630	Mo58	O134	2.098	0.512	Mo59	O114	2.089	0.527	Mo60	O35	2.027	0.642
Mo56	O100	2.311	0.260	Mo57	O121	2.292	0.276	Mo58	O13	2.229	0.338	Mo59	O107	2.128	0.466	Mo60	O116	2.280	0.287
			5.244				5.079				4.974				4.911				5.117
Mo61	O176	1.697	1.804	Mo62	O71	1.720	1.689	Mo63	O190	1.706	1.759	Mo64	O61	1.691	1.873	Mo65	O184	1.721	1.685
Mo61	O72	1.730	1.642	Mo62	O139	1.732	1.632	Mo63	O149	1.743	1.582	Mo64	O16	1.976	0.756	Mo65	O170	1.742	1.586
Mo61	O129	1.800	1.343	Mo62	O88	1.856	1.144	Mo63	O15	1.858	1.138	Mo64	O99	1.985	0.734	Mo65	O144	1.844	1.184
Mo61	O108	2.189	0.441	Mo62	O50	1.909	0.983	Mo63	O93	1.913	0.972	Mo64	O47	2.045	0.607	Mo65	O95	1.936	0.910
Mo61	O118	2.218	0.406	Mo62	O155	2.291	0.329	Mo63	O162	2.294	0.326	Mo64	O114	2.049	0.599	Mo65	O129	2.279	0.340
Mo61	O97	2.237	0.384	Mo62	O143	2.335	0.290	Mo63	O116	2.356	0.273	Mo64	O9	2.075	0.551	Mo65	O121	2.302	0.319
			6.020				6.068				6.049				5.119				6.024
Mo66	O127	1.698	1.831	Mo67	O203	1.679	1.946	Mo68	O215	1.702	1.779	Mo69	O212	1.668	2.015	Mo70	O211	1.701	1.814
Mo66	O19	1.955	0.808	Mo67	O84	1.977	0.753	Mo68	O214	1.728	1.651	Mo69	O84	1.983	0.739	Mo70	O179	1.969	0.773
Mo66	O91	1.961	0.793	Mo67	O62	1.978	0.751	Mo68	O74	1.807	1.317	Mo69	O62	2.001	0.698	Mo70	O185	1.971	0.768
Mo66	O92	2.067	0.565	Mo67	O54	2.045	0.607	Mo68	O191	1.931	0.923	Mo69	O173	2.013	0.672	Mo70	O169	2.045	0.607
Mo66	O30	2.134	0.457	Mo67	O81	2.082	0.539	Mo68	O188	2.315	0.307	Mo69	O74	2.033	0.630	Mo70	O54	2.064	0.571
Mo66	O36	2.149	0.436	Mo67	O80	2.139	0.450	Mo68	O200	2.334	0.291	Mo69	O200	2.299	0.270	Mo70	O80	2.106	0.499
			4.890				5.045				6.267				5.024				5.031
Nd1	O139	2.441	0.402	Nd2	O231	2.400	0.449	Nd3	O180	2.436	0.408	Nd4	O170	2.439	0.404	Nd5	O126	2.454	0.388
Nd1	O55	2.451	0.391	Nd2	O193	2.449	0.394	Nd3	O234	2.440	0.403	Nd4	O240	2.458	0.384	Nd5	O250	2.468	0.374
Nd1	O158	2.474	0.368	Nd2	O214	2.455	0.387	Nd3	O208	2.484	0.358	Nd4	O149	2.470	0.372	Nd5	O246	2.482	0.360
Nd1	O90	2.478	0.364	Nd2	O232	2.460	0.382	Nd3	O237	2.500	0.343	Nd4	O147	2.490	0.352	Nd5	O245	2.489	0.353
Nd1	O223	2.488	0.354	Nd2	O227	2.500	0.343	Nd3	O233	2.514	0.330	Nd4	O243	2.492	0.350	Nd5	O21	2.511	0.333
Nd1	O221	2.493	0.350	Nd2	O75	2.516	0.329	Nd3	O166	2.521	0.324	Nd4	O241	2.517	0.328	Nd5	O248	2.513	0.331
Nd1	O224	2.506	0.337	Nd2	O230	2.520	0.325	Nd3	O238	2.530	0.316	Nd4	O242	2.529	0.317	Nd5	O247	2.527	0.319
Nd1	O184	2.511	0.333	Nd2	O228	2.547	0.302	Nd3	O236	2.550	0.300	Nd4	O120	2.535	0.312	Nd5	O249	2.552	0.298
Nd1	O222	2.541	0.307	Nd2	O229	2.569	0.285	Nd3	O235	2.570	0.284	Nd4	O239	2.578	0.278	Nd5	O49	2.598	0.263
			3.207				3.195				3.066				3.098				3.020

7. Structure descriptions

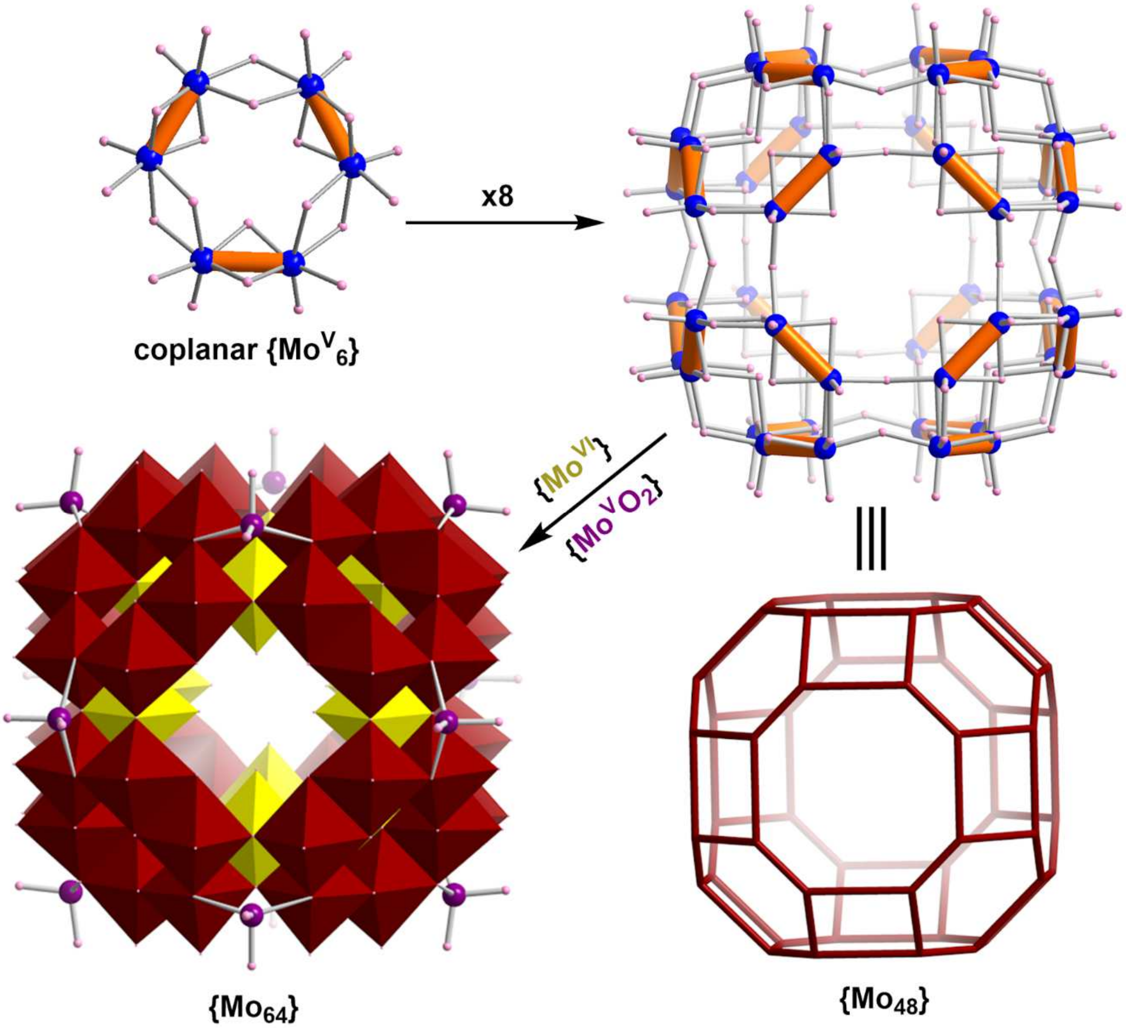

Figure S3. Building block approach towards the formation of the new POM $\{Mo_{64}\}$ super cube structure in compounds **1**-**3**: 48 Mo^{V} atoms form the main frame of the structure in the shape of a truncated cuboctahedron with thick bonds indicating the Mo-Mo bonds of ~2.58 Å in the hexagonal coplanar $\{Mo^{V}_{6}\}$ units; Other two close Mo…Mo separations are 3.57 and 3.89 Å (the rectangle). The $\{Mo^{VI}\}$ units (yellow polyhedrons) locate inside the cage behind the rectangle windows, the cube edge centres. Four $\{Mo^{V}O_2\}$ units are disordered over twelve sites (purple) in front the rectangle windows.

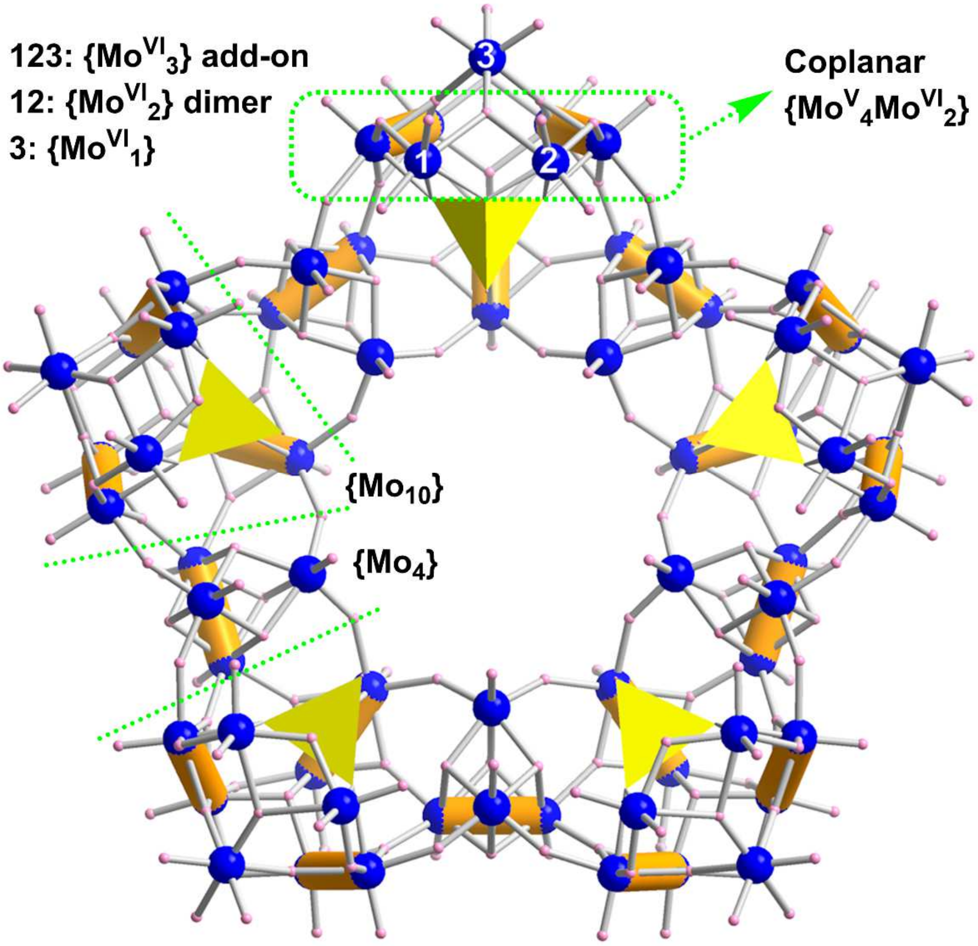

Figure S4. The constituents of the $\{Mo_{70}\}$ star structure, indicating the $\{Mo_{10}\}$ and $\{Mo_4\}$ building blocks, $\{Mo^{VI}_3\}$ add-on group, $\{Mo^{VI}_2\}$ dimer and coplanar $\{Mo^{V}_4Mo^{VI}_2\}$ subunit. $\{Mo^{VI}O_4\}$ templates are indicated as yellow tetrahedron.

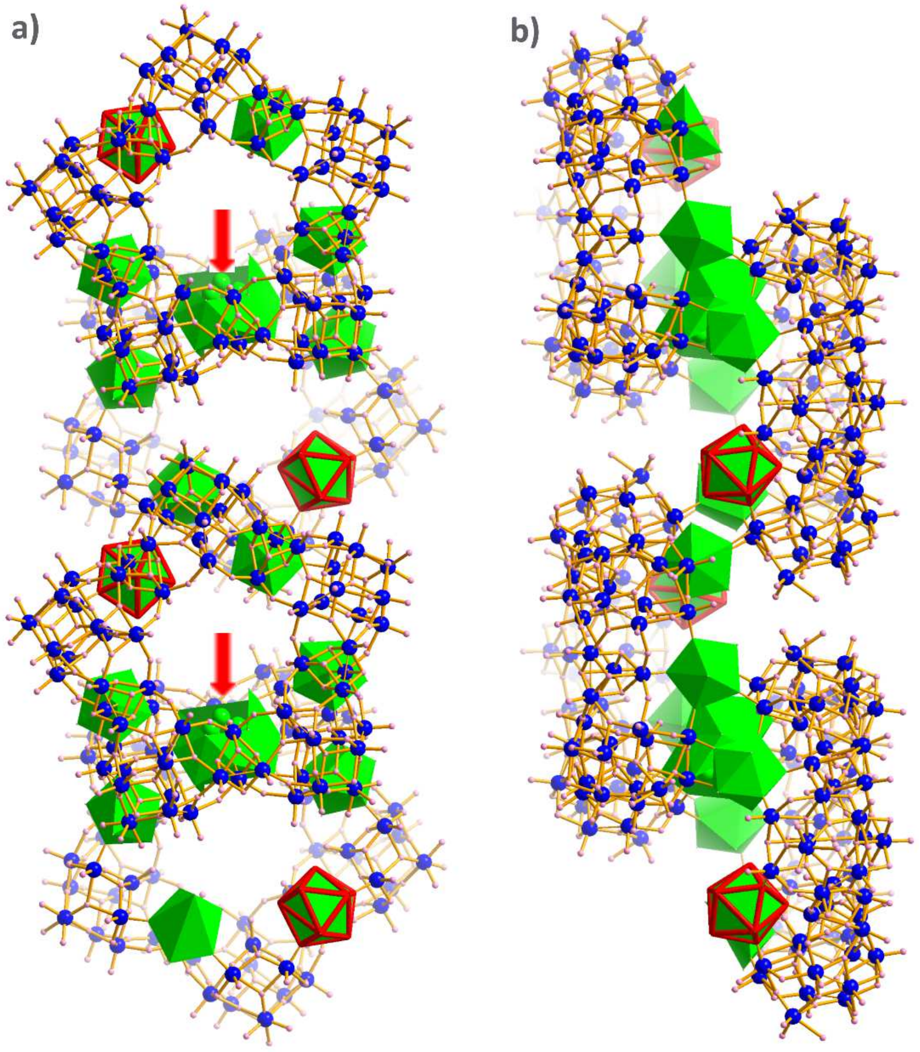

Figure S5a. The 1D chain of {Mo_{70}} star clusters formed by Ce^{3+} ion bridges (green polyhedrons without borders) in compound **4**. The green polyhedrons with red borders are Ce^{3+} ions not forming bridges between clusters. Red arrow points to the inversion centre around which a disordered Ce^{3+} ion is located. a) down view and b) side view of the chain. Colour scheme: Mo/blue, Ce/green, O/purple.

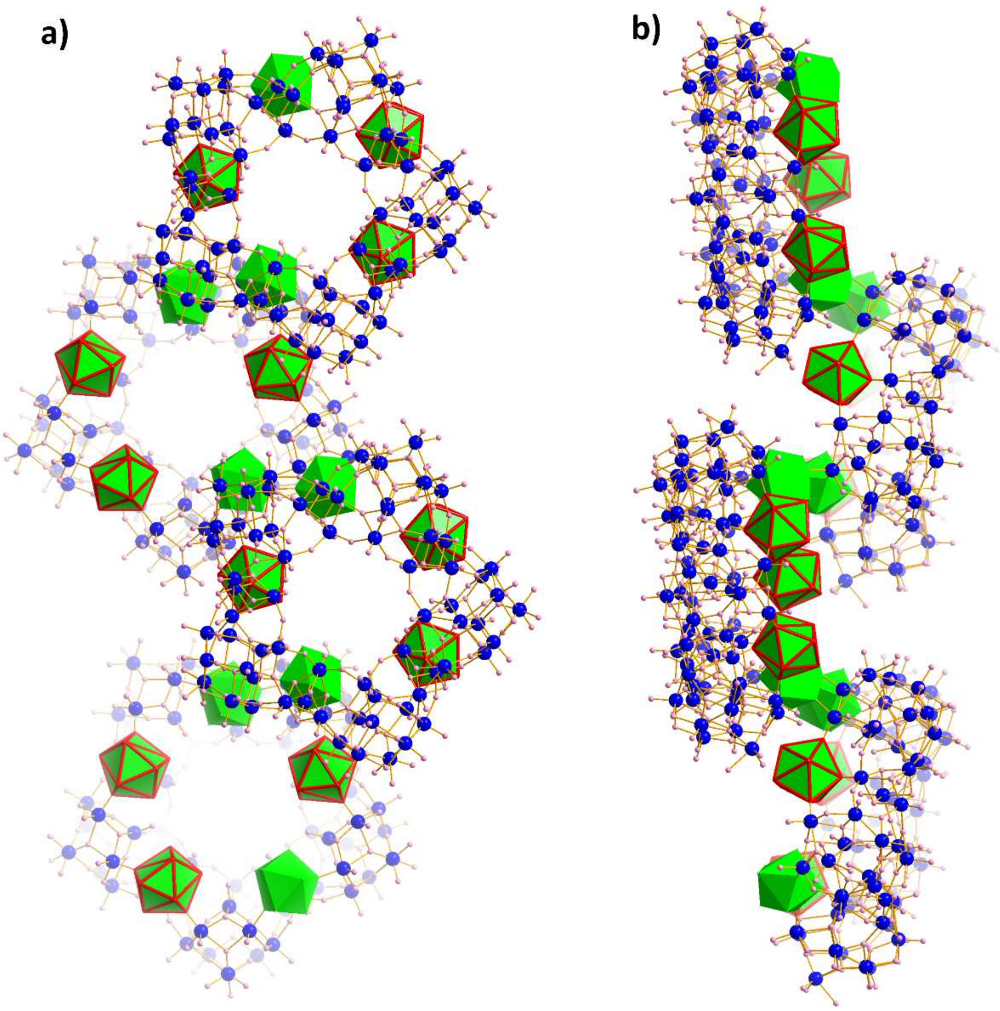

Figure S5b. The 1D chain of {Mo_{70}} star clusters formed by Nd^{3+} ion bridges (green polyhedrons without borders) in compound **5**. The green polyhedrons with red borders are Nd^{3+} ions not forming bridges between clusters. a) down view and b) side view of the chain. Colour scheme: Mo/blue, Nd/green, O/purple.

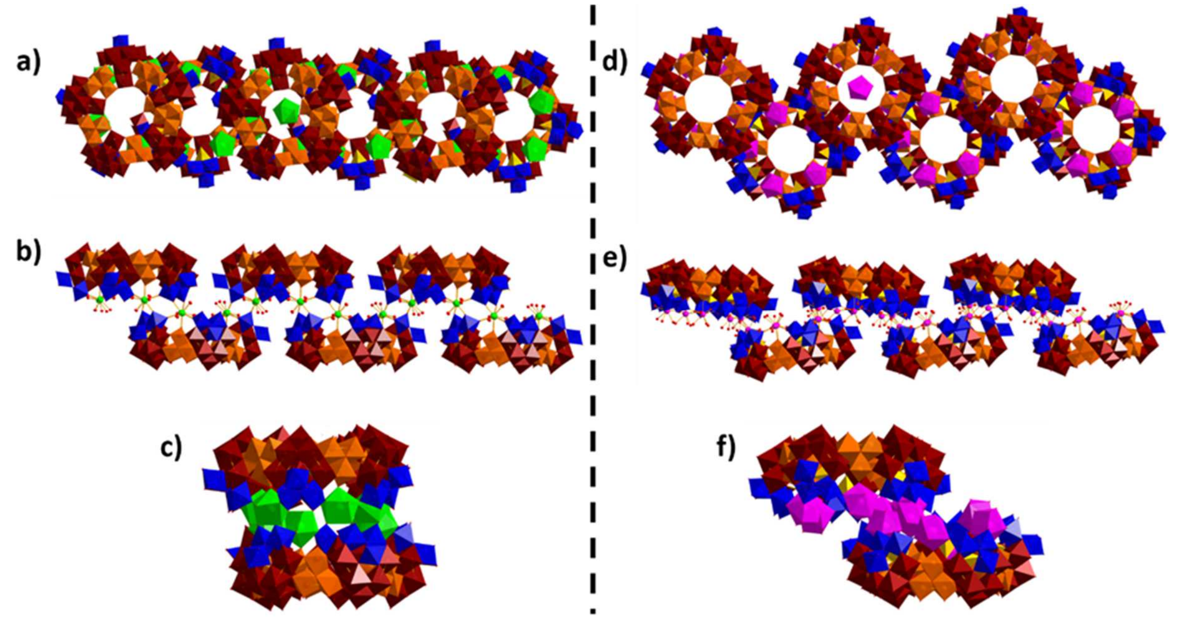

Figure S6. Polyhedron comparison between the crystal packings of compounds **4** and **5**, as seen from a top view, (a) and (d), side view, (b) and (e). The binding between two adjacent frameworks can be seen in (c) and (f).

Table S5. List of representative reported reduced polyoxomolybdates in the order of reduced ratio

Composition	Notation	Reduced ratio	Structure type	Ref.
$[Mo^{V}_{28}Mo^{VI}_{126}H_{14}O_{462}(H_2O)_{70}]^{14-}$	$\{Mo_{154}\}$	0.18	blue wheel	[9]
$[Mo^{V}_{112}Mo^{VI}_{256}H_{16}O_{1032}(H_2O)_{240}(SO_4)_{48}]^{48-}$	$\{Mo_{368}\}$	0.30	blue lemon	[10]
$[Mo^{V}_{80}Mo^{VI}_{168}H_{16}O_{720}(H_2O)_{128}]^{16-}$	$\{Mo_{248}\}$	0.32	blue cage	[11]
$[Mo^{V}_{30}Mo^{VI}_{72}O_{282}(H_2O)_{78}(CH_3CO_2)_{12}]$	$\{Mo_{102}\}$	0.35	blue ball	[12]
$[Mo^{V}_{20}Mo^{VI}_{34}H_4O_{168}(CH_3CO_2)_4]^{32-}$	$\{Mo_{54}\}$	0.37	brown loose	[13]
$[Mo^{V}_{20}Mo^{VI}_{26}H_{10}O_{144}(CH_3CO_2)_4]^{26-}$	$\{Mo_{46}\}$	0.43	brown loose	[14]
$[Mo^{V}_{60}Mo^{VI}_{72}O_{372}(H_2O)_{72}(CH_3CO_2)_{30}]^{42-}$	$\{Mo_{132}\}$	0.45	brown ball	[6]
$[Mo^{V}_{40}Mo^{VI}_{30}H_{30}O_{215}]^{20-}$	$\{Mo_{70}\}$	0.57	red star	this work
$[Mo^{V}_{26}Mo^{VI}_{9}H_{14}O_{112}]^{14-}$	$\{Mo_{37}\}$	0.70	red loose	[15]
$[Mo^{V}_{180}Mo^{VI}_{60}H_{60}O_{680-x}(SO_3)_{20-x}(SO_4)_x]^{(80-2x)-}$	$\{Mo_{240}\}$	0.75	red cage	[16]
$[Mo^{V}_{52}Mo^{VI}_{12}H_{26}O_{200}]^{42-}$	$\{Mo_{64}\}$	0.81	red cage	this work
$[Mo^{V}_{36}Mo^{VI}_{6}H_{15}O_{109}(C_5H_9O_4)_7]^{7-}$	$\{Mo_{42}\}$	0.85	red loose	[17]
$[Mo^{v}_{12}H_{12}O_{40}]^{8-}$	$\{Mo_{12}\}$	1.00	red cage	[18]

8. {Mo_{70}} star VS {Mo_{240}} cage

As mentioned in paper, there is a strong resemblance between the star framework (compounds **4**, **5** and **6**) and the pentagonal window of the previously reported {Mo_{240}} cage (Figure 4). Both share the same two building blocks the tripodal {Mo^{V}_{6}} and tetrahedral {$Mo^{V}_{2}Mo^{VI}_{2}$} that conform the overall framework and, aside from the {Mo^{VI}_{3}} finishing groups and lanthanide ions, present very few other differences. As such it was unexpected when our analyses suggested that one of those differences came in the core of the tripodal {Mo_6} building block. For the case of the star framework, there is little doubt that the central position of the building block is occupied by a tetrahedral {MoO_4} template unit as corroborated by XRD analysis and BVS.

In the case of the {Mo_{240}} cage,[16] the authors posited not only that that this position is occupied by a SO_3^{2-} /SO_4^{2-} template but also that the reduction of SO_4^{2-} to SO_3^{2-} is key for the formation of the overall structure. While this is, in principle, a plausible deduction, the fact the star presents such a resemblance to the cage window portion but with a MoO_4^{2-} in the same position presents a disjunctive. Thus, we argue that the claimed SO_3^{2-}/SO_4^{2-} templates in {Mo_{240}} are most probably MoO_4^{2-} with partial occupancy or a mix of templates, MoO_4/SO_4. The reported SO_3^{2-} is possibly MoO_4^{2-} with the fourth terminal oxo unable to be seen due to very low occupancy. This argument is mainly based on the S-O bond distances determined in the {Mo_{240}} structure. The S-O mean value of 1.697 Å is much closer to the Mo-O bond distances (around 1.77 Å, see Table S6) of MoO_4 templates in the star structures, other than general S-O bond distances (~1.5 Å) found in literature.[19] In the reported {Mo_{240}} structure, S atoms were reported with unusually high thermal parameters.

If the structure data is re-refined with free S occupancies, results show that the total S content is likely not full in these positions but occupied at around 50%, on average. If these S sites are assigned as Mo in the re-refinements, the total occupancy averages 20%. Table S7 lists the polyhedral perimeters of the star structures and {Mo_{240}} as shown in Figure S7, showing how similar the star base frameworks in both the star {Mo_{70}} and ball {Mo_{240}}.

Table S6. Mo-O bond distances of MoO_4 template in the star structures and S-O bond distances in $\{Mo_{240}\}$.

**Compound 4**			**Compound 5**			**Compound 6**			**{Mo240}**		
Mo24	O162	1.734	Mo11	O165	1.717	Mo19	O77	1.720			
Mo24	O83	1.744	Mo11	O172	1.750	Mo19	O131	1.740			
Mo24	O1	1.804	Mo11	O116	1.789	Mo19	O13	1.799			
Mo24	O66	1.822	Mo11	O104	1.793	Mo19	O50	1.861			
Mo25	O69	1.715	Mo20	O133	1.718	Mo27	O20	1.710			
Mo25	O125	1.739	Mo20	O87	1.723	Mo27	O110	1.720			
Mo25	O21	1.837	Mo20	O121	1.806	Mo27	O12	1.831			
Mo25	O19	1.844	Mo20	O25	1.814	Mo27	O70	1.830			
Mo45	O119	1.725	Mo32	O63	1.721	Mo30	O167	1.690			
Mo45	O164	1.745	Mo32	O102	1.744	Mo30	O55	1.700			
Mo45	O77	1.805	Mo32	O143	1.789	Mo30	O116	1.820	S1	O23	1.683
Mo45	O62	1.829	Mo32	O167	1.805	Mo30	O169	1.850	S1	O23	1.683
Mo46	O102	1.720	Mo48	O195	1.723	Mo42	O129	1.683	S1	O23	1.683
Mo46	O52	1.726	Mo48	O145	1.728	Mo42	O28	1.821	S2	O8	1.688
Mo46	O68	1.831	Mo48	O200	1.795	Mo42	O34	1.830	S2	O8	1.688
Mo46	O39	1.834	Mo48	O60	1.801	Mo42	O199	1.840	S2	O33	1.691
Mo59	O182	1.711	Mo53	O68	1.710	Mo65	O47	1.750	S3	O8	1.712
Mo59	O105	1.738	Mo53	O28	1.730	Mo65	O125	1.760	S3	O8	1.712
Mo59	O131	1.821	Mo53	O33	1.801	Mo65	O119	1.790	S3	O9	1.718
Mo59	O180	1.836	Mo53	O100	1.821	Mo65	O194	1.870	S3	O33	1.716
Mean		1.778			1.764			1.781			1.697

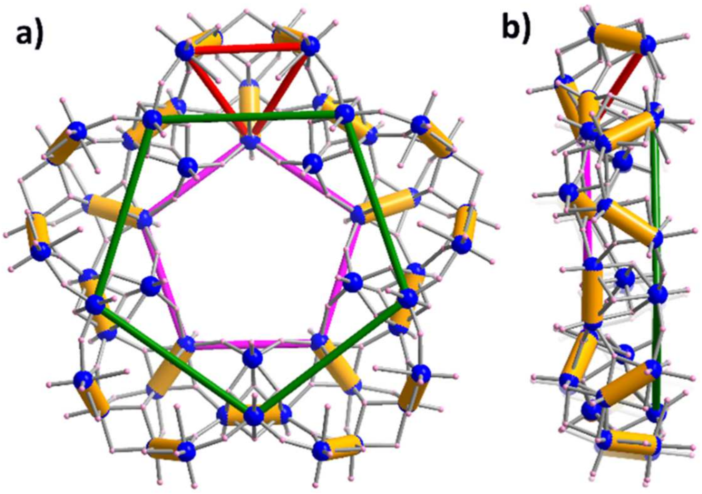

Figure S7. The 'star base', common part for building $\{Mo_{240}\}$ and $\{Mo_{70}\}$. a) Tripod base cycle is the triangle (red) formed by the outer Mo atoms of each pod; The window cycle (pink) is defined by five Mo atoms each from a tripod; the rim cycle (green) is defined by five Mo atoms each from the upper Mo atom of the $\{Mo_4\}$ unit. b) side view of the 'star base' indicating the positions of three cycles.

Table S7. Mo···Mo distances and cycle perimeters for comparison between the stars and Mo_{240}.†

**Compound 4**			**Compound 5**			**Compound 6**			**{Mo240}**		
Tripod base cycle											
Mo48	Mo42	5.929	Mo2	Mo8	5.963	Mo13	Mo29	5.947	Mo4	Mo11	6.027
Mo42	Mo19	5.948	Mo8	Mo56	5.918	Mo29	Mo8	5.964	Mo11	Mo4	6.027
Mo19	Mo48	5.919	Mo56	Mo2	5.945	Mo8	Mo13	5.918	Mo4	Mo4	6.027
Perimeter		17.796			17.826			17.829			18.081
Mo38	Mo44	5.938	Mo12	Mo9	5.904	Mo38	Mo60	5.968	Mo8	Mo8	5.994
Mo44	Mo11	5.941	Mo9	Mo57	5.965	Mo60	Mo63	5.963	Mo8	Mo8	5.994
Mo11	Mo38	5.969	Mo57	Mo12	5.905	Mo63	Mo38	5.952	Mo8	Mo8	5.994
Perimeter		17.848			17.774			17.883			17.982
Window cycle											
Mo48	Mo38	6.325	Mo8	Mo12	6.392	Mo13	Mo31	6.386	Mo4	Mo8	6.441
Mo38	Mo36	6.386	Mo12	Mo26	6.345	Mo31	Mo41	6.385	Mo8	Mo11	6.389
Mo36	Mo8	6.337	Mo26	Mo38	6.386	Mo41	Mo33	6.378	Mo11	Mo8	6.389
Mo8	Mo39	6.386	Mo38	Mo28	6.365	Mo33	Mo38	6.368	Mo8	Mo4	6.441
Mo39	Mo48	6.382	Mo28	Mo8	6.385	Mo38	Mo13	6.395	Mo4	Mo4	6.410
Perimeter		31.816			31.873			31.912			32.070
Rim cycle											
Mo33	Mo14	9.197	Mo23	Mo19	9.211	Mo20	Mo17	9.154	Mo6	Mo6	9.057
Mo14	Mo52	9.229	Mo19	Mo21	9.272	Mo17	Mo66	9.178	Mo6	Mo10	9.170
Mo52	Mo41	9.282	Mo21	Mo37	9.156	Mo66	Mo49	9.231	Mo10	Mo3	9.076
Mo41	Mo6	9.164	Mo37	Mo44	9.210	Mo49	Mo26	9.138	Mo3	Mo10	9.076
Mo6	Mo33	9.165	Mo44	Mo23	9.219	Mo26	Mo20	9.260	Mo10	Mo6	9.170
Perimeter		46.037			46.068			45.961			45.549

†Note: {Mo_{240}} has only two crystallographically unique tripod bases; the three star structures each have five unique tripod bases, of which the largest and smallest perimeter ones are listed.

9. Thermogravimetric analysis (TGA)

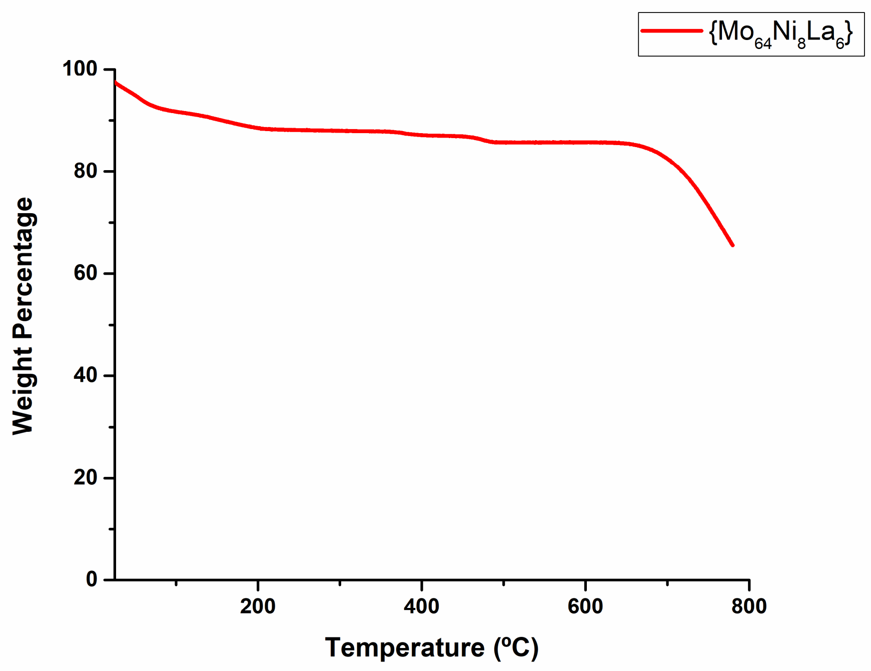

Figure S8. TGA graph of **1**. The weight loss from r.t. to 200 °C is 11.49%, which corresponds to ~86 guest water molecules.

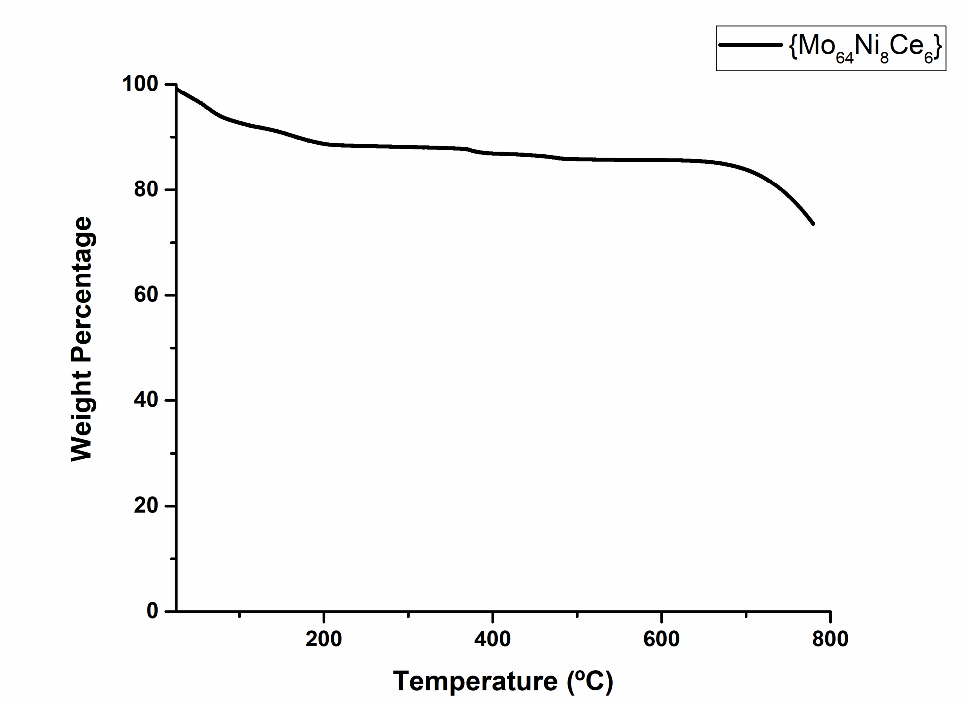

Figure S9. TGA graph of **2**. The weight loss from r.t. to 200 °C is 11.27%, which corresponds to ~84 guest water molecules.

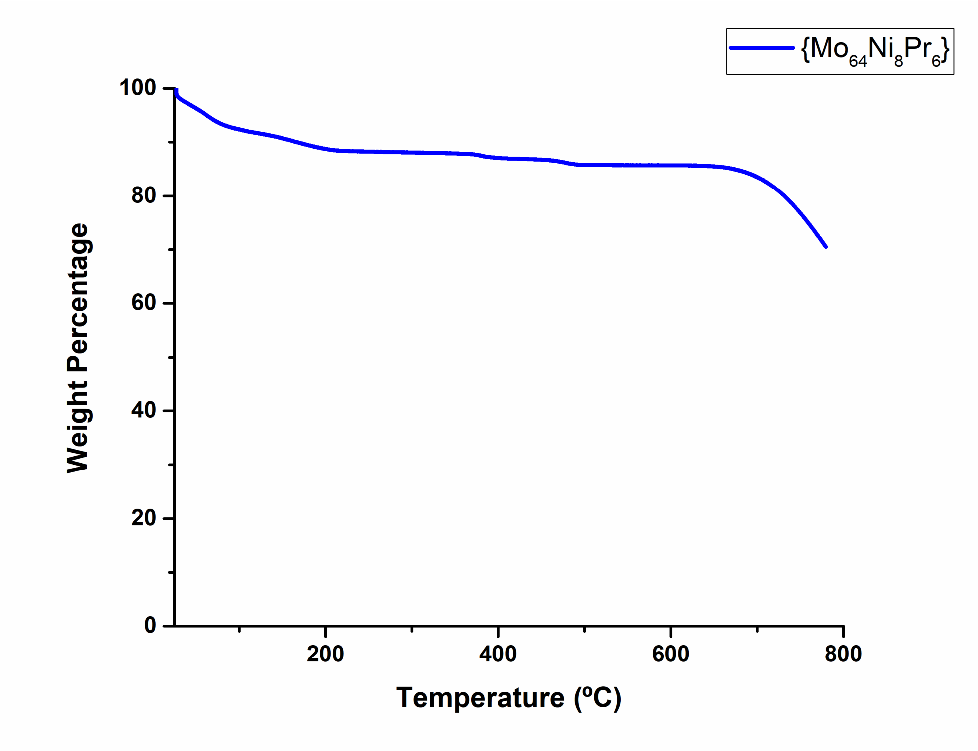

Figure S10. TGA graph of **3**. The weight loss from r.t. to 200 °C is 11.35%, which corresponds to ~85 guest water molecules.

10. Infrared spectroscopy (IR)

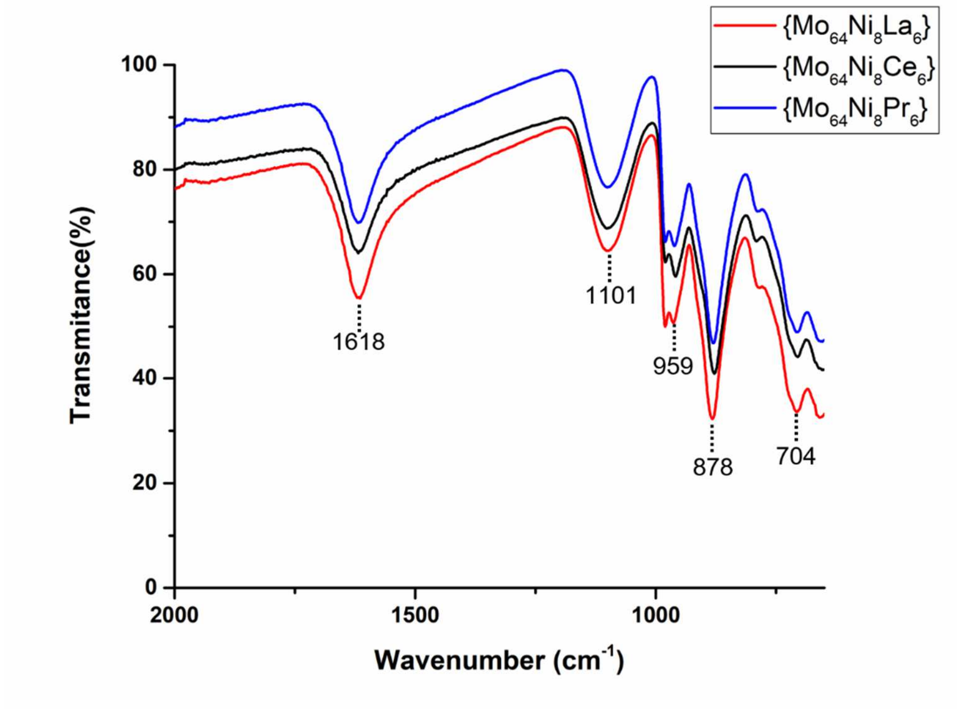

Figure S11. IR spectra for compound **1-3**.

11. UV-VIS spectrums

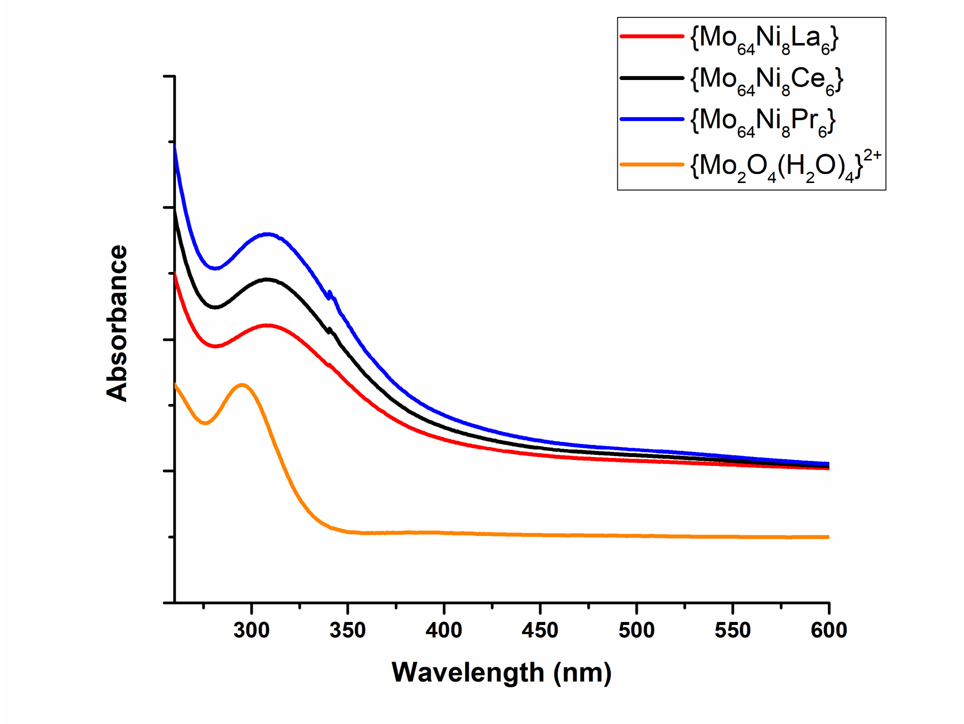

Figure S12. UV spectra for compound **1-3** and $\{Mo_2O_4(H_2O)_4\}^{2+}$ solution.

12. References

[1] G. M. Sheldrick, http://shelx.uni-ac.gwdg.de/SHELX/index.php, **2019**.

[2] L. J. B. Oleg V. Dolomanov, Richard J. Gildea, Judith A. K. Howarda and Horst Puschmanna, *J. Appl. Cryst.* **2009**, *42*, 339-341.

[3] L. Farrugia, *J. Appl. Cryst.* **1999**, *32*, 837-838.

[4] R. C. C. a. J. S. Reid, *Acta Cryst. A* **1995**, *A51*, 887-897.

[5] I. D. Brown, in *Industrial Chemistry Library, Vol. 2* (Eds.: M. O'Keeffe, A. Navrotsky), Elsevier, **1981**, pp. 1-30.

[6] A. Müller, E. Krickemeyer, H. Bögge, M. Schmidtmann, F. Peters, *Angew. Chem. Int. Ed.* **1998**, *37*, 3359-3363.

[7] A. Müller, P. Kögerler, C. Kuhlmann, *Chem. Commun.* **1999**, 1347-1358.

[8] O. C. Gagne, F. C. Hawthorne, *Acta Cryst. B* **2015**, *B71*, 562-578.

[9] T. Liu, E. Diemann, H. Li, A. W. Dress, A. Müller, *Nature* **2003**, *426*, 59-62.

[10] A. Müller, E. Beckmann, H. Bögge, M. Schmidtmann, A. Dress, *Angew. Chem. Int. Ed.* **2002**, *41*, 1162-1167.

[11] A. Müller, S. Q. N. Shah, H. Bögge, M. Schmidtmann, *Nature* **1999**, *397*, 48-50.

[12] A. Müller, S. Q. N. Shah, H. Bögge, M. Schmidtmann, P. Kögerler, B. Hauptfleisch, S. Leiding, K. Wittler, *Angew. Chem. Int. Ed.* **2000**, *39*, 1614-1616.

[13] W. Yang, C. Lu, X. Lin, H. Zhuang, *Chem. Commun.* **2000**, 1623-1624.

[14] W. Yang, C. Lu, X. Lin, H. Zhuang, *Inorg. Chem.* **2002**, *41*, 452-454.

[15] A. Müller, J. Meyer, E. Krickemeyer, C. Beugholt, H. Bögge, F. Peters, M. Schmidtmann, P. Kögerler, M. J. Koop, *Chem. – Eur. J.* **1998**, *4*, 1000-1006.
[16] J. Lin, N. Li, S. Yang, M. Jia, J. Liu, X.-M. Li, L. An, Q. Tian, L.-Z. Dong, Y.-Q. Lan, *J. Am. Chem. Soc.* **2020**, *142*, 13982-13988.
[17] M. I. Khan, J. Zubieta, *J. Am. Chem. Soc.* **1992**, *114*, 10058-10059.
[18] A. Müller, C. Beugholt, P. Kögerler, H. Bögge, S. Bud'ko, M. Luban, *Inorg. Chem.* **2000**, *39*, 5176–5177.
[19] T. Vu, A. M. Bond, D. C. R. Hockless, B. Moubaraki, K. S. Murray, G. Lazarev, A. G. Wedd, *Inorg. Chem.* **2001**, *40*, 65-72.
